# Unveiling Epigenetic Molecular Mechanisms in Bone Fracture Risk: Insights From 731 Immune Cells, 1400 Metabolites, and Critical Genetic Pathways

**DOI:** 10.1155/mi/3846184

**Published:** 2026-04-10

**Authors:** Xiaomin Wan, Wuchao Lu, Jizhao Xue, Jiaxin Huang, Weihong Qian, Zhuoyi Hu, Wulin You, Yafeng Zhang

**Affiliations:** ^1^ Department of Orthopaedic Surgery, Wuxi TCM Hospital Affiliated to Nanjing University of Chinese Medicine, Nanjing University of Chinese Medicine, Nanjing, 210023, China, njucm.edu.cn; ^2^ Department of Orthopaedic Surgery, Tongxiang Hospital of Traditional Chinese Medicine, Tongxiang, 314500, China

**Keywords:** fracture, immune cells, metabolites

## Abstract

**Background:**

The aging population and advancements in medical science have heightened the focus on fractures, which affect over 150 million individuals annually, with substantial health and economic consequences.

**Objective:**

This study investigated the potential causal relationships between 731 immune cells, 1400 metabolites, and nine fracture types using Mendelian randomization (MR).

**Methods:**

A combination of bidirectional MR, two‐sample MR, and mediation MR was employed to assess potential causal links. Sensitivity analysis was performed using MR‐PRESSO. Bioinformatics analyses, including functional enrichment and protein–protein interaction (PPI) network analysis, were conducted. Colocalization analysis was used to examine associations between key genes and fractures.

**Results:**

Bidirectional MR identified 7 immune cell subtypes (e.g., B cells, Tregs, and monocytes) and 11 metabolite classes (e.g., lipids, amino acids) with significant MR‐supported associations with fracture risk, with effects varying by skeletal site. Mediation analysis revealed that the increased wrist fracture risk associated with CD28+CD45RA‐CD8br T cells was mediated by 5‐methylthioadenosine (19.6%), while the reduced foot fracture risk linked to CD28‐CD8dim T cells was mediated via the taurine‐to‐cysteine ratio (20.9%). SNP nearest gene integration highlighted enriched pathways related to immune response, cell adhesion, and metabolism. PPI network analysis pinpointed 9 hub genes, six of which (CD8A, PRKACA, IL‐6, ITGB1, ITPR1, and STAT3) showed strong colocalization evidence with fractures. Moreover, DNA methylation at cg09664550 (ITGB1) showed the most significant negative impact on thoracic spine fractures (OR = 1.986), whereas cg18112163 (STAT3) conferred the strongest protective effect against foot fractures (OR = 0.602; all *p*  < 0.05).

**Conclusions:**

The findings suggest that immune cells and metabolites may have genetically predicted effects on fracture risk, with metabolites potentially serving as key mediators. Critical pathways, hub genes, and fracture‐associated SNPs were identified, along with potential epigenetic regulation via methylation sites. These preliminary insights offer novel directions for future research into the underlying mechanisms of fracture risk and intervention.

## 1. Introduction

The aging global population, combined with improved living conditions and medical advances, has increased public and scientific interest in bone health and fractures [1]. From 1990 to 2021, the incidence of fractures, prevalent cases, and years lived with disability have risen significantly [2]. Only one‐third of fracture patients regain full function, and mortality rates increase 4‐ to 7‐fold within the year following a hip fracture [3]. These injuries severely impact quality of life and impose substantial economic burdens [4].

Bone mineral density (BMD) is a well‐established determinant of fracture risk [5]. Numerous diseases are strongly associated with BMD, including nonalcoholic fatty liver disease [6], hyperglycemia [7], diabetes [8], obesity [9], sarcopenia [10], schizophrenia [11], and dementia [12]. Conversely, inverse causal relationships have been observed between BMD and homocysteine [13], LDL‐cholesterol [14], macrophage inflammatory protein [15], and Hemoglobin A1c [16]. Specific lipid metabolites also influence bone health, demonstrating causal effects on heel BMD [17]. Moreover, endogenous adrenal hormones have been shown to increase lumbar spine BMD and reduce fracture risk in women [18].

Immune cells play a critical role in modulating the metabolic microenvironment and facilitating fracture repair. The balance between macrophage polarization (M1/M2) is essential for effective healing [19], while dysregulated immune cell activity—including neutrophil and mast cell dysfunction—can impede the process [20]. The interaction between mesenchymal stem cells and immune cells is vital for coordinating bone injury repair [21]. In elderly hip fracture patients, significant shifts in immune cell subpopulations occur at different healing stages, directly affecting tissue repair, infection resistance, and fracture outcomes [22]. Bone regeneration is a complex, multistage process that requires dynamic interactions between immune and stromal cells to orchestrate tissue reconstruction [23]. Thus, the interplay between immune and metabolic factors is critical for successful bone healing.

Observational studies are limited in their ability to establish causality. While randomized controlled trials (RCTs) are considered the gold standard, their feasibility is often limited. Genome‐wide association studies (GWAS) provide valuable insights into genetic mechanisms, and integrating diverse methodologies is crucial for elucidating causal relationships between immune cells, metabolites, and fractures [24].

Osteoporosis, a systemic skeletal disorder characterized by reduced bone mass, microarchitectural deterioration, and increased fracture risk, exemplifies this complexity [25]. Mendelian randomization (MR) studies have identified intricate causal links between immune phenotypes and osteoporosis, highlighting the immune‐osteoporosis connection [26]. However, previous MR studies on fractures have been limited in scope, typically focusing on isolated immune or metabolic trait sets and single fracture sites [27, 28]. To address this gap systematically, our study encompasses nine fracture sites, 731 immune cell traits, and 1400 metabolites. This scale of data provides a clear coverage advantage, allowing for a more comprehensive investigation of the causal roles of the immune‐metabolic axis in fracture risk.

This study aims to explore the potential causal relationships between immune cells, metabolites, and fracture risk using comprehensive MR methods based on genetic variants. This research is pivotal for advancing fracture prevention, improving skeletal health outcomes, and informing targeted interventions.

## 2. Materials and Methods

### 2.1. Study Design

**Figure 1:**
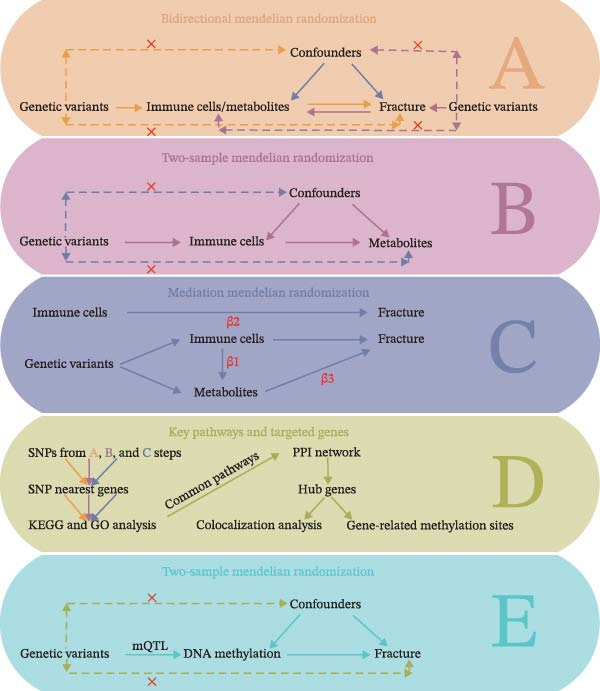
Workflow of the study based on MR analysis. Confounders include both measured and unmeasured factors that could distort the observed association between exposure and outcome. These confounding variables can introduce bias, leading to inaccurate conclusions about the true causal relationship. KEGG: Kyoto Encyclopedia of Genes and Genomes; GO: Gene Ontology; mQTL: DNA methylation quantitative trait loci.

Figure 1 illustrates the study design and the assumptions underlying MR. The MR analysis includes four key components: a bidirectional MR approach to explore potential causal relationships between fractures, immune cells, and metabolites; a two‐sample MR approach to investigate potential causal links between immune cells and metabolites; and mediation MR to examine the pathways involving fractures, immune cells, and metabolites. Additionally, the study aimed to identify the nearest genes associated with causal genetic variants to clarify the pathways and potential targets related to fractures. This research integrates MR analysis of GWAS summary data with DNA methylation quantitative trait loci (mQTL) data to prioritize DNA methylation‐mediated causal variants [29].

### 2.2. Data Sources

#### 2.2.1. Data Sources of Immune Cells, Metabolites, and DNA Methylation

Immune cells and metabolites served as exposure data in the MR analysis. Summary statistics on immune cell phenotypes were retrieved from the GWAS Catalog database using identifiers GCST90001391 to GCST90002121 (Table 1) (https://www.ebi.ac.uk/gwas/studies/GCST90001391-GCST90002121). The cohort study, which included 3757 Sardinian individuals, provided data on 22 million genetic variants related to 731 immune cell phenotypes. These included 118 absolute cell counts (AC), 192 relative counts (RC), 389 median fluorescence intensities (MFI) of surface antigens, and 32 morphological parameters (MP). The study focused on immune cell phenotypes related to MFI, AC, and RC [30].

**Table 1:** GWAS data sets of immune cells, metabolites, and fractures.

GWAS data set	Cases/controls	Sample size	Ethnicity	Source
Exposure/mediators				
731 immunophenotypes	Not relevant	3,757	Sardinian	DOI: 10.1038/s41588‐020‐0684‐4
1400 metabolites	Not relevant	8,299	Canadian	DOI: 10.1038/s41588‐022‐01270‐1
Outcomes				
Fracture of foot, except ankle	8,397/383,369	391,766	European	FinnGen R10
Fracture of forearm	21,495/383,405	404,900	European	FinnGen R10
Fracture of lower leg, including ankle	22,027/352,617	374,644	European	FinnGen R10
Fracture of lumbar spine and pelvis	6,831/398,057	404,888	European	FinnGen R10
Fracture of neck	1,649/404,306	405,955	European	FinnGen R10
Fracture of rib(s), sternum, and thoracic spine	9,995/395,649	405,644	European	FinnGen R10
Fracture of shoulder and upper arm	12,920/376,853	389,773	European	FinnGen R10
Fracture of skull and facial bones	7,580/358,857	366,437	European	FinnGen R10
Fracture at wrist and hand level	12,701/366,724	379,425	European	FinnGen R10

Circulating metabolites were obtained from comprehensive GWAS datasets on the human metabolome, encompassing 1091 blood metabolites and 309 metabolite ratios, analyzed from 8299 participants with ~15.4 million SNPs in the Canadian Longitudinal Study on Aging (CLSA) cohort. Full GWAS summary statistics for these 1400 blood biomarkers are publicly available in the GWAS Catalog (Table 1) (https://www.ebi.ac.uk/gwas/studies/GCST90199621-902010209) [31].

The study also utilized mQTL data regulating DNA methylation sites of interest from the Genetics of DNA Methylation Consortium (GoDMC), which involves over 30,000 participants with genetic and DNA methylation data. The mQTL data is publicly accessible from the resource at http://fileserve.mrcieu.ac.uk/mqtl/assoc_meta_all.csv.gz [32].

#### 2.2.2. GWAS Data Sources for Fractures

Fracture outcome data were sourced from the R10 version of the comprehensive Finnish database (https://www.finngen.fi/) [33]. The study included various fracture types, with the respective case and control sizes detailed in Table 1. Participants of European descent were included, and the study received local ethics approval. No new data collection was involved, and therefore no additional ethical approval was required.

### 2.3. Instrumental Variable (IV) Selection

SNPs associated with immune cell phenotypes and metabolites were selected using a threshold of *p*  < 1 × 10^–5^, with linkage disequilibrium (LD) checks performed (*r* ^2^ = 0.001, kb = 10,000). For inverse MR validation, SNPs linked to nine fracture types were tailored with a stricter threshold (*p*  < 1 × 10^–6^, *r* ^2^ = 0.01, kb = 5000) to ensure sufficient SNPs for analysis. The *r* ^2^ and kb values represent LD between loci, indicating correlated allele frequencies if LD is present. To assess IV strength and mitigate weak instrument bias, the *F*‐statistic was computed for each SNP, excluding those with low *F* values (<10) from the analysis as IVs [34–36].

The study ensured that selected exposures met three key assumptions: (1) Exposure must be correlated with the outcome, (2) IVs must not be associated with confounders influencing the exposure–outcome relationship, and (3) IVs should only affect the outcome through the exposure, not through other mechanisms. Any IVs violating these assumptions were excluded from analysis.

### 2.4. MR Analysis and Sensitivity Analysis

To investigate potential causal relationships between immune cells/metabolites and fractures, the study primarily utilized the TwoSampleMR (Version 0.6.1) and MR‐PRESSO (Version 1.0) packages in *R* (4.4.0). Various MR analysis methods were employed, including inverse variance weighting (IVW), MR‐Egger, weighted median, weighted mode, and MR‐PRESSO [37–40]. IVW was used as the primary method to estimate causality by combining effects from multiple genetic variants, providing a comprehensive causal estimate. Results were presented as odds ratios (OR) with 95% confidence intervals (CI), with significance set at *p*  < 0.05. However, IVW has limitations due to its reliance on specific assumptions and inability to account for horizontal pleiotropy. MR‐Egger [38] and MR‐Presso [39] were used to assess horizontal pleiotropy (with *p*  < 0.05 indicating significance), while Cochran’s Q test evaluated heterogeneity [41]. Unlike MR‐Egger intercept testing, MR‐Presso identifies outliers that fall outside the scope of horizontal pleiotropy [42]. Mediation proportions were calculated using the formula: (β1 × β3)/β2, where β2 represents the total effect, β1 indicates the influence of immune cells on mediators (metabolites), and β3 reflects the impact of mediators on fractures [43]. The β value denotes the effect size of the SNP, with β > 0 indicating a positive correlation and β < 0 indicating a negative correlation. Standard errors and CIs were calculated using delta methods.

### 2.5. Bioinformatics Analysis

After conducting bidirectional MR and two‐sample MR, SNPs causally linked to immune cells/metabolites and fractures were identified. The vautils (Version 0.1.0) package (https://github.com/oyhel/vautils) was used to determine the nearest genes to the SNPs of interest. Specifically, for each SNP, we identified all protein‐coding genes located within a 100‐kb flanking window (50 kb upstream and 50 kb downstream, based on the GRCh37/hg19 assembly). From this set of genes, the one with the shortest absolute physical distance from its transcription start site to the SNP was defined as the primary nearest gene. For SNPs located within gene‐dense regions or where multiple genes fell within the defined flanking window, the three genes with the smallest absolute distances to the SNP were initially retained to ensure comprehensive annotation. The final list of candidate genes was then consolidated by removing duplicate entries across all SNPs.

#### 2.5.1. GO and KEGG Analyses

Functional enrichment analysis was performed using Kyoto Encyclopedia of Genes and Genomes (KEGG) pathways and Gene Ontology (GO) annotations. KEGG enrichment and GO analyses were conducted with the ClusterProfiler (Version 4.12.0) and org.Hs.eg.db (Version 3.19.1) packages. Gene annotations from the KEGG API (https://www.kegg.jp/kegg/rest/keggapi.html) served as the background dataset, with statistical significance defined by an FDR <0.05 [44].

#### 2.5.2. Identification of Key SNPs Nearest‐Genes

A protein–protein interaction (PPI) network was constructed using the STRING database (https://string-db.org/) and visualized in Cytoscape software (Version 3.10.0) [45]. Key genes in the PPI network were identified with the CytoHubba plugin, utilizing four topological analysis methods: degree, edge percolated component (EPC), bottleneck, and closeness [46]. The top 20 nodes were selected, identifying 9 hub genes that intersected across all methods.

#### 2.5.3. Colocalization Analysis

Colocalization analysis was performed with the coloc R package (Version 5.2.3) to assess associations between key genes and fractures. The dataset was sourced from the IEU open GWAS project (https://gwas.mrcieu.ac.uk/) [47]. A Bayesian framework was used to test five mutually exclusive hypotheses:

• H0: No SNP associated with any trait in the region
• H1: A causal SNP linked to the first trait
• H2: A causal SNP linked to the second trait
• H3: A causal SNP linked to both traits
• H4: Presence of two different SNPs, each corresponding to one trait

An a priori probability (p1, p2) for SNP association with only one trait was set at 1 × 10^–4^. If the posterior probability of shared causal variation (PH4) exceeded 0.75 and the probability (p12) of both traits was greater than 1 × 10^–5^, the SNP was considered to show strong evidence of colocalization [48, 49]. This analysis provides insight into the genetic relationship between loci and fractures.

#### 2.5.4. Statistical Analysis: R Packages

Statistical analyses and visualizations were conducted using R Version 4.4.0. The ggplot2 package (Version 3.3.6) was used for bar plots and forest plots, the VennDiagram package (Version 1.7.3) for Venn diagrams, and the ggalluvial package (Version 0.12.5) for stacked bar plots. These tools facilitated the extraction of meaningful insights and conclusions.

## 3. Results

### 3.1. Comprehensive Insights Into Immune and Metabolic Factors Influencing Fracture Risk: Bidirectional MR Analysis of Immunophenotypes/Metabolites for Fracture

This study rigorously analyzed immune cell subpopulations and metabolite classes with MR‐supported associations across multiple fracture types. A total of 731 distinct immune cell subpopulations were assessed, with seven identified as potentially influential in fracture susceptibility, including B cells, regulatory T cells, TBNK cells, various T cell stages, conventional dendritic cells, myeloid cells, and monocytes. The number of immune subtypes causally linked to fractures varied by anatomical location, ranging from 21 for skull/facial fractures to 52 for forearm and lower leg fractures (Figure 2A). This variability highlights the complex interactions among immune system branches in regulating skeletal health.

Figure 2Percentage of immune cell and metabolite subtypes with MR causality for multiple fractures. (A) Distribution of seven immune cell subtypes with MR causality for multiple fractures, including B cells, Treg cells, TBNK cells, various T cell maturation stages, cDC cells, myeloid cells, and monocytes. (B) Distribution of 11 metabolite subtypes with MR causality for multiple fractures, including lipids, metabolite ratios, unknown metabolites, amino acids, xenobiotics, nucleotides, cofactors and vitamins, carbohydrates, partially characterized molecules, peptides, and energy‐related metabolites. (C) Immune cells associated with the shoulder/upper arm in MR analysis. FDR (BH‐adjusted *p*‐value) = *p*× (268/Rank of *p*). Metabolites associated with fractures of the (D) lumbar spine/pelvis and (E) neck in MR analysis. *p*  < 0.05 (Benjamini–Hochberg corrected). FDR (BH‐adjusted *p*‐value) = *P*× (528/rank of *p*). The significant association points (*p*.adj < 0.05) are above the dotted line, and some key markers are marked.

**Figure:**
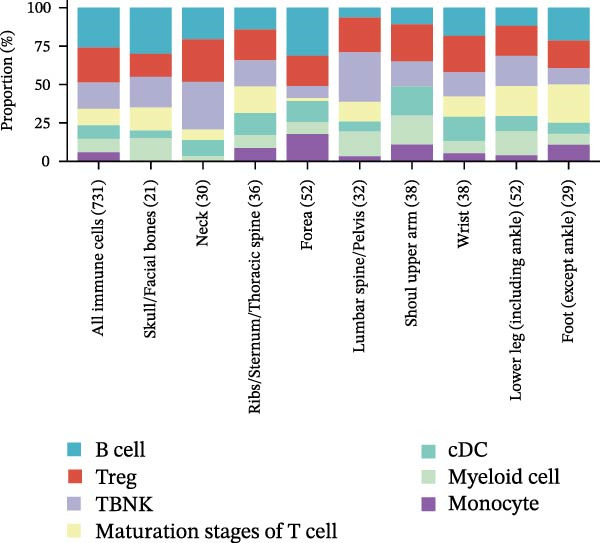
(A)

**Figure:**
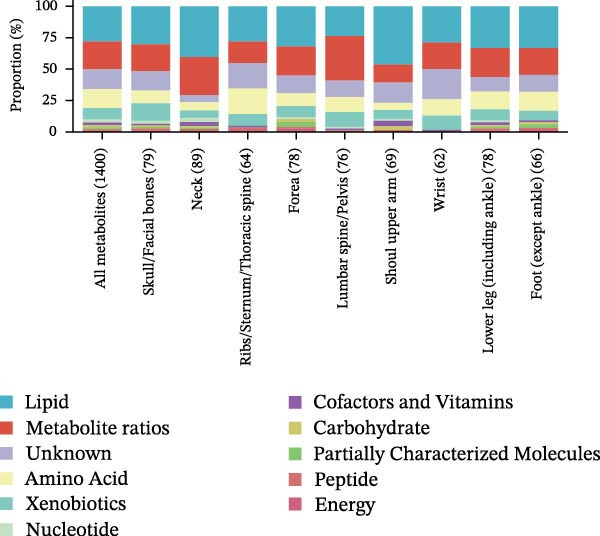
(B)

**Figure:**
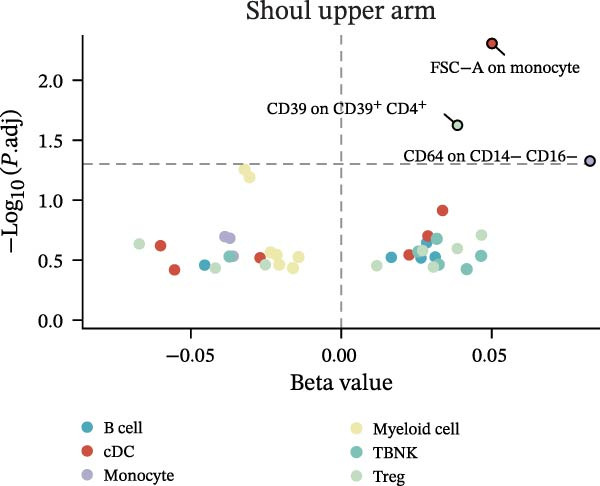
(C)

**Figure:**
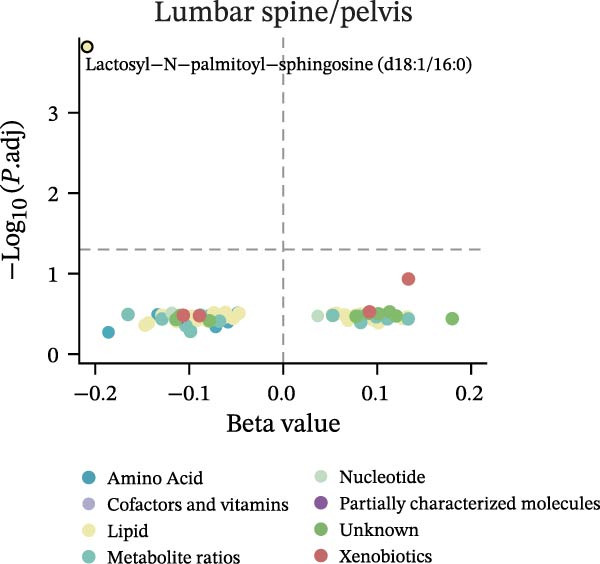
(D)

**Figure:**
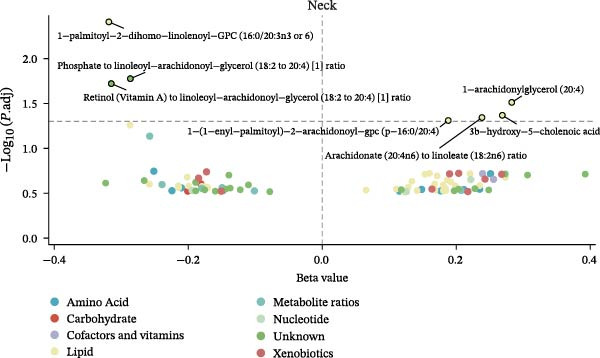
(E)

Similarly, the metabolite analysis evaluated 1400 distinct blood‐borne metabolites, revealing 11 broad categories spanning lipids, amino acids, nucleotides, cofactors/vitamins, carbohydrates, and energy‐related pathways, all influencing fracture risk. Just as with immune cells, specific metabolites associated with fractures varied by location, with 62 metabolites linked to wrist fractures and 89 to neck fractures (Figure 2B). After removing LD, we identified 268 immune cell traits and 528 metabolites that showed at least one MR association with any fracture (Figure 2A, B, Supporting Information 1: Figures S1–S18). To identify robust associations, we applied Benjamini–Hochberg multiple testing correction to the primary MR results (immune cell–fracture and metabolite–fracture) by fracture site. Three immune cell phenotypes were associated with shoulder/upper arm fracture (FSC‐A on monocyte [cDC], CD39 on CD39+ CD4+ [Treg], and CD64 on CD14‐ CD16‐ [Monocyte]) (FDR <0.05, Figure 2C), and eight metabolites were associated with lumbar/pelvic and neck fractures (e.g., 1‐arachidonylglycerol (20:4) [Lipid], and 1‐palmitoyl‐2‐dihomo‐linolenoyl‐GPC (16:0/20:3n3 or 6) [Lipid]) (FDR < 0.05, Figure 2D, E). This study presents the complete results from the IVW analyses after FDR correction (Supporting Information 2: Tables S1, S2).

Among immune cell types, 55 distinct phenotypes were primarily associated with forearm fractures, while 89 metabolites showed strong links to neck fractures. B cells and Tregs exhibited significant associations with multiple fractures, alongside specific lipid metabolites (Figure 3A, B). Notably, B cells and metabolites such as lipids, metabolite ratios, and xenobiotics suggested the most extensive causal relationships with fractures across various anatomical sites. This comprehensive overview highlights the site‐specific and exposure‐specific nature of genetic predisposition to fracture risk (Figure 3C).

Figure 3Summary of immune cells and metabolites with MR associations with fractures. (A) Number of immune cells with MR associations with fractures. (B) Number of metabolites with MR associations with fractures. (C) Heatmap of causal effects of immune cells and metabolites on fractures at different anatomical sites. The heatmap summarizes the inverse variance weighted (IVW) method from Mendelian randomization analysis conducted between nine fracture sites and two exposure categories (immune cells and metabolites). Red indicates a beta value greater than 1, while blue indicates a beta value less than 1. Color intensity corresponds to the number of significant IVW results. All the presented results are statistically significant, with a *p*‐value of less than 0.05.

**Figure:**
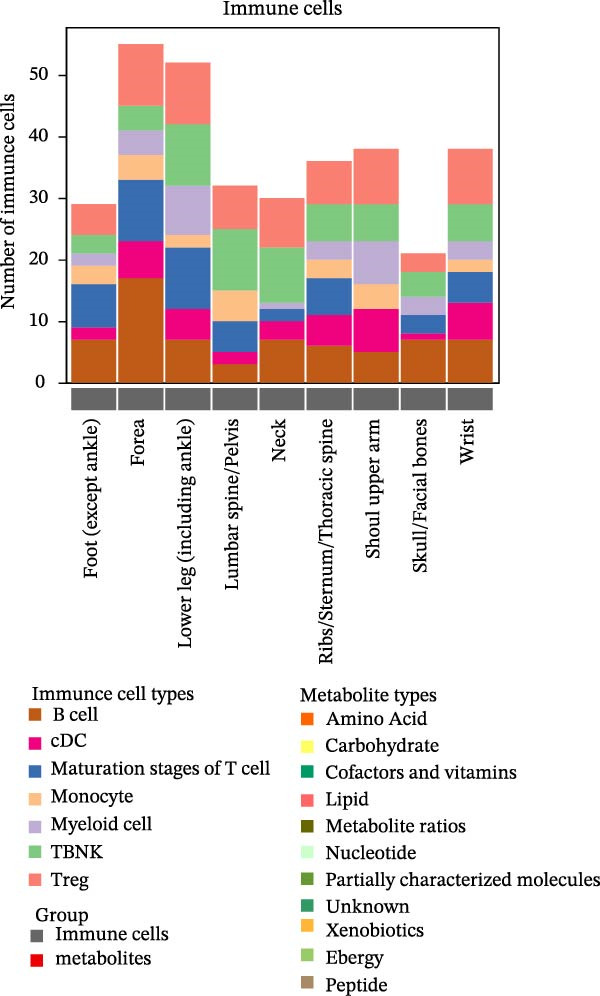
(A)

**Figure:**
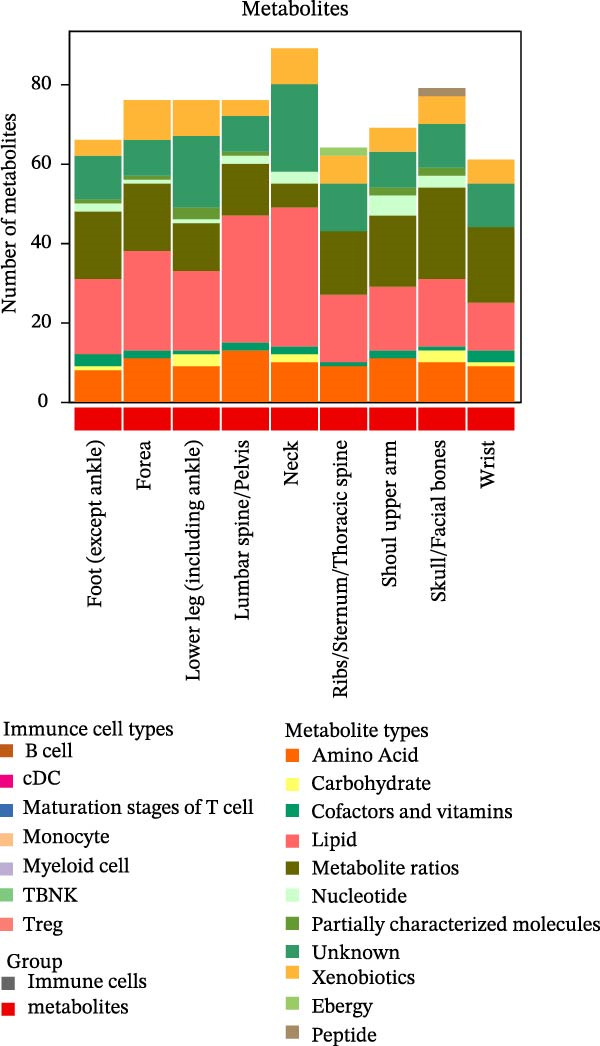
(B)

**Figure:**
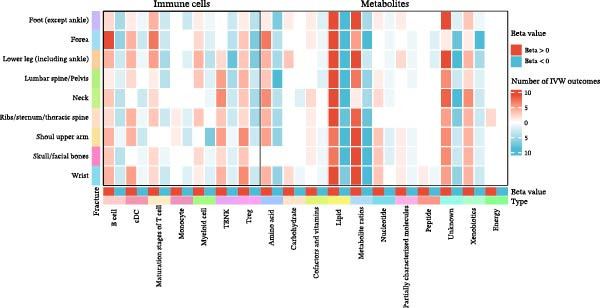
(C)

In assessing horizontal pleiotropy, MR‐Egger and IVW methods were employed. Results showed no evidence of horizontal pleiotropy, and Cochrane Q‐tests indicated minimal heterogeneity (Supporting Information 1: Figures S1–S18). The IVW method confirmed significant causal associations between immune cell phenotypes/metabolites and various fracture types. Horizontal pleiotropy was negligible, as indicated by nonsignificant MR‐Egger intercept tests in 96.7% (956 out of 989) of cases. Furthermore, 97.0% (959 out of 989) of associations exhibited no significant heterogeneity based on Cochran’s Q test. These results support the validity and consistency of the IVs used in our main analyses (Supporting Information 1: Figures S1–S18). Further analyses using MR‐Egger, Weighted Median, Simple Mode, and Weighted Mode methods refined these causal relationships (Supporting Information 2: Tables S3–S20). Sensitivity analysis through leave‐one‐out (LOO) testing indicated strong consistency across genetic instruments, with all results meeting the minimum validity criterion and a substantial proportion meeting the maximum criterion for both immune cell traits and metabolites (Supporting Information 1: Figure S19 A–E). Reverse MR analysis indicated no reverse causality for positively associated immune cells/metabolites, with nearly all *p*‐values >0.05 (Supporting Information 2: Tables S21−S38).

In summary, MR analyses provide evidence consistent with genetically predicted effects of immune cell profiles and metabolites on fracture risk. These findings highlight complex biological mechanisms that potentially link immunity, metabolism, and skeletal health. The variety of influential factors—from immune cell subtypes to diverse metabolite classes—indicates that fracture susceptibility arises from a multifaceted physiological network rather than a single pathway. These results suggest the importance of continued research into the relationships connecting immunity, metabolism, and bone health.

### 3.2. Two‐Sample MR Analysis of Immune Cell Phenotypes and Metabolites Influencing Fracture Risk

To further investigate the connections between immune cells, metabolites, and fractures, our analysis explored the relationships between pre‐identified immune cell subtypes (Supporting Information 1: Figures S1‐–S9) and metabolites associated with fractures (Supporting Information 1: Figures S10–S18). Using the two‐sample MR method, causal associations between immune cells, metabolites, and fractures at nine targeted sites were assessed through the IVW method (Supporting Information 2: Tables S39−S47).

For foot fractures (excluding the ankle), 38 immune cell types associated with increased risk and 37 associated with protection were identified, along with their corresponding metabolites (Supporting Information 2: Table S39). Forearm fractures displayed a pronounced immune and metabolite profile, with 84 immune cell types linked to increased risk and 64 to protection, along with their associated metabolites (Supporting Information 2: Table S40). A similar pattern was observed for lower leg fractures (including the ankle), where 84 immune cell types were associated with increased risk and 72 with protection, alongside corresponding metabolites (Supporting Information 2: Table S41). Lumbar spine/pelvic fractures were linked to 53 risk‐associated and 48 protective immune cell types, each with relevant metabolite profiles (Supporting Information 2: Table S42).

Neck fractures were associated with 48 immune cell types linked to increased risk and 50 associated with protection, along with their metabolites (Supporting Information 2: Table S43). Rib, sternum, and thoracic spine fractures exhibited an equal distribution, with 50 immune cell types linked to both risk and protection, alongside corresponding metabolite profiles (Supporting Information 2: Table S44). Shoulder and upper arm fractures were related to 50 immune cell types associated with increased risk and 60 with protection, each with relevant metabolites (Supporting Information 2: Table S45). Skull and facial fractures were connected to 44 immune cell types linked to increased risk and 28 to protection (Supporting Information 2: Table S46). Finally, wrist fractures were associated with 52 immune cell types linked to increased risk and 46 with protection, along with their metabolites (Supporting Information 2: Table S47).

These metabolites, consistently aligned with MR associations, may serve as potential mediators of immune cell effects on fracture risk. The site‐specific immune cell and metabolite profiles highlight complex relationships between immune systems, metabolic factors, and fracture susceptibility. Different immune cell populations and their associated metabolites likely play distinct roles in modulating fracture risk at various skeletal sites, offering insights for targeted interventions and personalized fracture prevention strategies.

### 3.3. Causal Effects of Possible Mediators on Fracture

Building on the positive results from two‐sample MR (Supporting Information 2: Tables S39−S47), immune cells were selected as exposures and metabolites as mediators to investigate their potential mediating effects on fractures. The overall influence of metabolites as mediators in the MR‐inferred relationship between immune cells and fractures was significant, with the mediation proportion serving as an effect indicator. The estimated impact of potential mediators on fracture risk is detailed in Supporting Information 2: Table S48.

Several metabolites were identified, including 5‐methylthioadenosine (MTA), linolenoylcarnitine (C18:3), the salicylate to oxalate ratio, the taurine to cysteine ratio, and several unannotated metabolites (X‐12100, X‐13723, X‐15486, X‐22776, and X‐23782), all exhibiting pleiotropic properties and showing associations with multiple immune cells in relation to fractures.

For foot fractures (excluding the ankle), the taurine to cysteine ratio and X‐23782 acted as mediators. In forearm fractures, the cytidine to *N*‐acetylneuraminate ratio mediated the relationship, with an 18.3% mediation proportion. For lumbar spine/pelvic fractures, X‐22776 was a key mediator with a 29.2% mediation proportion. The salicylate to oxalate (ethanedioate) ratio served as a significant mediator for neck fractures, accounting for a 27% mediation proportion. For wrist fractures, the most abundant metabolite suggested a mediator effect exceeding 15%. These findings highlight the complex interactions between the immune system, metabolic factors, and fracture susceptibility, suggesting that different metabolites may play varying roles in mediating the pathways linking immune cells and fractures at distinct skeletal sites.

### 3.4. Identification of Relevant Pathways and Targets of Immune Cells and Metabolites Associated With Fractures

Following the bidirectional MR and two‐sample MR analysis, SNPs associated with immune cells and metabolites were identified as causally linked to fractures. After removing duplicates, 486 SNPs related to immune cells and fractures were pinpointed (Supporting Information 2: Table S49), along with 1014 SNPs linked to metabolites and fractures (Supporting Information 2: Table S50), and 1018 SNPs connecting immune cells to metabolites (Supporting Information 2: Table S51). Using the vautils package, 615 genes (β2: SNP nearest gene) associated with immune cells and fractures were identified, along with 1300 genes (β3: SNP nearest gene) linked to metabolites and fractures, and 1185 genes (β1: SNP nearest gene) correlated with both immune cells and metabolites (Supporting Information 2: Table S52).

To explore mechanistic connections, KEGG and GO enrichment analyses were performed on SNP‐associated genes to investigate potential links between immune cells, metabolites, and fractures. The SNP nearest genes of β1 showed significant enrichment in KEGG pathways related to cell adhesion, immune responses, and autoimmune diseases (Figure 4A). GO analyses revealed associations with plasma membrane structures, MHC protein complexes, and cell adhesion processes (Figure 4B).

Figure 4Top 10 KEGG Pathway and GO Enrichment Analysis of SNP‐Associated Genes for MR of Causality Between Immune Cells/Metabolites and Fractures. (A) KEGG enrichment analysis of SNP‐associated genes for MR of causality between immune cells and metabolites. (B) GO enrichment analysis of SNP‐associated genes for MR of causality between immune cells and metabolites. (C) KEGG enrichment analysis of SNP‐associated genes for MR of causality between immune cells and various fracture sites. (D) GO enrichment analysis of SNP‐associated genes for MR of causality between immune cells and various fracture sites. (E) KEGG enrichment analysis of SNP‐associated genes for MR of causality between metabolites and various fracture sites. (F) GO enrichment analysis of SNP‐associated genes for MR of causality between metabolites and various fracture sites. The β value represents the effect size of the SNP in two‐sample MR. β1 represents the effect value of the SNP for immune cells and metabolites, β2 represents the effect value of the SNP for immune cells and fractures, and β3 represents the effect value of the SNP for metabolites and fractures. All pathways with *p*  < 0.05 were considered statistically significant.

**Figure:**
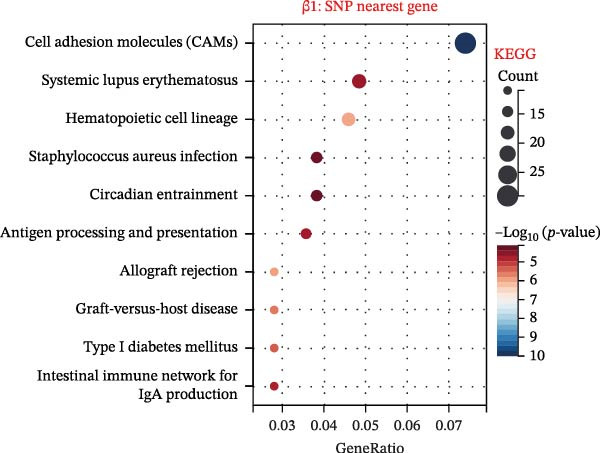
(A)

**Figure:**
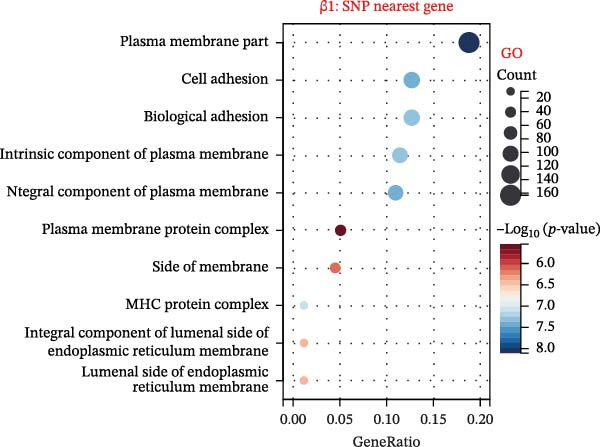
(B)

**Figure:**
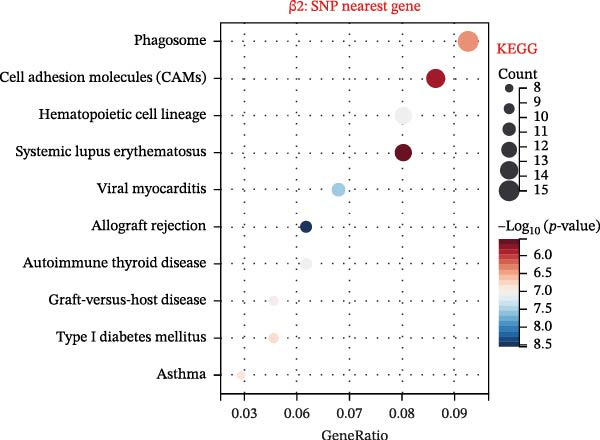
(C)

**Figure:**
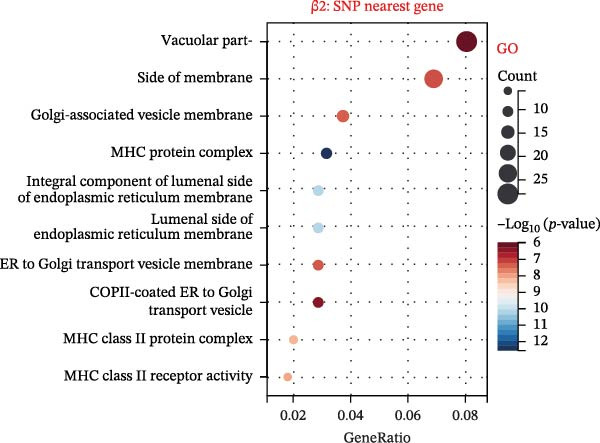
(D)

**Figure:**
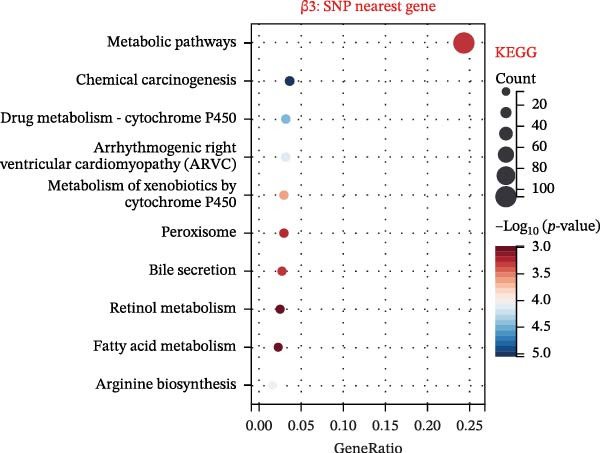
(E)

**Figure:**
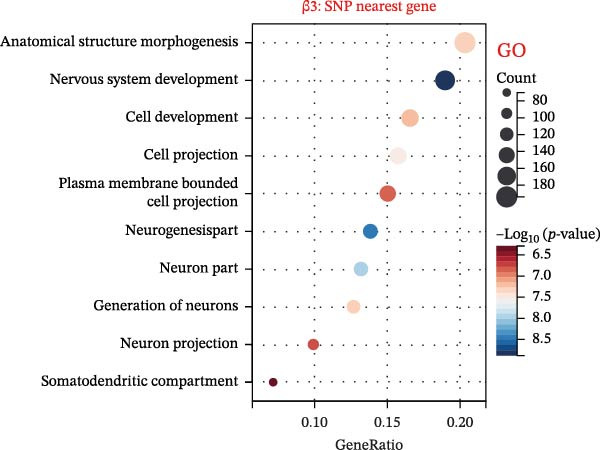
(F)

For β2 SNP nearest genes, KEGG pathway analyses suggested enrichment in immune response, autoimmune diseases, and cellular adhesion pathways (Figure 4C). GO analyses indicated roles in MHC protein complexes, Golgi‐associated vesicle membranes, and receptor activities (Figure 4D). For β3 SNP nearest genes, KEGG analysis highlighted enrichment in metabolic and biosynthetic pathways, drug metabolism, and cardiomyopathy (Figure 4E). GO analyses revealed associations with neural development, cell projection processes, and anatomical structure morphogenesis (all *p*  < 0.05, Figure 4F). All relevant gene set enrichment pathways (FDR < 0.05) are summarized in Supporting Information 2: Tables S53−S58.

Common GO pathways across β1, β2, and β3 indicated intersections involving the endomembrane system, plasma membrane integrity, and membrane protein complexes (Figure 5A,B, Supporting Information 1: Figure S20). However, KEGG enrichment analysis did not reveal intersecting pathways across the three groups (Supporting Information 1: Figure S21). A total of 789 genes corresponding to common GO pathways were merged. PPI analysis by degree identified 195 genes interacting at a 0.9 confidence threshold. The top 20 genes were evaluated using four algorithms: degree, EPC, bottleneck, and closeness (Figure 5C). Convergence from these methods highlighted 9 key genes: TP53, ITPR1, STAT3, IL‐6, ITGB1, NOS1, CD8A, TNF, and PRKACA (Supporting Information 1: Figure S22). In summary, this integrative analysis offers preliminary insights into the potential biological links between immune cells, metabolites, and fracture risk, identifying specific pathways and molecular patterns that merit further investigation.

Figure 5Multisample Mendelian causality for gene set enrichment common GO enrichment pathways and PPI analysis of GO pathway‐related genes. (A) Intersection of SNP‐associated gene enrichment pathways obtained by three MR analyses. (B) Bubble diagram illustrating gene enrichment pathways for five common GO pathways in MR analysis of causal SNPs between immune cells and fractures. (C) PPI analysis of five GO pathway‐related genes, visualized within a network at a confidence interval of 0.9. Network centrality analysis using four methods (degree, EPC, bottleneck, and closeness) to identify the top 20 relevant GO pathway‐related genes, with each method highlighting central nodes in the network. β1 represents the effect value of the SNP for immune cells and metabolites, β2 represents the effect value of the SNP for immune cells and fractures, and β3 represents the effect value of the SNP for metabolites and fractures. All pathways with *p*  < 0.05 were considered statistically significant.

**Figure:**
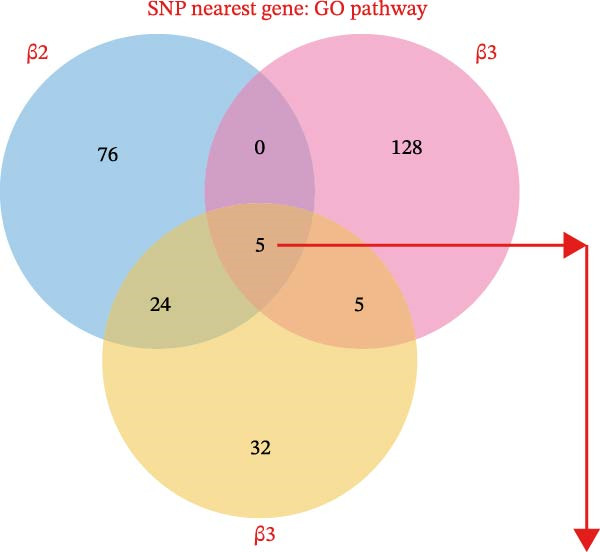
(A)

**Figure:**
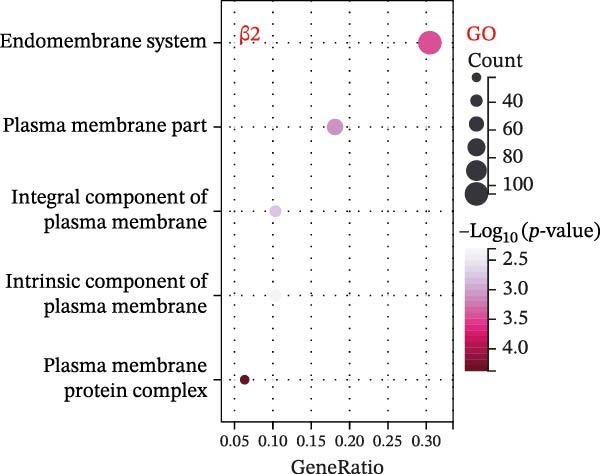
(B)

**Figure:**
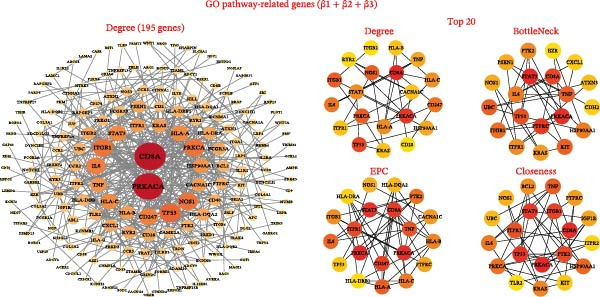
(C)

### 3.5. Colocalization Between Hub Genes and Fractures

Close LD can lead to inaccurate MR results due to horizontal pleiotropy [50]. Colocalization analysis is a valuable tool for identifying shared causal SNPs that are associated with both the exposure and the outcome [47]. Six key genes—CD8A, PRKACA, IL‐6, ITGB1, ITPR1, and STAT3—were found to colocalize with 36 fracture data points, with specific details provided in Supporting Information 2:Tables S59 and S60. Colocalization results are summarized in Supporting Information 2: Table S61 (PH4 > 0.75). Among hub genes and fracture‐related GWAS, nine colocalized SNPs were identified, including rs7596329, rs147796716, rs35116860, rs2905333, rs11009173, rs1187095, rs2819588, rs13325272, and rs1053004 (Figure 6). These findings provide genetic evidence that contributes to our understanding of fracture‐related biological pathways and may inform future mechanistic research toward potential preventive strategies.

Figure 6Locus comparison plots for the shared causal variant in the associations of six key genes (CD8A, PRKACA, IL‐6, ITGB1, ITPR1, and STAT3) with fractures. Colocalization analysis revealed a shared causal variant within the gene region for each gene and fracture. This variant served as the lead cis‐eQTL and was strongly correlated with the lead fracture GWAS variant, with a high posterior probability (PPH4 > 0.75), providing preliminary evidence for a shared genetic basis. Labels for SNP: (A) rs2905333, (B) rs35116860, (C) rs1187095, (D) rs11009173, (E) rs2819588, (F) rs13325272, (G) rs7596329, (H) rs147796716, and (I) rs1053004. The *r* ^2^ value measures the linkage disequilibrium between each variant and the lead single nucleotide polymorphism, reflecting the strength of their genetic association. All the presented results are statistically significant, with a *p*‐value of less than 0.05.

**Figure:**
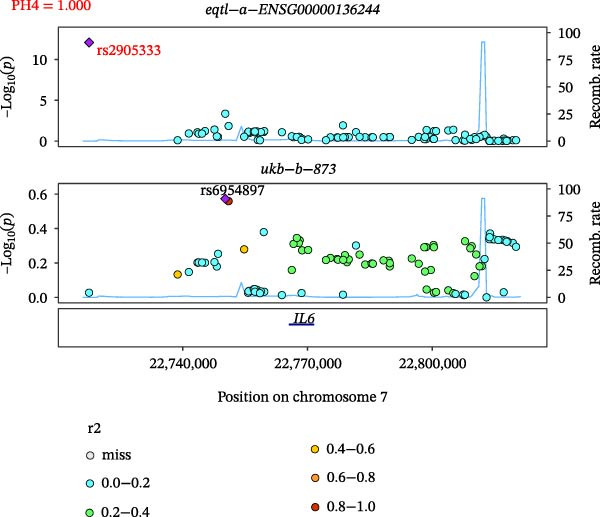
(A)

**Figure:**
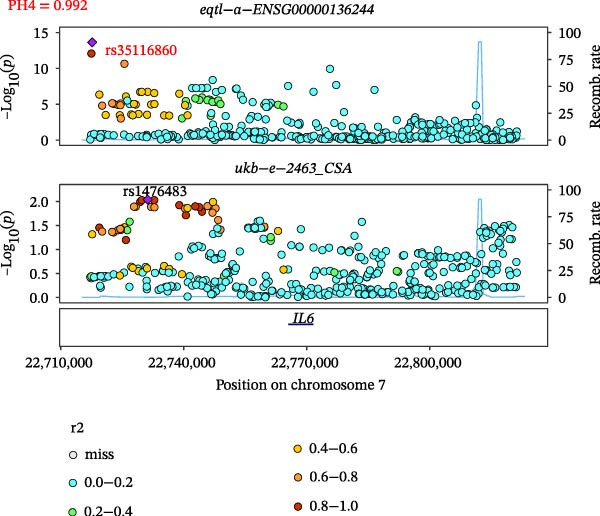
(B)

**Figure:**
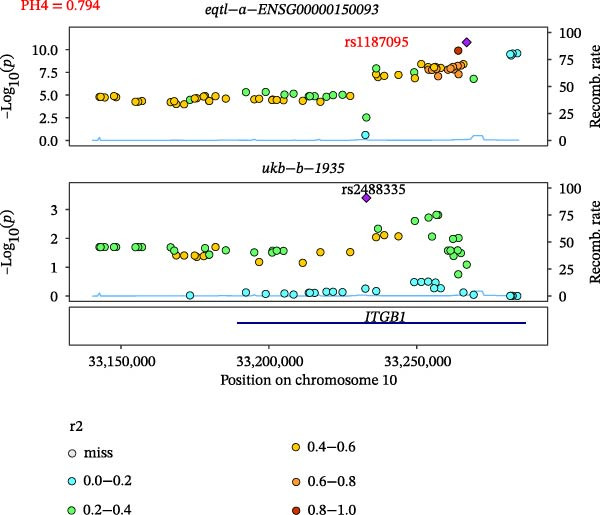
(C)

**Figure:**
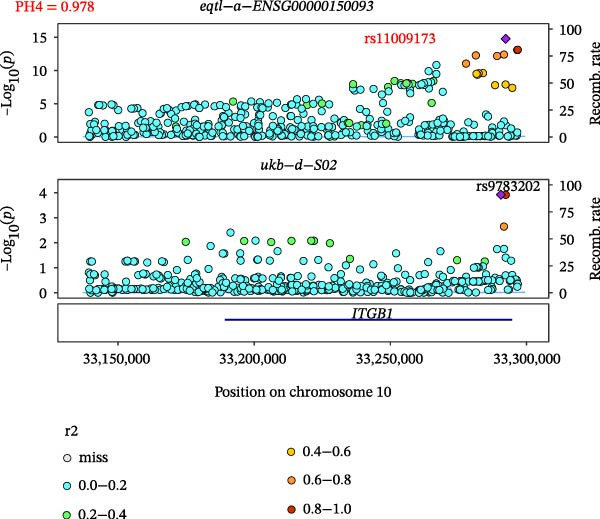
(D)

**Figure:**
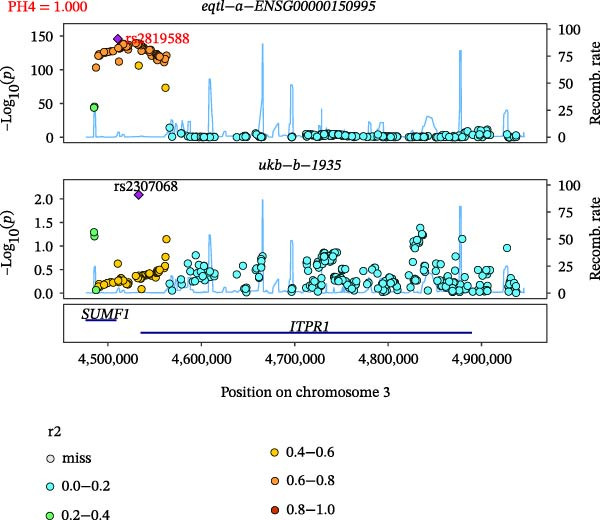
(E)

**Figure:**
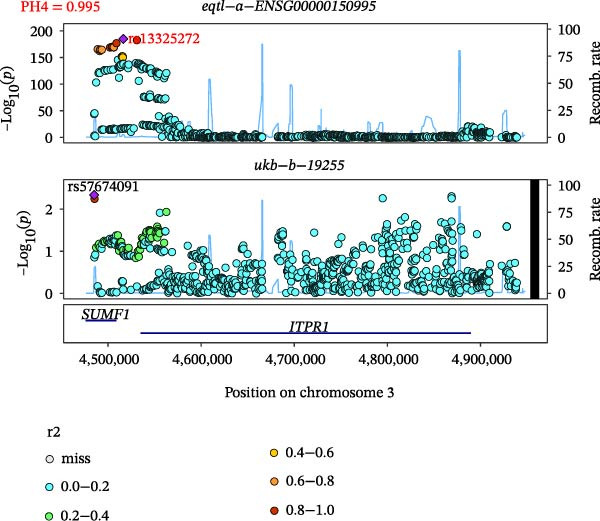
(F)

**Figure:**
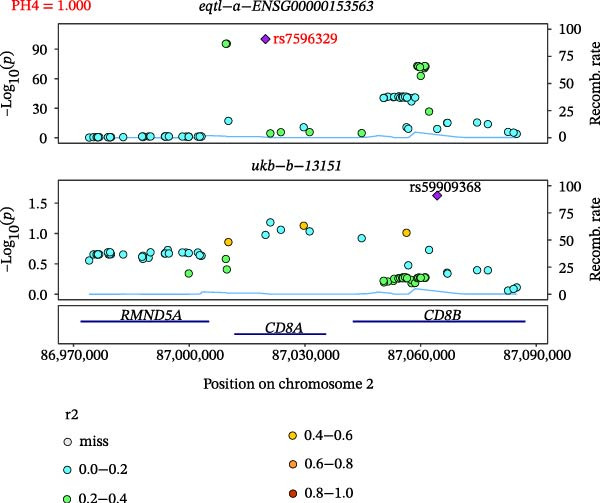
(G)

**Figure:**
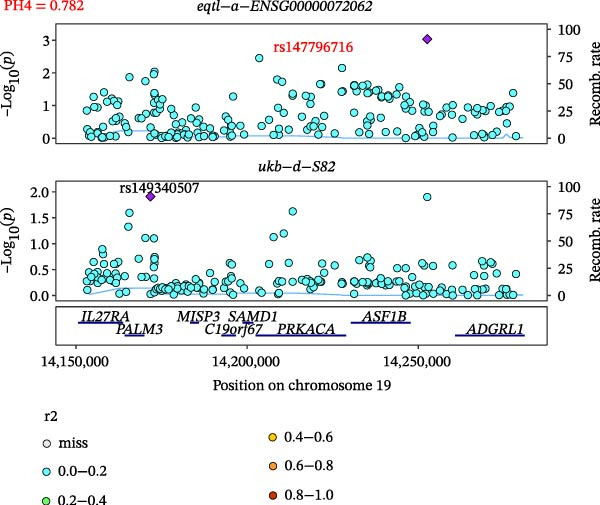
(H)

**Figure:**
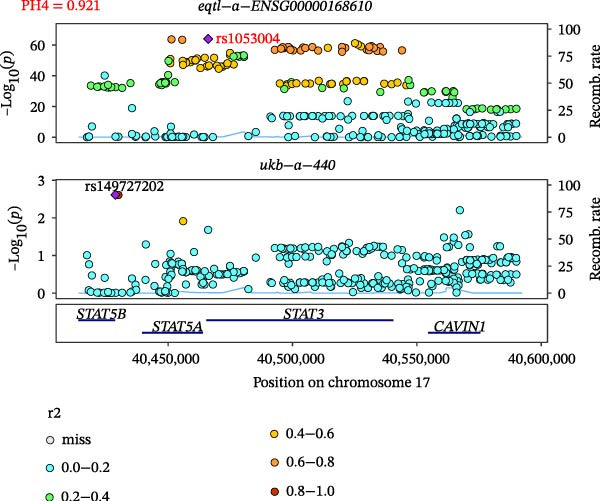
(I)

### 3.6. Two‐Sample MR Analysis of DNA Methylation and Fractures

To further explore connections between CpG sites in hub genes and the nine fracture types, the two‐sample MR method was employed. The advent of Epigenome‐Wide Association Studies (EWAS) has provided a robust framework for investigating complex traits. Methylation sites for key genes were identified using the EWAS Data Hub (https://ngdc.cncb.ac.cn/ewas/datahub/) (Supporting Information 2: Table S62) [51]. MR analyses were conducted using the IVW method to estimate causal relationships between these CpG sites and specific fracture outcomes (Supporting Information 1: Figures S23–S31). Additional MR analyses using four complementary methods assessed the joint relationships between fractures and hub gene CpG loci (Supporting Information 2: Tables S63−S71).

**Figure 7:**
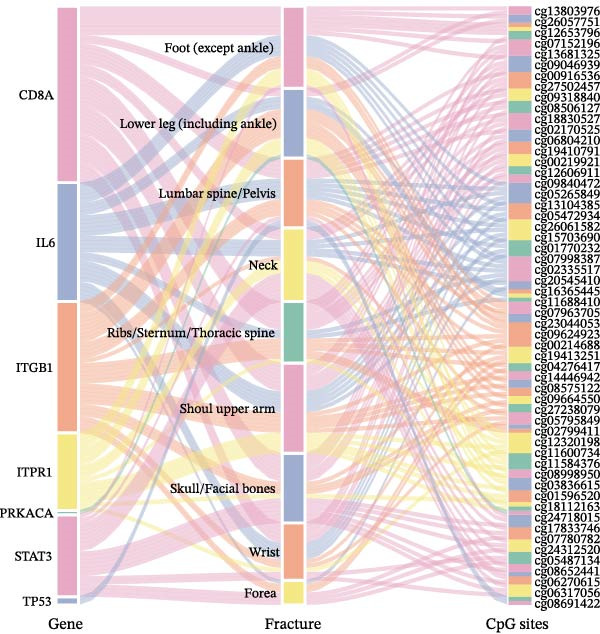
Summary of MR relationships between fractures and CpG sites of hub genes. All the presented results are statistically significant, with a *p*‐value of less than 0.05.

Distinct roles and associations between various gene methylation sites and MR causal relationships emerged across different fracture sites. A Sankey diagram summarizes these MR‐inferred relationships between fractures and CpG sites of hub genes (Figure 7). Notably, the CD8A methylation site exhibited MR associations, indicating detrimental effects for foot and lower leg fractures, while showing protective associations for lumbar spine/pelvis, neck, ribs/sternum/thoracic spine, shoulder/upper arm, and skull/facial bone fractures. The methylation sites of CD8A displayed opposite MR causal associations in wrist fractures, with no MR causal links identified for forearm fractures (all *p*  < 0.05) (Supporting Information 1: Figures S23–S31). Methylation site cg09664550 of ITGB1 showed the most significant negative impact on ribs/sternum/thoracic spine fractures (OR = 1.986), while cg18112163 of STAT3 exhibited strong protective effects on foot fractures (excluding the ankle) (OR = 0.602, all *p*  < 0.05) (Supporting Information 1: Figures S23, S28). Sensitivity analyses confirmed the robustness of the findings for the majority of significant methylation site‐fracture pairs. Evidence of horizontal pleiotropy was absent in 99.3% (145 out of 146) of associations (MR‐Egger intercept *p*  > 0.05), and 97.3% (142 out of 146) showed no significant heterogeneity (Cochran’s Q *p*  > 0.05). This high consistency reinforces the reliability of the inferred causal effects between DNA methylation and site‐specific fracture risk (Supporting Information 1: Figures S23–S31). These findings delineate the site‐specific epigenetic complexity underlying fracture risk. They suggest potential pathways for future exploration but do not yet constitute a basis for clinical application.

## 4. Discussion

Fractures pose a significant clinical challenge, highlighting the need for standardized international guidelines for prevention and management [3, 52]. A crucial step in improving outcomes is the identification of shared biological pathways and therapeutic targets that can mitigate fracture progression and enhance healing. While MR studies have expanded our understanding of fracture causality—linking fractures to diseases [8], immune cells [26], metabolites [53], and inflammatory factors [15]—current research remains limited by a narrow focus on specific fracture sites, exposure factors, and imprecise subtype classifications. Furthermore, conventional Mendelian associations provide insufficient mechanistic insights into fracture injury and healing, hindering the development of targeted therapies. To address these gaps, this study systematically explored MR‐based associations to identify potential causal relationships across 731 immune cell traits and 1400 metabolites in relation to nine fracture sites, uncovering intrinsic mechanistic connections. The analysis of SNP nearest genes linked to fracture‐associated genetic variants revealed key biological pathways and potential therapeutic targets, suggesting new directions for future research in fracture pathophysiology.

The findings reveal the complex interactions between immune regulation, metabolic processes, and fracture susceptibility. Notably, SNP nearest genes (β1 and β2) were enriched in pathways related to immune responses, autoimmune disorders, and cell adhesion, indicating a potential direct influence of immune function on fracture risk. Mechanistically, this can be understood through osteoimmunology, where immune cell subtypes such as T helper 17 (Th17) cells promote osteoclastogenesis via RANKL [54], while regulatory T cells (Tregs) can exert protective effects through cytokines like TGF‐β and IL‐10 [55]. These genetic associations also implicated MHC protein complexes, further emphasizing the role of immune cells in bone homeostasis. Conversely, metabolic and biosynthetic pathways (β3)—along with connections to drug metabolism and cardiomyopathy—highlight metabolites as critical regulators of bone metabolism and fracture risk. For instance, dysregulated lipid metabolism can drive bone marrow adiposity at the expense of osteoblast formation [56], while defects in mitochondrial metabolites may impair the bioenergetics of bone remodeling [57]. Additionally, neural development and cellular processes associated with these SNPs suggest broader systemic interactions that influence fracture outcomes. Collectively, these results suggest that fractures involve more than just structural damage; they may also reflect underlying immune‐metabolic dysregulation [58], potentially explaining the frequent co‐occurrence of immune disorders, metabolic diseases, and fractures [59].

Further pathway analysis highlighted the role of the endomembrane system, plasma membrane integrity, and membrane‐associated protein complexes in fracture biology. The endomembrane system (including the endoplasmic reticulum and Golgi apparatus) is critical for protein trafficking and signaling, which are essential for bone remodeling [60, 61]. It also serves as a key platform for lipid synthesis and calcium storage, processes fundamental to osteoblast and osteoclast function [62]. Plasma membrane proteins mediate cell–cell interactions vital for osteoblast and osteoclast activity [63]. Crucially, it is at the plasma membrane that receptors like RANK and Ephrins form complexes to direct bone cell fate and crosstalk [64]. Additionally, membrane‐localized protein complexes facilitate signal transduction and immune‐skeletal crosstalk during fracture repair [65], suggesting that immune and metabolic factors may influence bone healing through membrane‐dependent mechanisms. These insights could guide the development of precision therapeutics targeting these pathways.

Colocalization analysis strengthened the evidence for shared genetic mechanisms by identifying common SNPs between hub genes (CD8A, PRKACA, IL‐6, ITGB1, ITPR1, and STAT3) and fracture risk. Notably, IL‐6 is a well‐established biomarker for osteoporotic fractures [66], and STAT3 modulation has been shown to either alleviate or exacerbate bone loss in preclinical models [67]. These findings highlight the translational relevance of these genes in fracture pathophysiology.

Epigenetic investigations identified DNA methylation as a potential critical regulatory mechanism, with ITGB1 (cg09664550) significantly associated with rib, sternum, and thoracic fractures, while STAT3 (cg18112163) exhibited a protective effect against foot fractures. Given that ITGB1‐mediated pathways contribute to cartilage regeneration [68], these methylation sites may serve as potential biomarkers or therapeutic targets for personalized fracture prevention.

Several limitations should be considered. First, the preliminary analyses without multiple testing correction revealed widespread associations. However, after FDR adjustment, only three immune cells and eight metabolites remained significantly linked to individual fracture types, underscoring the exploratory nature of these findings. Second, all results are based on secondary genetic data and lack independent cohort validation, necessitating future replication. Third, the predominantly European ancestry of the GWAS data limits the generalizability across populations. Fourth, colocalization focused on only six hub genes, potentially excluding other causal loci. Fifth, the research implications of the identified unannotated metabolites (e.g., X‐12100) and specific CpG sites remain speculative and require extensive functional validation to elucidate their biological roles and translational potential. Lastly, the limited sample sizes of the source GWAS for immune cells and metabolites necessitated the use of a relaxed IV selection threshold (*p*  < 1 × 10^−5^), which might have included weak variants despite sensitivity analyses and *F*‐statistic checks. As such, these results represent robust genetic evidence but require validation in multiethnic cohorts, confirmatory statistical analyses, and orthogonal experimental studies to establish biological mechanisms and clinical relevance.

By integrating multiomic MR analyses, this study illuminates the immune‐metabolic‐skeletal axis in fracture susceptibility and healing. The identification of key methylation sites and hub genes offers promising epigenetic and molecular candidates for personalized fracture prevention and treatment strategies. These findings provide preliminary genetic evidence and mechanistic insights, which can inform future investigations into fracture pathophysiology.

**Nomenclature**
AC: Absolute cell counts
BMD: Bone mineral density
CI: Confidence interval
EPC: Edge percolated component
GO: Gene ontology
GWAS: Genome‐wide association studies
IVS: Instrumental variable selection
KEGG: Kyoto Encyclopedia of Genes and Genomes
MFI: Median fluorescence intensities
MP: Morphological parameters
mQTL: Methylation quantitative trait loci
MR: Mendelian Randomization
OR: Odds ratio
PPI: Protein–protein interaction
RC: Relative counts
TBNK cells: T cells, B cells, and natural killer cells.

## Author Contributions

Xiaomin Wan and Wuchao Lu conceived and designed the study. Jizhao Xue and Jiaxin Huang collected the data and managed resources. Xiaomin Wan, Weihong Qian, and Zhuoyi Hu performed statistical analyses, including software implementation, validation, and formal data analysis. Xiaomin Wan and Weihong Qian created visualizations. Xiaomin Wan wrote the original draft. Wulin You and Yafeng Zhang contributed to methodology development and manuscript revision. Wuchao Lu supervised the project, administered its execution and acquired funding.

## Funding

This work was supported by the Top Talent Support Program for Young and Middle‐aged People of Wuxi Health Committee, Jiangsu, China (Grant BJ2023070) and Wuxi Taihu Lake Talent Plan, Supports for Leading Talents in Medical and Health Profession, Jiangsu, China (Grant 202001).

## Disclosure

All authors reviewed and approved the final manuscript. No persons or third‐party services outside the listed authors were involved in this work.

## Ethics Statement

The authors have nothing to report.

## Consent

The authors have nothing to report.

## Conflicts of Interest

The authors declare no conflicts of interest.

## Supporting Information

Additional supporting information can be found online in the Supporting Information section.

## Supporting information


**Supporting Information 1** Figure S1: Assessing MR causality between immune cells and fractures (foot, excluding ankle). Exposure comprises 731 immune cell phenotypes, while the outcome is defined as fractures (foot, excluding ankle); nSNP: number of single nucleotide polymorphisms; method: inverse variance weighting; OR: odds ratio; CI: confidence interval. The odds ratio (OR) and confidence interval (CI) are calculated, with OR > 1 indicating that the exposure is a risk factor for the outcome and OR < 1 suggesting it serves as a protective factor. Heterogeneity is analyzed using Q, with Q_df representing the degrees of freedom; a Q_pval <0.05 indicates significant heterogeneity. The Egger_intercept is used for pleiotropy analysis, with E_se denoting the standard error. A *p*‐value (E_pval) <0.05 signifies the presence of pleiotropy. Figure S2: Assessing MR causality between immune cells and fractures (forea). Exposure comprises 731 immune cell phenotypes, while the outcome is defined as fractures (forea); nSNP: number of single nucleotide polymorphisms; method: inverse variance weighting; OR: odds ratio; CI: confidence interval. The odds ratio (OR) and confidence interval (CI) are calculated, with OR > 1 indicating that the exposure is a risk factor for the outcome and OR < 1 suggesting it serves as a protective factor. Heterogeneity is analyzed using Q, with Q_df representing the degrees of freedom; a Q_pval <0.05 indicates significant heterogeneity. The Egger_intercept is used for pleiotropy analysis, with E_se denoting the standard error. A *p*‐value (E_pval) <0.05 signifies the presence of pleiotropy. Figure S3: Assessing MR causality between immune cells and fractures (lower leg, including ankle). Exposure comprises 731 immune cell phenotypes, while the outcome is defined as fractures (lower leg, including ankle); nSNP: number of single nucleotide polymorphisms; method: inverse variance weighting; OR: odds ratio; CI: confidence interval. The odds ratio (OR) and confidence interval (CI) are calculated, with OR > 1 indicating that the exposure is a risk factor for the outcome and OR < 1 suggesting it serves as a protective factor. Heterogeneity is analyzed using Q, with Q_df representing the degrees of freedom; a Q_pval <0.05 indicates significant heterogeneity. The Egger_intercept is used for pleiotropy analysis, with E_se denoting the standard error. A *p*‐value (E_pval) <0.05 signifies the presence of pleiotropy. Figure S4: Assessing MR causality between immune cells and fractures (lumbar spine/pelvis). Exposure comprises 731 immune cell phenotypes, while the outcome is defined as fractures (lumber spine/pelvis); nSNP: number of single nucleotide polymorphisms; method: inverse variance weighting; OR: odds ratio; CI: confidence interval. The odds ratio (OR) and confidence interval (CI) are calculated, with OR > 1 indicating that the exposure is a risk factor for the outcome and OR < 1 suggesting it serves as a protective factor. Heterogeneity is analyzed using Q, with Q_df representing the degrees of freedom; a Q_pval <0.05 indicates significant heterogeneity. The Egger_intercept is used for pleiotropy analysis, with E_se denoting the standard error. A *p*‐value (E_pval) <0.05 signifies the presence of pleiotropy. Figure S5: Assessing MR causality between immune cells and fractures (neck). Exposure comprises 731 immune cell phenotypes, while the outcome is defined as fractures (neck); nSNP: number of single nucleotide polymorphisms; method: inverse variance weighting; OR: odds ratio; CI: confidence interval. The odds ratio (OR) and confidence interval (CI) are calculated, with OR > 1 indicating that the exposure is a risk factor for the outcome and OR < 1suggesting it serves as a protective factor. Heterogeneity is analyzed using Q, with Q_df representing the degrees of freedom; a Q_pval <0.05 indicates significant heterogeneity. The Egger_intercept is used for pleiotropy analysis, with E_se denoting the standard error. A *p*‐value (E_pval) <0.05 signifies the presence of pleiotropy. Figure S6: Assessing MR causality between immune cells and fractures (ribs/sternum/thoracic spine). Exposure comprises 731 immune cell phenotypes, while the outcome is defined as fractures (ribs/sternum/thoracic spine); nSNP: number of single nucleotide polymorphisms; method: inverse variance weighting; OR: odds ratio; CI: confidence interval. The odds ratio (OR) and confidence interval (CI) are calculated, with OR > 1 indicating that the exposure is a risk factor for the outcome and OR < 1 suggesting it serves as a protective factor. Heterogeneity is analyzed using Q, with Q_df representing the degrees of freedom; a Q_pval <0.05 indicates significant heterogeneity. The Egger_intercept is used for pleiotropy analysis, with E_se denoting the standard error. A *p*‐value (E_pval) <0.05 signifies the presence of pleiotropy. Figure S7: Assessing MR causality between immune cells and fractures (shouler/upper arm). Exposure comprises 731 immune cell phenotypes, while the outcome is defined as fractures (shoulder/upper arm); nSNP: number of single nucleotide polymorphisms; method: inverse variance weighting; OR: odds ratio; CI: confidence interval. The odds ratio (OR) and confidence interval (CI) are calculated, with OR > 1 indicating that the exposure is a risk factor for the outcome and OR < 1 suggesting it serves as a protective factor. Heterogeneity is analyzed using Q, with Q_df representing the degrees of freedom; a Q_pval <0.05 indicates significant heterogeneity. The Egger_intercept is used for pleiotropy analysis, with E_se denoting the standard error. A *p*‐value (E_pval) <0.05 signifies the presence of pleiotropy. Figure S8: Assessing MR causality between immune cells and fractures (skull/facial bones). Exposure comprises 731 immune cell phenotypes, while the outcome is defined as fractures (skull/facial bones); nSNP: number of single nucleotide polymorphisms; method: inverse variance weighting; OR: odds ratio; CI: confidence interval. The odds ratio (OR) and confidence interval (CI) are calculated, with OR > 1 indicating that the exposure is a risk factor for the outcome and OR < 1 suggesting it serves as a protective factor. Heterogeneity is analyzed using Q, with Q_df representing the degrees of freedom; a Q_pval <0.05 indicates significant heterogeneity. The Egger_intercept is used for pleiotropy analysis, with E_se denoting the standard error. A *p*‐value (E_pval) <0.05 signifies the presence of pleiotropy. Figure S9: Assessing MR causality between immune cells and fractures (wrist). Exposure comprises 731 immune cell phenotypes, while the outcome is defined as fractures (wrist); nSNP: number of single nucleotide polymorphisms; method: inverse variance weighting; OR: odds ratio; CI: confidence interval. The odds ratio (OR) and confidence interval (CI) are calculated, with OR > 1 indicating that the exposure is a risk factor for the outcome and OR < 1 suggesting it serves as a protective factor. Heterogeneity is analyzed using Q, with Q_df representing the degrees of freedom; a Q_pval <0.05 indicates significant heterogeneity. The Egger_intercept is used for pleiotropy analysis, with E_se denoting the standard error. A *p*‐value (E_pval) <0.05 signifies the presence of pleiotropy. Figure S10: Assessing MR causality between metabolites and fractures (foot, excluding ankle). Exposure comprises 1400 metabolites, while the outcome is defined as fractures (foot, excluding ankle); nSNP: number of single nucleotide polymorphisms; method: inverse variance weighting; OR: odds ratio; CI: confidence interval. The odds ratio (OR) and confidence interval (CI) are calculated, with OR > 1 indicating that the exposure is a risk factor for the outcome and OR < 1 suggesting it serves as a protective factor. Heterogeneity is analyzed using Q, with Q_df representing the degrees of freedom; a Q_pval <0.05 indicates significant heterogeneity. The Egger_intercept is used for pleiotropy analysis, with E_se denoting the standard error. A *p*‐value (E_pval) <0.05 signifies the presence of pleiotropy. Figure S11: Assessing MR causality between metabolites and fractures (forea). Exposure comprises 1400 metabolites, while the outcome is defined as fractures (forea); nSNP: number of single nucleotide polymorphisms; method: inverse variance weighting; OR: odds ratio; CI: confidence interval. The odds ratio (OR) and confidence interval (CI) are calculated, with OR > 1 indicating that the exposure is a risk factor for the outcome and OR < 1 suggesting it serves as a protective factor. Heterogeneity is analyzed using Q, with Q_df representing the degrees of freedom; a Q_pval <0.05 indicates significant heterogeneity. The Egger_intercept is used for pleiotropy analysis, with E_se denoting the standard error. A *p*‐value (E_pval) <0.05 signifies the presence of pleiotropy. Figure S12: Assessing MR causality between metabolites and fractures (lower leg, including ankle). Exposure comprises 1400 metabolites, while the outcome is defined as fractures (lower leg, including ankle); nSNP: number of single nucleotide polymorphisms; method: inverse variance weighting; OR: odds ratio; CI: confidence interval. The odds ratio (OR) and confidence interval (CI) are calculated, with OR > 1 indicating that the exposure is a risk factor for the outcome and OR < 1 suggesting it serves as a protective factor. Heterogeneity is analyzed using Q, with Q_df representing the degrees of freedom; a Q_pval <0.05 indicates significant heterogeneity. The Egger_intercept is used for pleiotropy analysis, with E_se denoting the standard error. A *p*‐value (E_pval) <0.05 signifies the presence of pleiotropy. Figure S13: Assessing MR causality between metabolites and fractures (lumbar spine/pelvis). Exposure comprises 1400 metabolites, while the outcome is defined as fractures (lumbar spine/pelvis); nSNP: number of single nucleotide polymorphisms; method: inverse variance weighting; OR: odds ratio; CI: confidence interval. The odds ratio (OR) and confidence interval (CI) are calculated, with OR > 1 indicating that the exposure is a risk factor for the outcome and OR < 1 suggesting it serves as a protective factor. Heterogeneity is analyzed using Q, with Q_df representing the degrees of freedom; a Q_pval <0.05 indicates significant heterogeneity. The Egger_intercept is used for pleiotropy analysis, with E_se denoting the standard error. A *p*‐value (E_pval) <0.05 signifies the presence of pleiotropy. Figure S14: Assessing MR causality between metabolites and fractures (neck). Exposure comprises 1400 metabolites, while the outcome is defined as fractures (neck); nSNP: number of single nucleotide polymorphisms; method: inverse variance weighting; OR: odds ratio; CI: confidence interval. The odds ratio (OR) and confidence interval (CI) are calculated, with OR > 1 indicating that the exposure is a risk factor for the outcome and OR < 1 suggesting it serves as a protective factor. Heterogeneity is analyzed using Q, with Q_df representing the degrees of freedom; a Q_pval <0.05 indicates significant heterogeneity. The Egger_intercept is used for pleiotropy analysis, with E_se denoting the standard error. A *p*‐value (E_pval) <0.05 signifies the presence of pleiotropy. Figure S15: Assessing MR causality between metabolites and fractures (ribs/sternum/thoracic spine). Exposure comprises 1400 metabolites, while the outcome is defined as fractures (ribs/sternum/thoracic spine); nSNP: number of single nucleotide polymorphisms; method: inverse variance weighting; OR: odds ratio; CI: confidence interval. The odds ratio (OR) and confidence interval (CI) are calculated, with OR > 1 indicating that the exposure is a risk factor for the outcome and OR < 1 suggesting it serves as a protective factor. Heterogeneity is analyzed using Q, with Q_df representing the degrees of freedom; a Q_pval <0.05 indicates significant heterogeneity. The Egger_intercept is used for pleiotropy analysis, with E_se denoting the standard error. A *p*‐value (E_pval) <0.05 signifies the presence of pleiotropy. Figure S16: Assessing MR causality between metabolites and fractures (shoulder/upper arm). Exposure comprises 1400 metabolites, while the outcome is defined as fractures (shoulder/upper arm); nSNP: number of single nucleotide polymorphisms; method: inverse variance weighting; OR: odds ratio; CI: confidence interval. The odds ratio (OR) and confidence interval (CI) are calculated, with OR > 1 indicating that the exposure is a risk factor for the outcome and OR < 1 suggesting it serves as a protective factor. Heterogeneity is analyzed using Q, with Q_df representing the degrees of freedom; a Q_pval <0.05 indicates significant heterogeneity. The Egger_intercept is used for pleiotropy analysis, with E_se denoting the standard error. A *p*‐value (E_pval) <0.05 signifies the presence of pleiotropy. Figure S17: Assessing MR causality between metabolites and fracture (skull/facial bones). Exposure comprises 1400 metabolites, while the outcome is defined as fracture (skull/facial bones); nSNP: number of single nucleotide polymorphisms; method: inverse variance weighting; OR: odds ratio; CI: confidence interval. The odds ratio (OR) and confidence interval (CI) are calculated, with OR > 1 indicating that the exposure is a risk factor for the outcome and OR < 1 suggesting it serves as a protective factor. Heterogeneity is analyzed using Q, with Q_df representing the degrees of freedom; a Q_pval <0.05 indicates significant heterogeneity. The Egger_intercept is used for pleiotropy analysis, with E_se denoting the standard error. A *p*‐value (E_pval) <0.05 signifies the presence of pleiotropy. Figure S17: Assessing MR causality between metabolites and fractures (skull/facial bones). Exposure comprises 1400 metabolites, while the outcome is defined as fractures (skull/facial bones); nSNP: number of single nucleotide polymorphisms; method: inverse variance weighting; OR: odds ratio; CI: confidence interval. The odds ratio (OR) and confidence interval (CI) are calculated, with OR > 1 indicating that the exposure is a risk factor for the outcome and OR < 1 suggesting it serves as a protective factor. Heterogeneity is analyzed using Q, with Q_df representing the degrees of freedom; a Q_pval <0.05 indicates significant heterogeneity. The Egger_intercept is used for pleiotropy analysis, with E_se denoting the standard error. A *p*‐value (E_pval) <0.05 signifies the presence of pleiotropy. Figure S18: Assessing MR causality between metabolites and fractures (wrist). Exposure comprises 1400 metabolites, while the outcome is defined as fractures (wrist); nSNP: number of single nucleotide polymorphisms; method: inverse variance weighting; OR: odds ratio; CI: confidence interval. The odds ratio (OR) and confidence interval (CI) are calculated, with OR > 1 indicating that the exposure is a risk factor for the outcome and OR < 1 suggesting it serves as a protective factor. Heterogeneity is analyzed using Q, with Q_df representing the degrees of freedom; a Q_pval <0.05 indicates significant heterogeneity. The Egger_intercept is used for pleiotropy analysis, with E_se denoting the standard error. A *p*‐value (E_pval) <0.05 signifies the presence of pleiotropy. Figure S19: Statistical results of leave‐one‐out (LOO) sensitivity analysis on immune cells and metabolites associated with fractures by MR analysis. Figures A–C exemplify two scenarios in the current LOO sensitivity analysis: (A) Maximum Criterion: the LOO CI for all SNPs do not cross or include zero (the black vertical dashed line) and remain consistent (all on the same side). (B, C) Minimum Criterion: the LOO CI for most SNPs do not cross or include zero (the black vertical dashed line) and are consistent (all on the same side). The proportion of Minimum Criterion and Maximum Criterion in the LOO sensitivity analysis results was quantified for (D) immune cells and (E) metabolites associated with fractures in the Mendelian randomization analysis. Figure S20: Top 5 GO enrichment analysis of SNP‐associated genes for MR of causality between immune cells/metabolites and fractures. The bubble diagram (upper section) illustrates gene enrichment pathways for five common GO pathways in MR analysis of causal SNPs between immune cells and metabolites. The lower section of the figure demonstrates gene enrichment pathways for five common GO pathways in MR analysis of causal SNPs between immune cells and fractures. The β value represents the effect size of the SNP in two‐sample MR. β1 represents the effect size of the SNP for immune cells and metabolites, and β3 represents the effect size of the SNP for metabolites and fractures. All pathways with *p*  < 0.05 were considered statistically significant. Figure S21: KEGG pathway of genes from the association between immune cells, metabolites, and fractures. Intersection of SNP‐associated gene enrichment KEGG pathways obtained from three MR analyses. β1 represents the effect size of the SNP for immune cells and metabolites, β2 represents the effect size of the SNP for immune cells and fractures, and β3 represents the effect size of the SNP for metabolites and fractures. All pathways with *p*  < 0.05 were considered statistically significant. Figure S22: PPI analysis of GO pathway‐related genes. The top 20 genes were assessed through four PPI analysis algorithms—degree, EPC, bottleneck, and closeness—resulting in the identification of nine critical genes. Figure S23: Assessing MR causality between CpG sites of hub genes and fractures (foot, excluding ankle). Exposure comprises CpG sites of hub genes, while the outcome is defined as fractures (foot, excluding ankle). nSNP: number of single nucleotide polymorphisms; method: inverse variance weighting; OR: odds ratio; CI: confidence interval. The odds ratio (OR) and confidence interval (CI) are calculated, with OR > 1 indicating that the exposure is a risk factor for the outcome and OR < 1 suggesting it serves as a protective factor. Heterogeneity is analyzed using Q, with Q_df representing the degrees of freedom; a Q_pval <0.05 indicates significant heterogeneity. The Egger_intercept is used for pleiotropy analysis, with E_se denoting the standard error. A *p*‐value (E_pval) <0.05 signifies the presence of pleiotropy. Figure S24: Assessing MR causality between CpG sites of hub genes and fractures (forea). Exposure comprises CpG sites of hub genes, while the outcome is defined as fractures (forea). nSNP: number of single nucleotide polymorphisms; method: inverse variance weighting; OR: odds ratio; CI: confidence interval. The odds ratio (OR) and confidence interval (CI) are calculated, with OR > 1 indicating that the exposure is a risk factor for the outcome and OR < 1 suggesting it serves as a protective factor. Heterogeneity is analyzed using Q, with Q_df representing the degrees of freedom; a Q_pval <0.05 indicates significant heterogeneity. The Egger_intercept is used for pleiotropy analysis, with E_se denoting the standard error. A *p*‐value (E_pval) <0.05 signifies the presence of pleiotropy. Figure S25: Assessing MR causality between CpG sites of hub genes and fractures (lower leg, including ankle). Exposure comprises CpG sites of hub genes, while the outcome is defined as fractures (lower leg, including ankle). nSNP: number of single nucleotide polymorphisms; method: inverse variance weighting; OR: odds ratio; CI: confidence interval. The odds ratio (OR) and confidence interval (CI) are calculated, with OR > 1 indicating that the exposure is a risk factor for the outcome and OR < 1 suggesting it serves as a protective factor. Heterogeneity is analyzed using Q, with Q_df representing the degrees of freedom; a Q_pval <0.05 indicates significant heterogeneity. The Egger_intercept is used for pleiotropy analysis, with E_se denoting the standard error. A *p*‐value (E_pval) <0.05 signifies the presence of pleiotropy. Figure S26: Assessing MR causality between CpG sites of hub genes and fractures (lumbar spine/pelvis). Exposure comprises CpG sites of hub genes, while the outcome is defined as fractures (lumbar spine/pelvis). nSNP: number of single nucleotide polymorphisms; method: inverse variance weighting; OR: odds ratio; CI: confidence interval. The odds ratio (OR) and confidence interval (CI) are calculated, with OR > 1 indicating that the exposure is a risk factor for the outcome and OR < 1 suggesting it serves as a protective factor. Heterogeneity is analyzed using Q, with Q_df representing the degrees of freedom; a Q_pval <0.05 indicates significant heterogeneity. The Egger_intercept is used for pleiotropy analysis, with E_se denoting the standard error. A *p*‐value (E_pval) <0.05 signifies the presence of pleiotropy. Figure S27: Assessing MR causality between CpG sites of hub genes and fractures (neck). Exposure comprises CpG sites of hub genes, while the outcome is defined as fractures (neck). nSNP: number of single nucleotide polymorphisms; method: inverse variance weighting; OR: odds ratio; CI: confidence interval. The odds ratio (OR) and confidence interval (CI) are calculated, with OR > 1 indicating that the exposure is a risk factor for the outcome, and OR < 1 suggesting it serves as a protective factor. Heterogeneity is analyzed using Q, with Q_df representing the degrees of freedom; a Q_pval <0.05 indicates significant heterogeneity. The Egger_intercept is used for pleiotropy analysis, with E_se denoting the standard error. A *p*‐value (E_pval) <0.05 signifies the presence of pleiotropy. Figure S28: Assessing MR causality between CpG sites of hub genes and fractures (ribs/sternum/thoracic spine). Exposure comprises CpG sites of hub genes, while the outcome is defined as fractures (ribs/sternum/thoracic spine). nSNP: number of single nucleotide polymorphisms; method: inverse variance weighting; OR: odds ratio; CI: confidence interval. The odds ratio (OR) and confidence interval (CI) are calculated, with OR > 1 indicating that the exposure is a risk factor for the outcome and OR < 1 suggesting it serves as a protective factor. Heterogeneity is analyzed using Q, with Q_df representing the degrees of freedom; a Q_pval <0.05 indicates significant heterogeneity. The Egger_intercept is used for pleiotropy analysis, with E_se denoting the standard error. A *p*‐value (E_pval) <0.05 signifies the presence of pleiotropy. Figure S29: Assessing MR causality between CpG sites of hub genes and fractures (shoulder/upper arm). Exposure comprises CpG sites of hub genes, while the outcome is defined as fractures (shoulder/upper arm). nSNP: number of single nucleotide polymorphisms; method: inverse variance weighting; OR: odds ratio; CI: confidence interval. The odds ratio (OR) and confidence interval (CI) are calculated, with OR > 1 indicating that the exposure is a risk factor for the outcome and OR < 1 suggesting it serves as a protective factor. Heterogeneity is analyzed using Q, with Q_df representing the degrees of freedom; a Q_pval <0.05 indicates significant heterogeneity. The Egger_intercept is used for pleiotropy analysis, with E_se denoting the standard error. A *p*‐value (E_pval) <0.05 signifies the presence of pleiotropy. Figure S30: Assessing MR causality between CpG sites of hub genes and fractures (skull/facial bones). Exposure comprises CpG sites of hub genes, while the outcome is defined as fractures (skull/facial bones). nSNP: number of single nucleotide polymorphisms; method: inverse variance weighting; OR: odds ratio; CI: confidence interval. The odds ratio (OR) and confidence interval (CI) are calculated, with OR > 1 indicating that the exposure is a risk factor for the outcome and OR < 1 suggesting it serves as a protective factor. Heterogeneity is analyzed using Q, with Q_df representing the degrees of freedom; a Q_pval <0.05 indicates significant heterogeneity. The Egger_intercept is used for pleiotropy analysis, with E_se denoting the standard error. A *p*‐value (E_pval) <0.05 signifies the presence of pleiotropy. Figure S31: Assessing MR causality between CpG sites of hub genes and fractures (wrists). Exposure comprises CpG sites of hub genes, while the outcome is defined as fractures (wrist). nSNP: number of single nucleotide polymorphisms; method: inverse variance weighting; OR: odds ratio; CI: confidence interval. The odds ratio (OR) and confidence interval (CI) are calculated, with OR > 1 indicating that the exposure is a risk factor for the outcome and OR < 1 suggesting it serves as a protective factor. Heterogeneity is analyzed using Q, with Q_df representing the degrees of freedom; a Q_pval <0.05 indicates significant heterogeneity. The Egger_intercept is used for pleiotropy analysis, with E_se denoting the standard error. A *p*‐value (E_pval) <0.05 signifies the presence of pleiotropy.

**Supporting Information 2** Table S1: Assessing MR causality between immune cells and fractures by inverse variance weighting (IVW) method. FDR (BH‐adjusted *p*‐value) = *P*× (268/Rank of *p*). Table S2: Assessing MR causality between Metabolites and fracturesby inverse variance weighting (IVW) method. FDR (BH‐adjusted *p*‐value) = *P*× (528/Rank of *p*). Table S3: Four methods of foot (except ankle) and immune cells in MR analysis. Table S4: Four methods of forea and immune cells in MR analysis. Table S5: Four methods of lower leg (including ankle) and immune cells in MR analysis. Table S6: Four methods of lumbar spine/pelvis and immune cells in MR analysis. Table S7: Four methods of neck and immune cells in MR analysis. Table S8: Four methods of ribs/sternum/thoracic spine and immune cells in MR analysis. Table S9: Four methods of shoulder/upper arm and immune cells in MR analysis. Table S10: Four methods of skull/facial bones and immune cells in MR analysis. Table S11: Four methods of wrist and immune cells in MR analysis. Table S12: Four methods of foot (except ankle) and metabolites in MR analysis. Table S13: Four methods of forea and metabolites in MR analysis. Table S14: Four methods of lower leg (including ankle) and metabolites in MR analysis. Table S15: Four methods of lumbar spine/pelvis and metabolites in MR analysis. Table S16: Four methods of neck and metabolites in MR analysis. Table S17: Four methods of ribs/sternum/thoracic spine and metabolites in MR analysis. Table S18: Four methods of shoulder/upper arm and metabolites in MR analysis. Table S19: Four methods of skull/facial bones and metabolites in MR analysis. Table S20: Four methods of wrist and metabolites in MR analysis. Table S21: Five methods of foot (except ankle) and immune cells in reverse MR analysis. Table S22: Five methods of forea and immune cells in reverse MR analysis. Table S23: Five methods of lower leg (including ankle) and immune cells in reverse MR analysis. Table S24: Five methods of lumbar spine/pelvis and immune cells in reverse MR analysis. Table S25: Five methods of neck and immune cells in reverse MR analysis. Table S26: Five methods of ribs/sternum/thoracic spine and immune cells in reverse MR analysis. Table S27: Five methods of shoulder/upper arm and immune cells in reverse MR analysis. Table S28: Five methods of skull/facial bones and immune cells in reverse MR analysis. Table S29: Five methods of wrist and immune cells in reverse MR analysis. Table S30: Five methods of foot (except ankle) and metabolites in reverse MR analysis. Table S31: Five methods of forea and metabolites in reverse MR analysis. Table S32: Five methods of lower leg (including ankle) and metabolites in reverse MR analysis. Table S33: Five methods of lumbar spine/pelvis and metabolites in reverse MR analysis. Table S34: Five methods of neck and metabolites in reverse MR analysis. Table S35: Five methods of ribs/sternum/thoracic spine and metabolites in reverse MR analysis. Table S36: Five methods of shoulder/upper arm and metabolites in reverse MR analysis. Table S37: Five methods of skull/facial bones and metabolites in reverse MR analysis. Table S38: Five methods of wrist and metabolites in reverse MR analysis. Table S39: IVW method of immune cells and metabolites in MR analysis associated with fracture of foot (except ankle). Table S40: IVW method of immune cells and metabolites in MR analysis associated with fracture of forea. Table S41: IVW method of immune cells and metabolites in MR analysis associated with fracture of lower leg (including ankle). Table S42: IVW method of immune cells and metabolites in MR analysis associated with fracture of lumbar spine/pelvis. Table S43: IVW method of immune cells and metabolites in MR analysis associated with fracture of neck. Table S44: IVW method of immune cells and metabolites in MR analysis associated with fracture of ribs/sternum/thoracic spine. Table S45: IVW method of immune cells and metabolites in MR analysis associated with fracture of shoulder/upper arm. Table S46: IVW method of immune cells and metabolites in MR analysis associated with fracture of skull/facial bones. Table S47: IVW method of immune cells and metabolites in MR analysis associated with fracture of wrist. Table S48: Metabolites as mediators of MR causation effects in immune cells and fractures. Table S49: immune cells causally SNPs nearest genes associated with metabolites. Table S50: immune cells causally SNPs nearest genes associated with fractures. Table S51: metabolites causally SNPs nearest genes associated with fractures. Table S52: SNP nearest gene in MR analysis. Table S53: KEGG analysis of SNP‐associated genes for MR of causality between immune cells and various sites of fracture (FDR < 0.05). Table S54: GO analysis of SNP‐associated genes for MR of causality between immune cells and various sites of fracture (FDR < 0.05). Table S55: KEGG analysis of SNP‐associated genes for MR of causality between metabolites and various sites of fracture (FDR < 0.05)Table S56: GO analysis of SNP‐associated genes for MR of causality between metabolites and various sites of fracture (FDR < 0.05). Table S57: KEGG analysis of SNP‐associated genes for MR of causality between immune cells and metabolites (FDR < 0.05). Table S58: GO analysis of SNP‐associated genes for MR of causality immune cells between and metabolites (FDR < 0.05). Table S59: Summary of 6 gene eQTL data for Colocalization analysis. Table S60: Summary of 36 fracture GWAS data for Colocalization analysis. Table S61: Colocalization analysis between hub genes and fractures. Table S62: Hub gene‐related methylation sites. Table S63: Four methods of foot (except ankle) and CpG sites of hub gene in MR analysis. Table S64: Four methods of forea and CpG sites of hub gene in MR analysis. Table S65: Four methods of lower leg (including ankle) and CpG sites of hub gene in MR analysis. Table S66: Four methods of lumbar spine/pelvis and CpG sites of hub gene in MR analysis. Table S67: Four methods of neck and CpG sites of hub gene in MR analysis. Table S68: Four methods of ribs/sternum/thoracic spine and CpG sites of hub gene in MR analysis. Table S69: Four methods of shoulder/upper arm and CpG sites of hub gene in MR analysis. Table S70: Four methods of skull/facial bones and CpG sites of hub gene in MR analysis. Table S71: Four methods of wrist and CpG sites of hub gene in MR analysis.

## Data Availability

The datasets used in this study were sourced from publicly available databases. The findings and results of the analyses are thoroughly documented in the article and Supporting Information.
