# Supplementary material for: Unveiling Epigenetic Molecular Mechanisms in Bone Fracture Risk: Insights From 731 Immune Cells, 1400 Metabolites, and Critical Genetic Pathways
Source: Mediators Inflamm. 2026 Apr 10;2026:3846184. doi: 10.1155/mi/3846184 (PMC13067064; doi:10.1155/mi/3846184)
Supplement: Supplementary file 1 — Supporting Information 1 Figure S1: Assessing MR causality between immune cells and fractures (foot, excluding ankle). Exposure comprises 731 immune cell phenotypes, while the outcome is defined as fractures (foot, excluding ankle); nSNP: number of single nucleotide polymorphisms; method: inverse variance weighting; OR: odds ratio; CI: confidence interval. The odds ratio (OR) and confidence interval (CI) are calculated, with OR > 1 indicating that the exposure is a risk factor for the outcome and OR < 1 suggesting it serves as a protective factor. Heterogeneity is analyzed using Q, with Q_df representing the degrees of freedom; a Q_pval <0.05 indicates significant heterogeneity. The Egger_intercept is used for pleiotropy analysis, with E_se denoting the standard error. A p‐value (E_pval) <0.05 signifies the presence of pleiotropy. Figure S2: Assessing MR causality between immune cells and fractures (forea). Exposure comprises 731 immune cell phenotypes, while the outcome is defined as fractures (forea); nSNP: number of single nucleotide polymorphisms; method: inverse variance weighting; OR: odds ratio; CI: confidence interval. The odds ratio (OR) and confidence interval (CI) are calculated, with OR > 1 indicating that the exposure is a risk factor for the outcome and OR < 1 suggesting it serves as a protective factor. Heterogeneity is analyzed using Q, with Q_df representing the degrees of freedom; a Q_pval <0.05 indicates significant heterogeneity. The Egger_intercept is used for pleiotropy analysis, with E_se denoting the standard error. A p‐value (E_pval) <0.05 signifies the presence of pleiotropy. Figure S3: Assessing MR causality between immune cells and fractures (lower leg, including ankle). Exposure comprises 731 immune cell phenotypes, while the outcome is defined as fractures (lower leg, including ankle); nSNP: number of single nucleotide polymorphisms; method: inverse variance weighting; OR: odds ratio; CI: confidence interval. The odds ratio (OR) and con [file MI-2026-3846184-s001.docx]

**Unveiling Epigenetic Molecular Mechanisms in Bone Fracture Risk: Insights from 731 Immune Cells, 1400 Metabolites, and Critical Genetic Pathways**

**
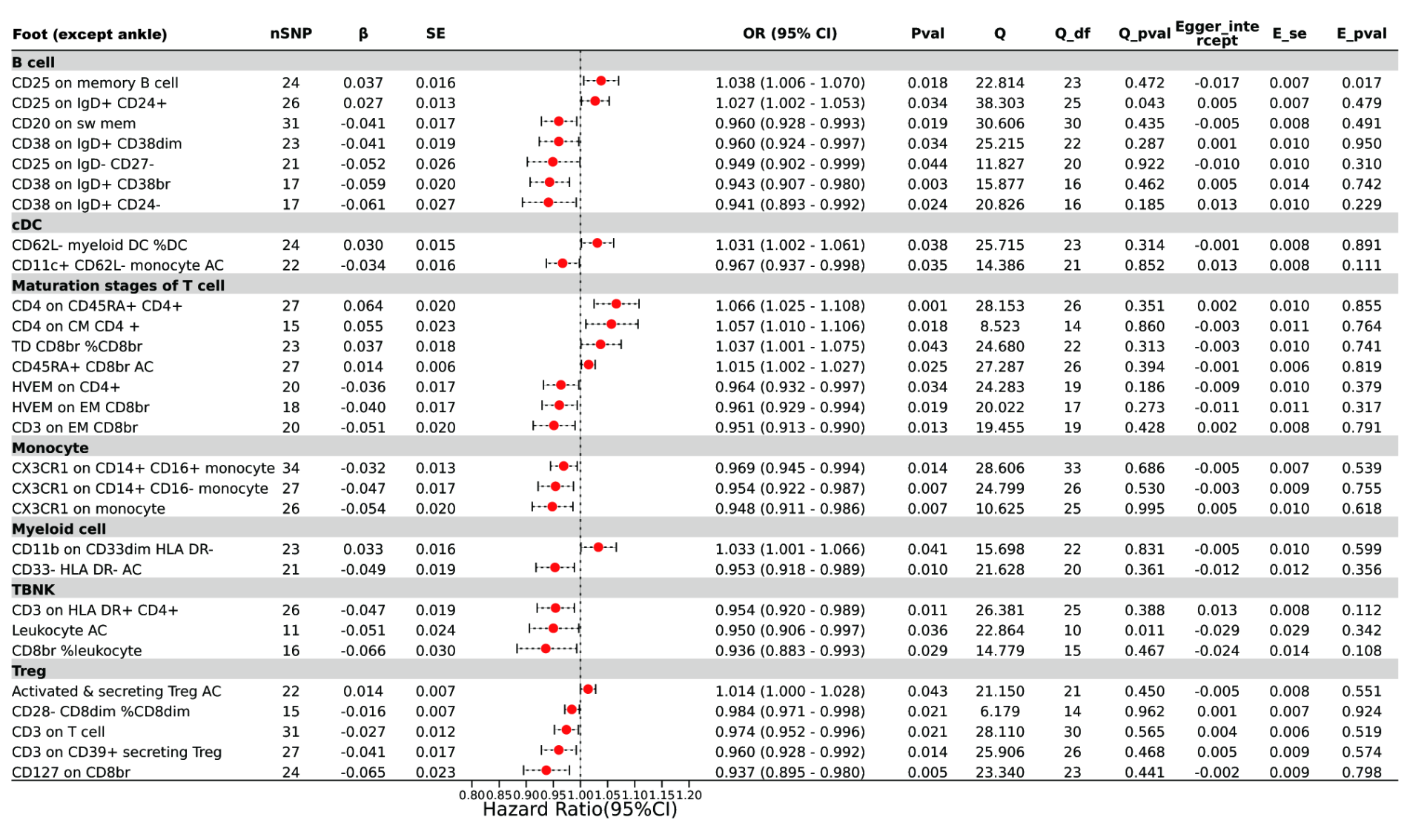
**

**Figure S1: Assessing MR causality between immune cells and fractures (foot, excluding ankle).** Exposure comprises 731 immune cell phenotypes, while the outcome is defined as fractures (foot, excluding ankle); nSNP: number of single nucleotide polymorphisms; method: inverse variance weighting; OR: odds ratio; CI: confidence interval. The odds ratio (OR) and confidence interval (CI) are calculated, with OR > 1 indicating that the exposure is a risk factor for the outcome, and OR < 1 suggesting it serves as a protective factor. Heterogeneity is analyzed using Q, with Q_df representing the degrees of freedom; a Q_pval < 0.05 indicates significant heterogeneity. The Egger_intercept is used for pleiotropy analysis, with E_se denoting the standard error. A p-value (E_pval) < 0.05 signifies the presence of pleiotropy.
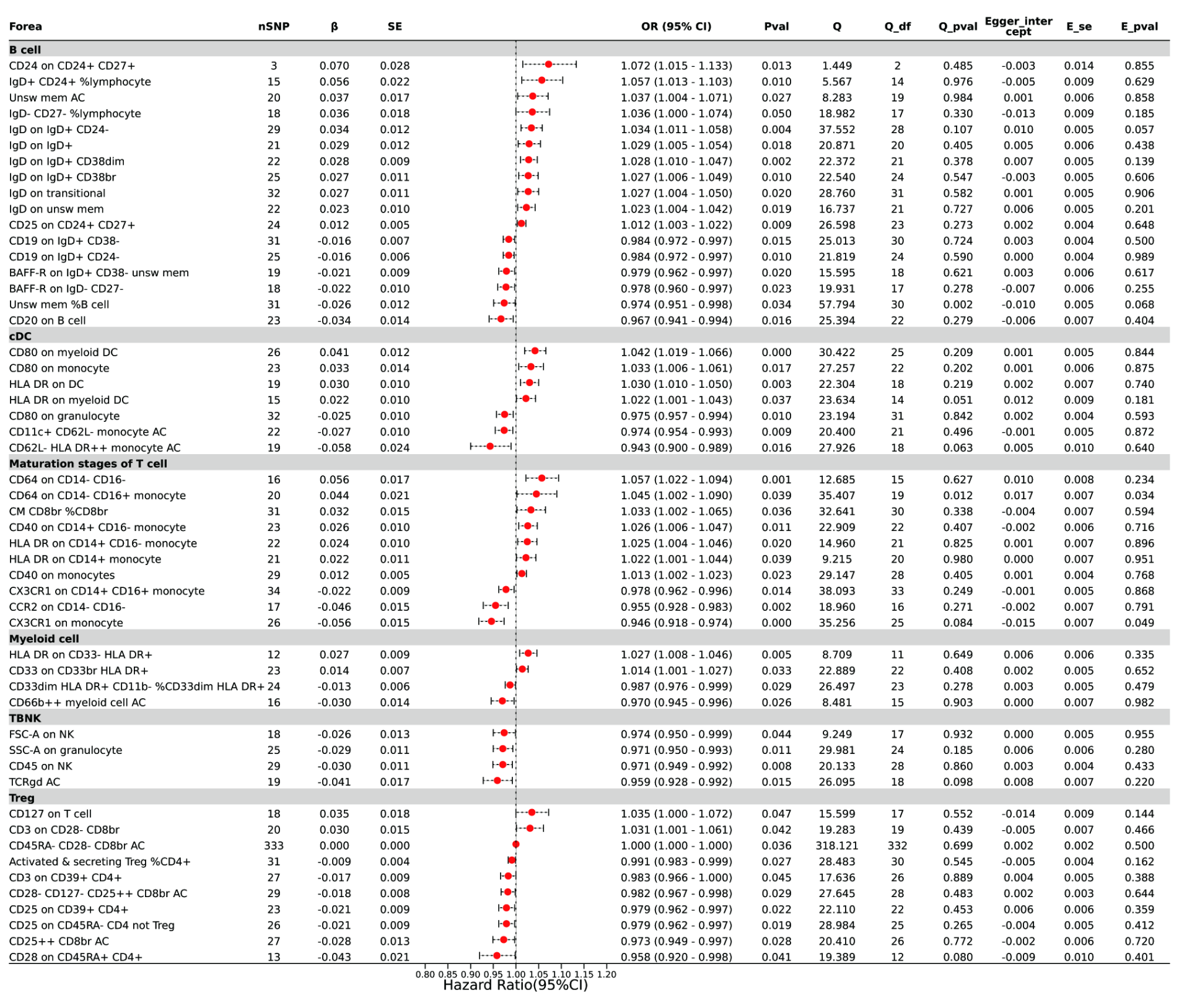


**Figure S2: Assessing MR causality between immune cells and fractures (forea).** Exposure comprises 731 immune cell phenotypes, while the outcome is defined as fractures (forea); nSNP: number of single nucleotide polymorphisms; method: inverse variance weighting; OR: odds ratio; CI: confidence interval. The odds ratio (OR) and confidence interval (CI) are calculated, with OR > 1 indicating that the exposure is a risk factor for the outcome, and OR < 1 suggesting it serves as a protective factor. Heterogeneity is analyzed using Q, with Q_df representing the degrees of freedom; a Q_pval < 0.05 indicates significant heterogeneity. The Egger_intercept is used for pleiotropy analysis, with E_se denoting the standard error. A p-value (E_pval) < 0.05 signifies the presence of pleiotropy.


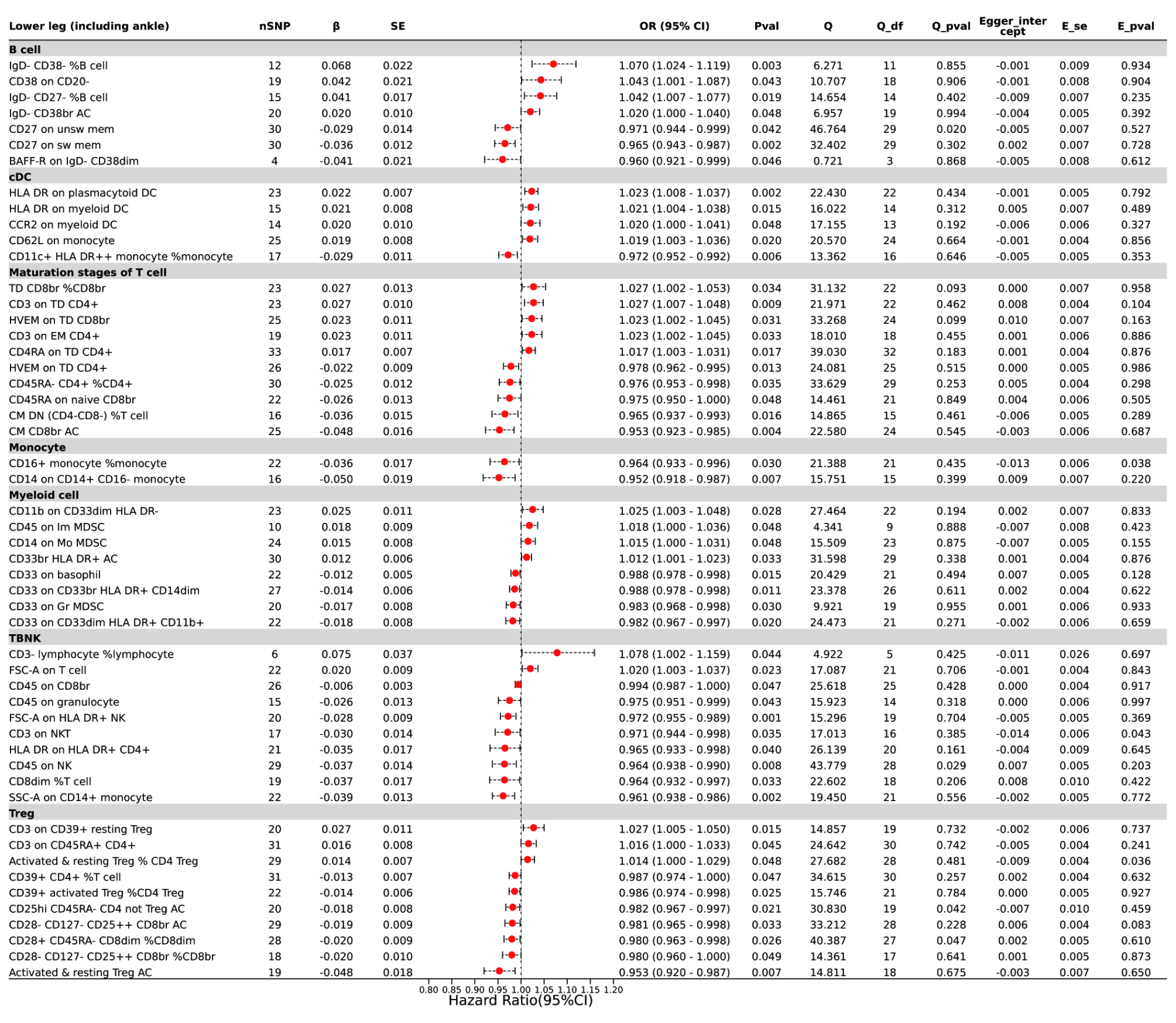


**Figure S3: Assessing MR causality between immune cells and fractures (lower leg, including ankle).** Exposure comprises 731 immune cell phenotypes, while the outcome is defined as fractures (lower leg, including ankle); nSNP: number of single nucleotide polymorphisms; method: inverse variance weighting; OR: odds ratio; CI: confidence interval. The odds ratio (OR) and confidence interval (CI) are calculated, with OR > 1 indicating that the exposure is a risk factor for the outcome, and OR < 1 suggesting it serves as a protective factor. Heterogeneity is analyzed using Q, with Q_df representing the degrees of freedom; a Q_pval < 0.05 indicates significant heterogeneity. The Egger_intercept is used for pleiotropy analysis, with E_se denoting the standard error. A p-value (E_pval) < 0.05 signifies the presence of pleiotropy.


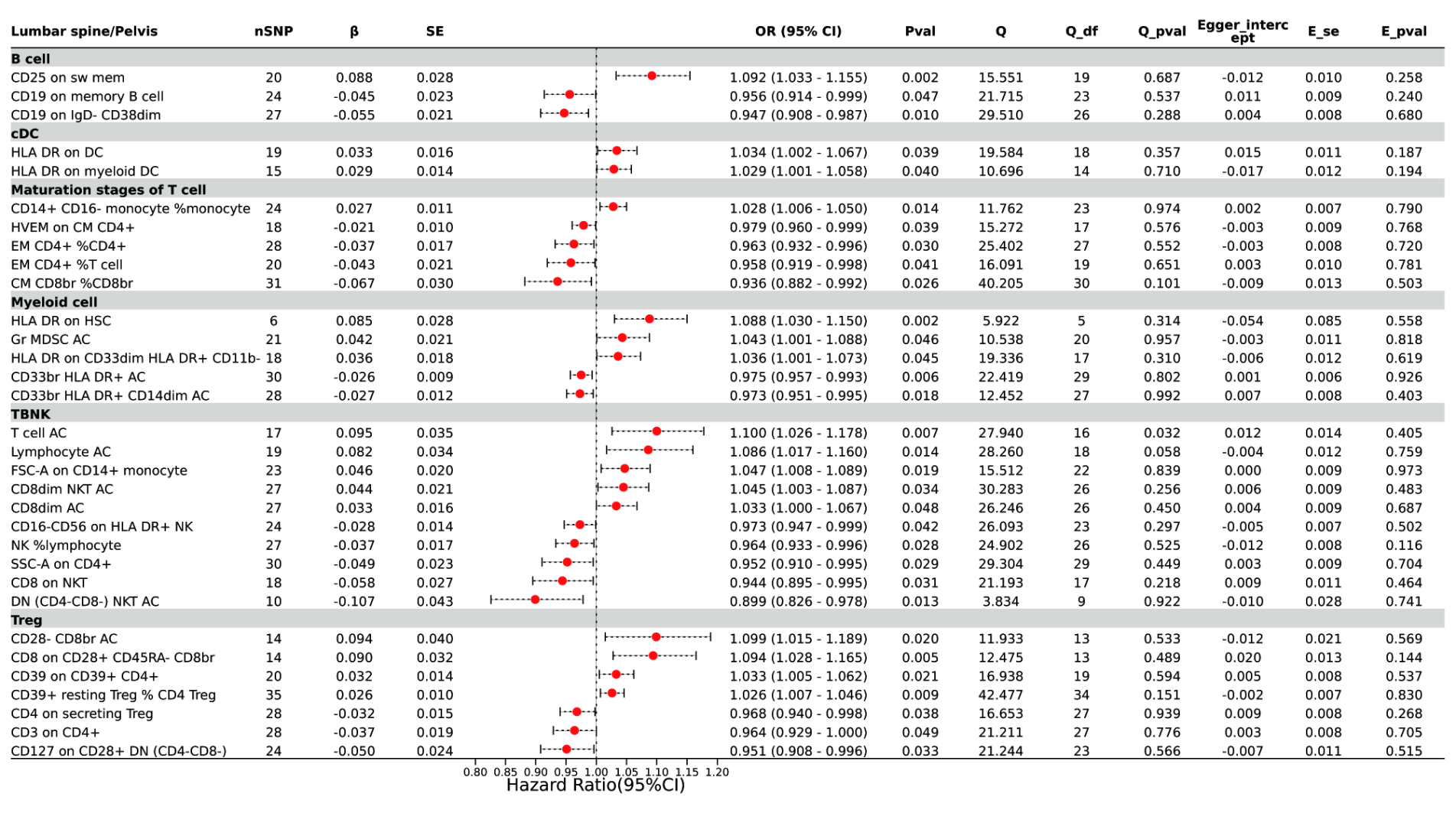


**Figure S4: Assessing MR causality between immune cells and fractures (lumber spine/pelvis).** Exposure comprises 731 immune cell phenotypes, while the outcome is defined as fractures (lumber spine/pelvis); nSNP: number of single nucleotide polymorphisms; method: inverse variance weighting; OR: odds ratio; CI: confidence interval. The odds ratio (OR) and confidence interval (CI) are calculated, with OR > 1 indicating that the exposure is a risk factor for the outcome, and OR < 1 suggesting it serves as a protective factor. Heterogeneity is analyzed using Q, with Q_df representing the degrees of freedom; a Q_pval < 0.05 indicates significant heterogeneity. The Egger_intercept is used for pleiotropy analysis, with E_se denoting the standard error. A p-value (E_pval) < 0.05 signifies the presence of pleiotropy.

**
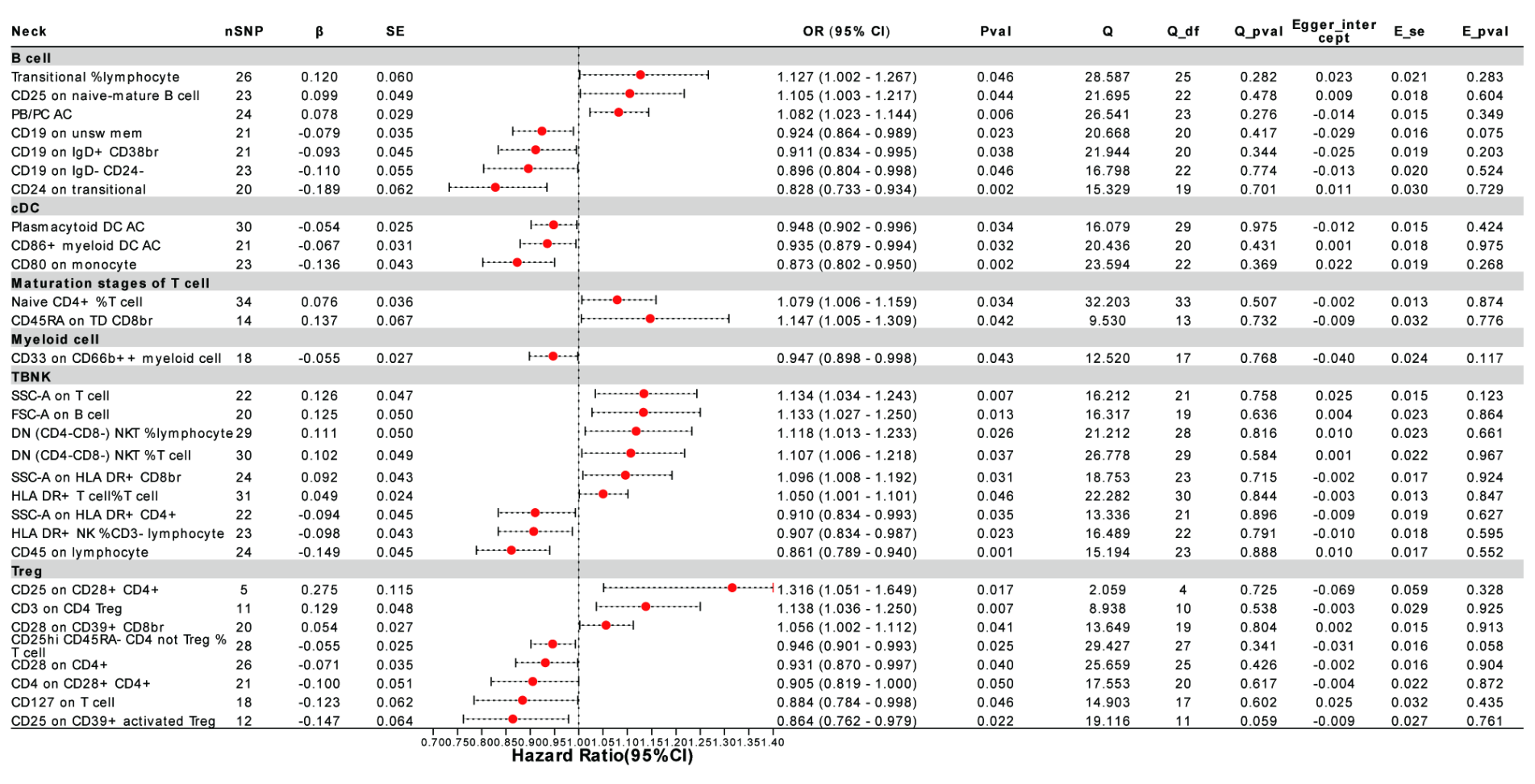
**

**Figure S5: Assessing MR causality between immune cells and fractures (neck).** Exposure comprises 731 immune cell phenotypes, while the outcome is defined as fractures (neck); nSNP: number of single nucleotide polymorphisms; method: inverse variance weighting; OR: odds ratio; CI: confidence interval. The odds ratio (OR) and confidence interval (CI) are calculated, with OR > 1 indicating that the exposure is a risk factor for the outcome, and OR < 1 suggesting it serves as a protective factor. Heterogeneity is analyzed using Q, with Q_df representing the degrees of freedom; a Q_pval < 0.05 indicates significant heterogeneity. The Egger_intercept is used for pleiotropy analysis, with E_se denoting the standard error. A p-value (E_pval) < 0.05 signifies the presence of pleiotropy.


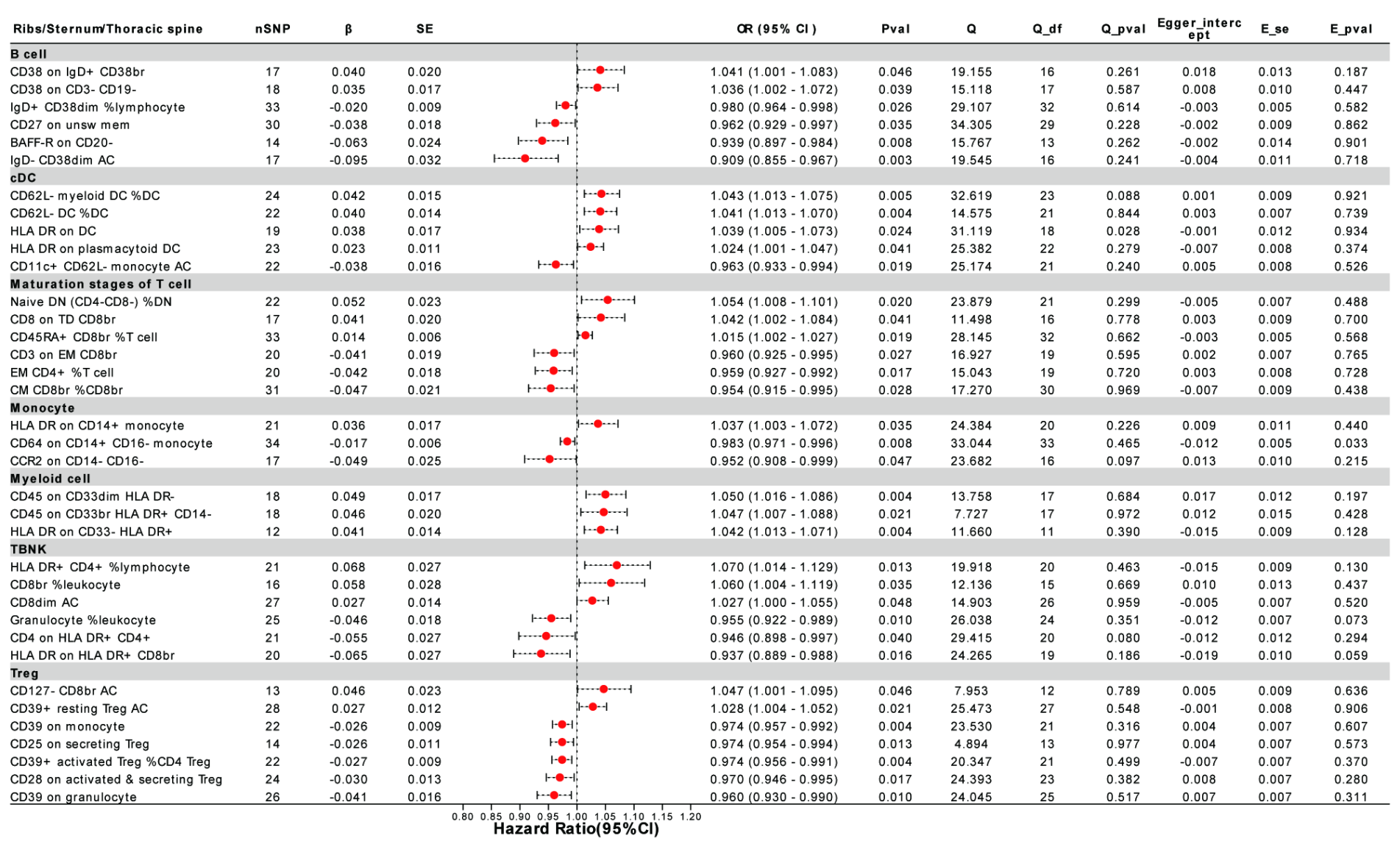


**Figure S6: Assessing MR causality between immune cells and fractures (ribs/sternum/thoracic spine).** Exposure comprises 731 immune cell phenotypes, while the outcome is defined as fractures (ribs/sternum/thoracic spine); nSNP: number of single nucleotide polymorphisms; method: inverse variance weighting; OR: odds ratio; CI: confidence interval. The odds ratio (OR) and confidence interval (CI) are calculated, with OR > 1 indicating that the exposure is a risk factor for the outcome, and OR < 1 suggesting it serves as a protective factor. Heterogeneity is analyzed using Q, with Q_df representing the degrees of freedom; a Q_pval < 0.05 indicates significant heterogeneity. The Egger_intercept is used for pleiotropy analysis, with E_se denoting the standard error. A p-value (E_pval) < 0.05 signifies the presence of pleiotropy.


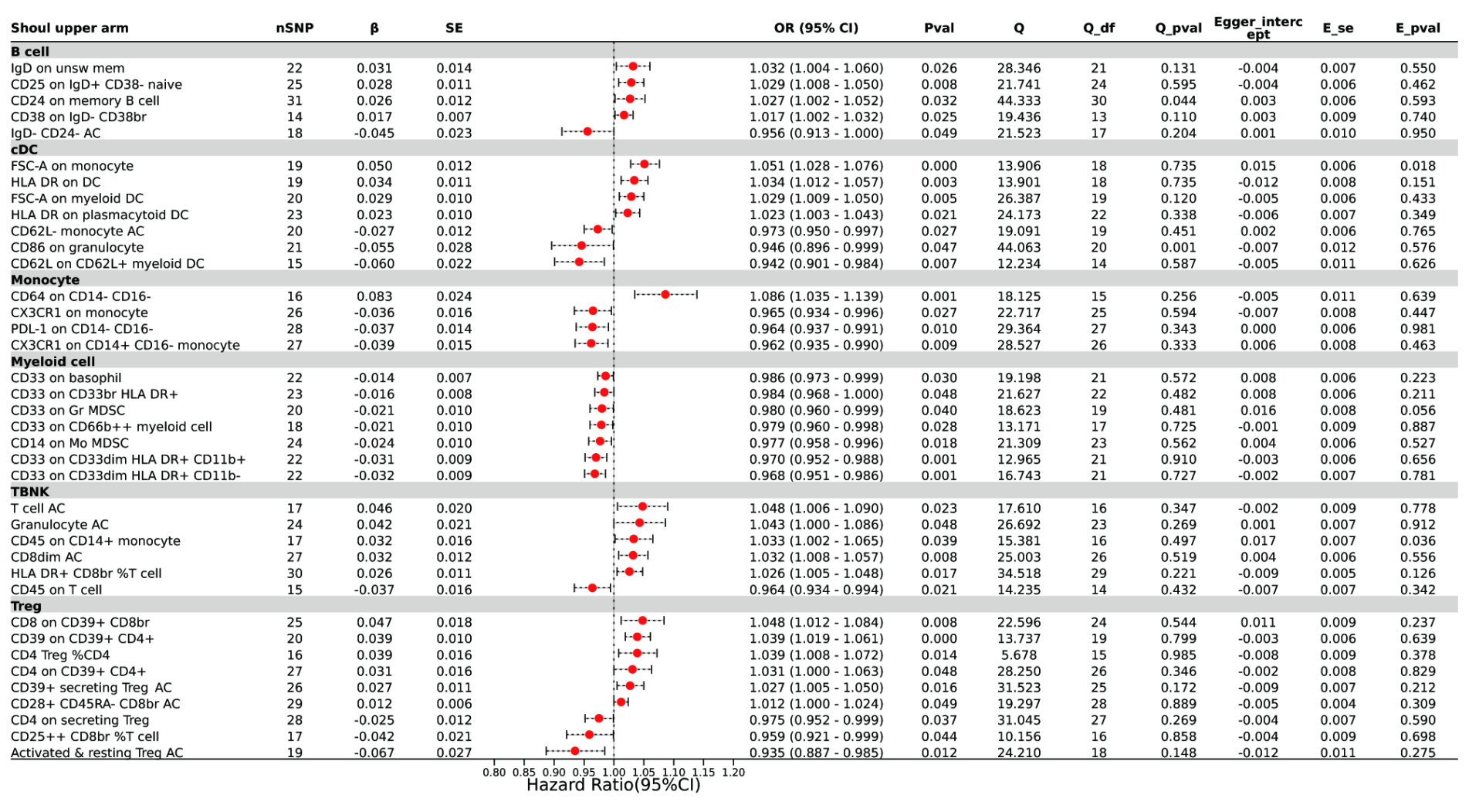


**Figure S7: Assessing MR causality between immune cells and fractures (shoul upper arm).** Exposure comprises 731 immune cell phenotypes, while the outcome is defined as fractures (shoul upper arm); nSNP: number of single nucleotide polymorphisms; method: inverse variance weighting; OR: odds ratio; CI: confidence interval. The odds ratio (OR) and confidence interval (CI) are calculated, with OR > 1 indicating that the exposure is a risk factor for the outcome, and OR < 1 suggesting it serves as a protective factor. Heterogeneity is analyzed using Q, with Q_df representing the degrees of freedom; a Q_pval < 0.05 indicates significant heterogeneity. The Egger_intercept is used for pleiotropy analysis, with E_se denoting the standard error. A p-value (E_pval) < 0.05 signifies the presence of pleiotropy.


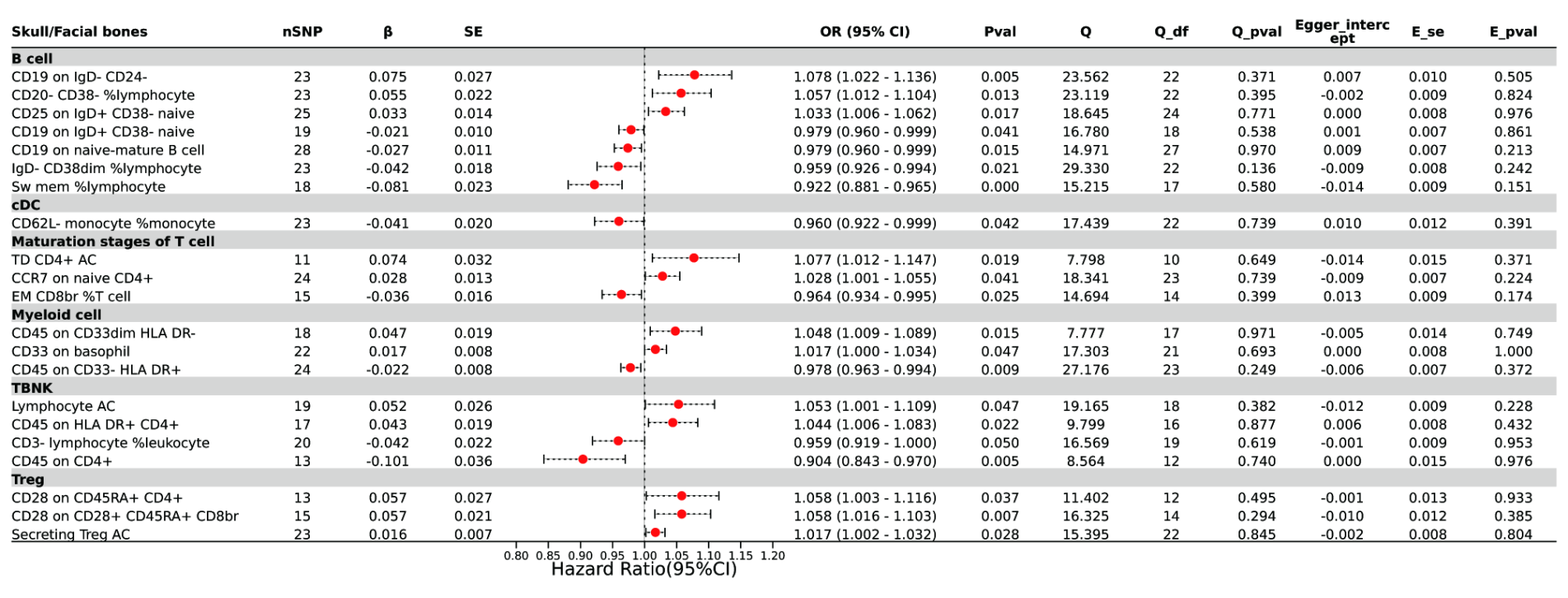


**Figure S8: Assessing MR causality between immune cells and fractures (skull/facial bones).** Exposure comprises 731 immune cell phenotypes, while the outcome is defined as fractures (skull/facial bones); nSNP: number of single nucleotide polymorphisms; method: inverse variance weighting; OR: odds ratio; CI: confidence interval. The odds ratio (OR) and confidence interval (CI) are calculated, with OR > 1 indicating that the exposure is a risk factor for the outcome, and OR < 1 suggesting it serves as a protective factor. Heterogeneity is analyzed using Q, with Q_df representing the degrees of freedom; a Q_pval < 0.05 indicates significant heterogeneity. The Egger_intercept is used for pleiotropy analysis, with E_se denoting the standard error. A p-value (E_pval) < 0.05 signifies the presence of pleiotropy.


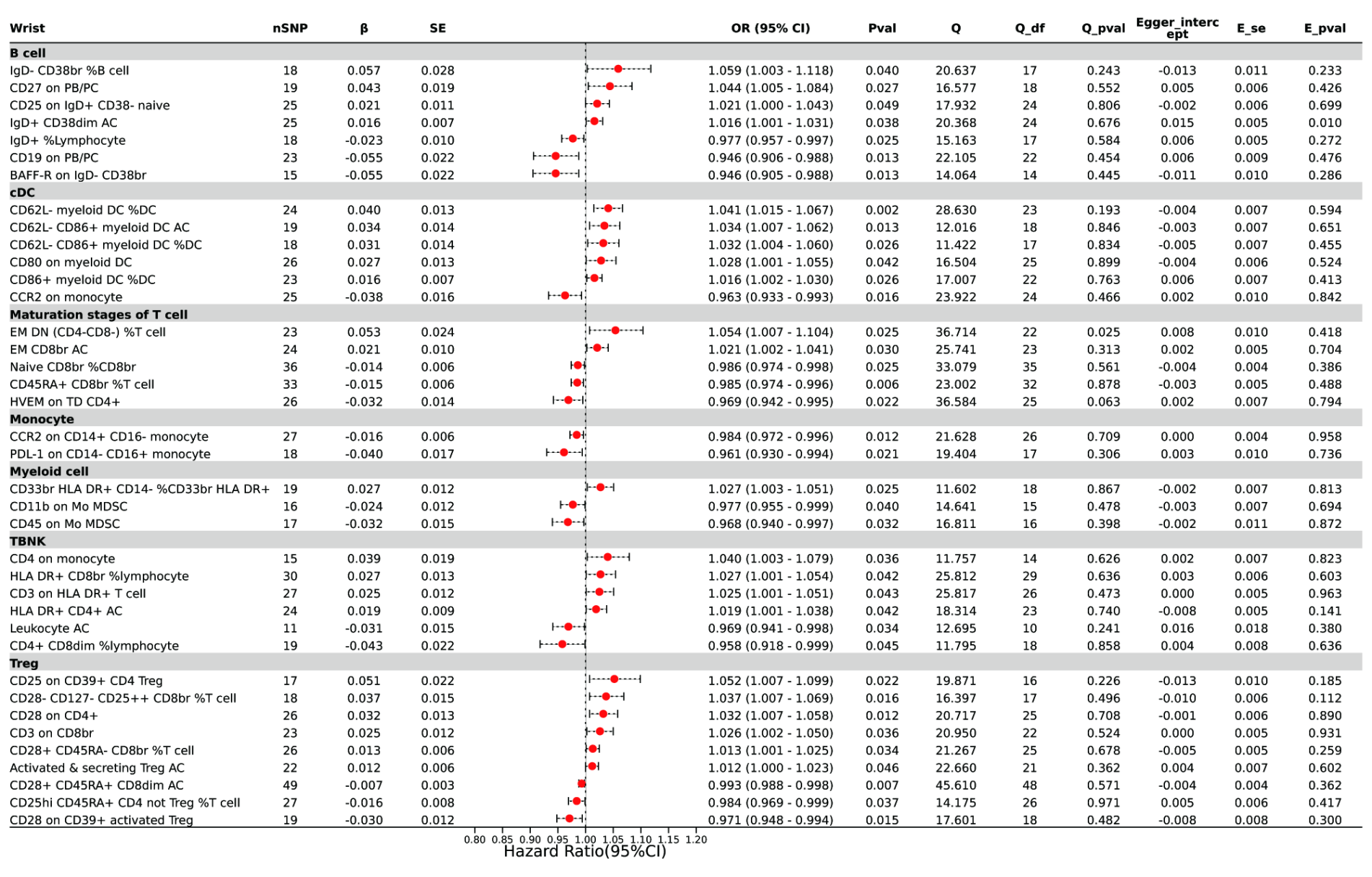


**Figure S9: Assessing MR causality between immune cells and fractures (wrist).** Exposure comprises 731 immune cell phenotypes, while the outcome is defined as fractures (wrist); nSNP: number of single nucleotide polymorphisms; method: inverse variance weighting; OR: odds ratio; CI: confidence interval. The odds ratio (OR) and confidence interval (CI) are calculated, with OR > 1 indicating that the exposure is a risk factor for the outcome, and OR < 1 suggesting it serves as a protective factor. Heterogeneity is analyzed using Q, with Q_df representing the degrees of freedom; a Q_pval < 0.05 indicates significant heterogeneity. The Egger_intercept is used for pleiotropy analysis, with E_se denoting the standard error. A p-value (E_pval) < 0.05 signifies the presence of pleiotropy.


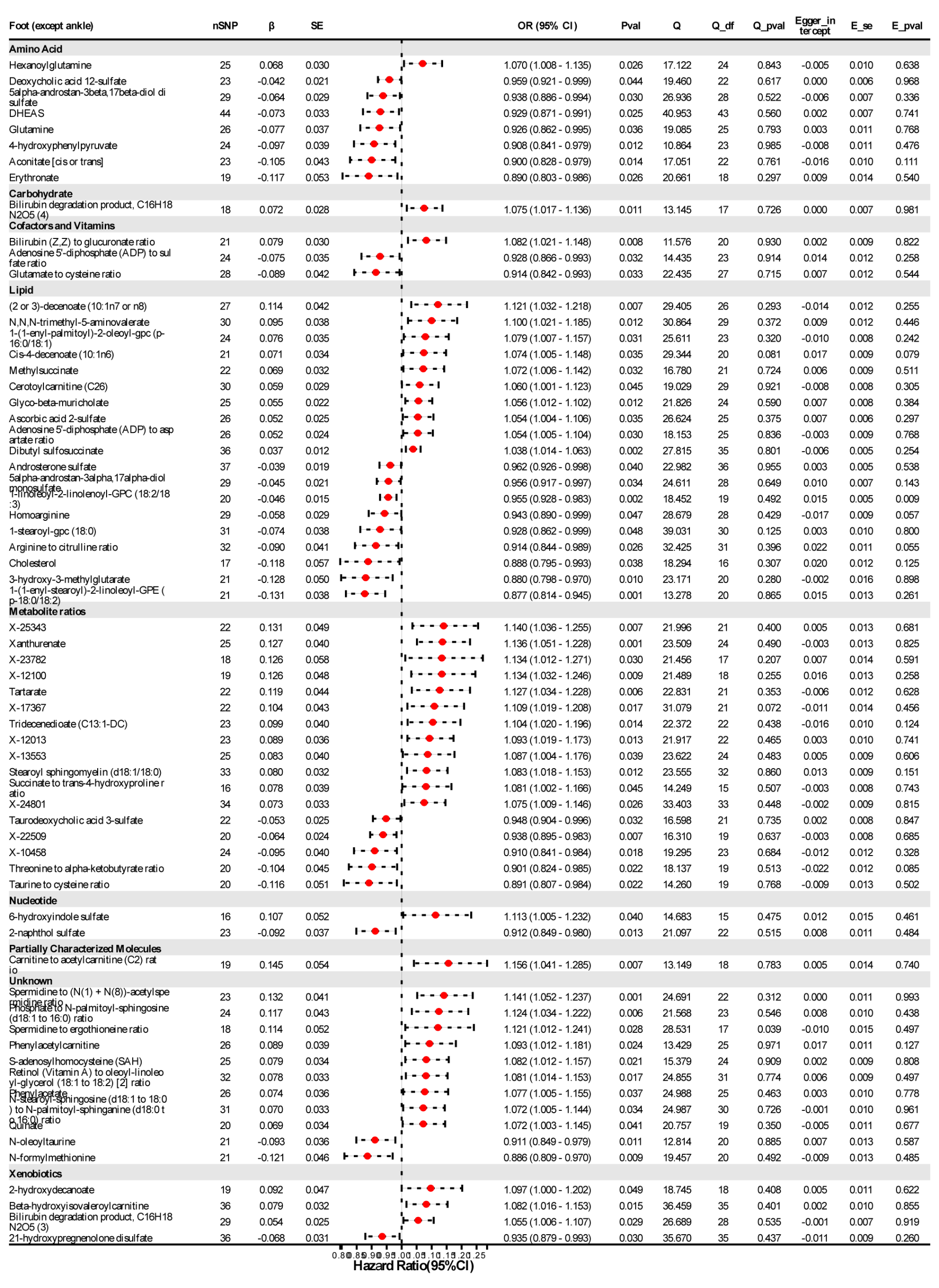


**Figure S10: Assessing MR causality between metabolites and fractures (foot, excluding ankle).** Exposure comprises 1400 metabolites, while the outcome is defined as fractures (foot, excluding ankle); nSNP: number of single nucleotide polymorphisms; method: inverse variance weighting; OR: odds ratio; CI: confidence interval. The odds ratio (OR) and confidence interval (CI) are calculated, with OR > 1 indicating that the exposure is a risk factor for the outcome, and OR < 1 suggesting it serves as a protective factor. Heterogeneity is analyzed using Q, with Q_df representing the degrees of freedom; a Q_pval < 0.05 indicates significant heterogeneity. The Egger_intercept is used for pleiotropy analysis, with E_se denoting the standard error. A p-value (E_pval) < 0.05 signifies the presence of pleiotropy.

**
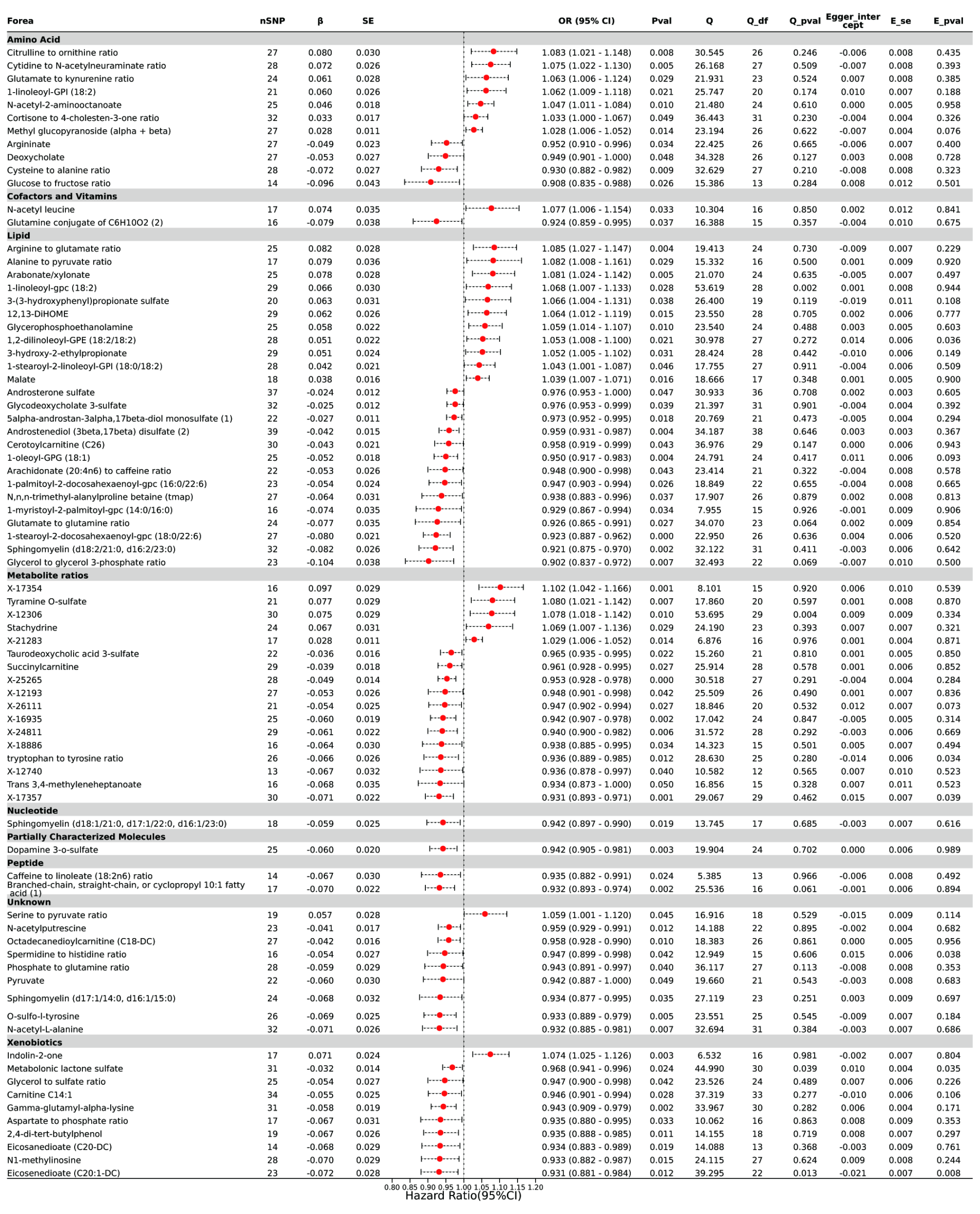
**

**Figure S11: Assessing MR causality between metabolites and fractures (forea).** Exposure comprises 1400 metabolites, while the outcome is defined as fractures (forea); nSNP: number of single nucleotide polymorphisms; method: inverse variance weighting; OR: odds ratio; CI: confidence interval. The odds ratio (OR) and confidence interval (CI) are calculated, with OR > 1 indicating that the exposure is a risk factor for the outcome, and OR < 1 suggesting it serves as a protective factor. Heterogeneity is analyzed using Q, with Q_df representing the degrees of freedom; a Q_pval < 0.05 indicates significant heterogeneity. The Egger_intercept is used for pleiotropy analysis, with E_se denoting the standard error. A p-value (E_pval) < 0.05 signifies the presence of pleiotropy.


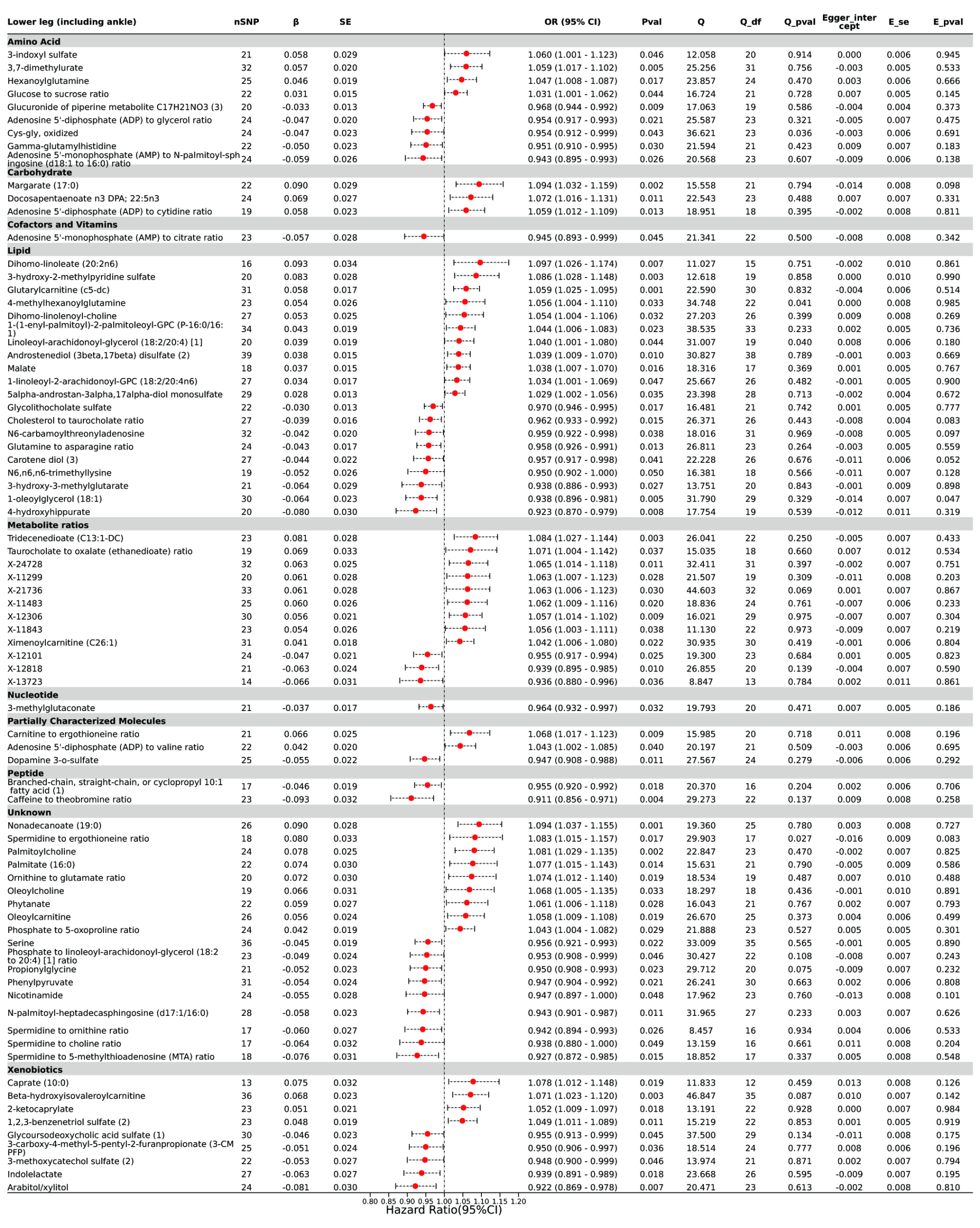


**Figure S12: Assessing MR causality between metabolites and fractures (lower leg, including ankle).** Exposure comprises 1400 metabolites, while the outcome is defined as fractures (lower leg, including ankle); nSNP: number of single nucleotide polymorphisms; method: inverse variance weighting; OR: odds ratio; CI: confidence interval. The odds ratio (OR) and confidence interval (CI) are calculated, with OR > 1 indicating that the exposure is a risk factor for the outcome, and OR < 1 suggesting it serves as a protective factor. Heterogeneity is analyzed using Q, with Q_df representing the degrees of freedom; a Q_pval < 0.05 indicates significant heterogeneity. The Egger_intercept is used for pleiotropy analysis, with E_se denoting the standard error. A p-value (E_pval) < 0.05 signifies the presence of pleiotropy.


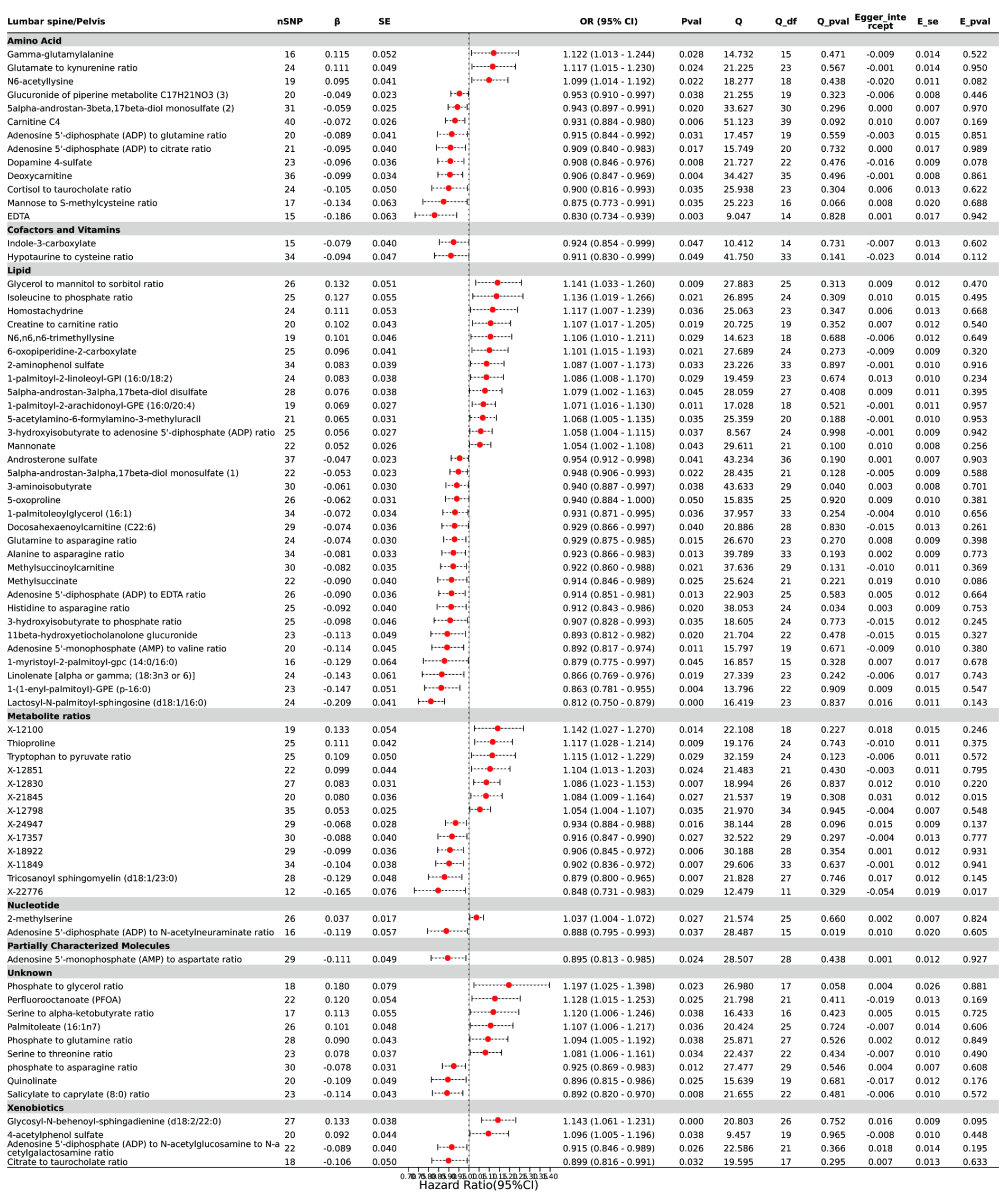


**Figure S13: Assessing MR causality between metabolites and fractures (lumbar spine/pelvis).** Exposure comprises 1400 metabolites, while the outcome is defined as fractures (lumbar spine/pelvis); nSNP: number of single nucleotide polymorphisms; method: inverse variance weighting; OR: odds ratio; CI: confidence interval. The odds ratio (OR) and confidence interval (CI) are calculated, with OR > 1 indicating that the exposure is a risk factor for the outcome, and OR < 1 suggesting it serves as a protective factor. Heterogeneity is analyzed using Q, with Q_df representing the degrees of freedom; a Q_pval < 0.05 indicates significant heterogeneity. The Egger_intercept is used for pleiotropy analysis, with E_se denoting the standard error. A p-value (E_pval) < 0.05 signifies the presence of pleiotropy.


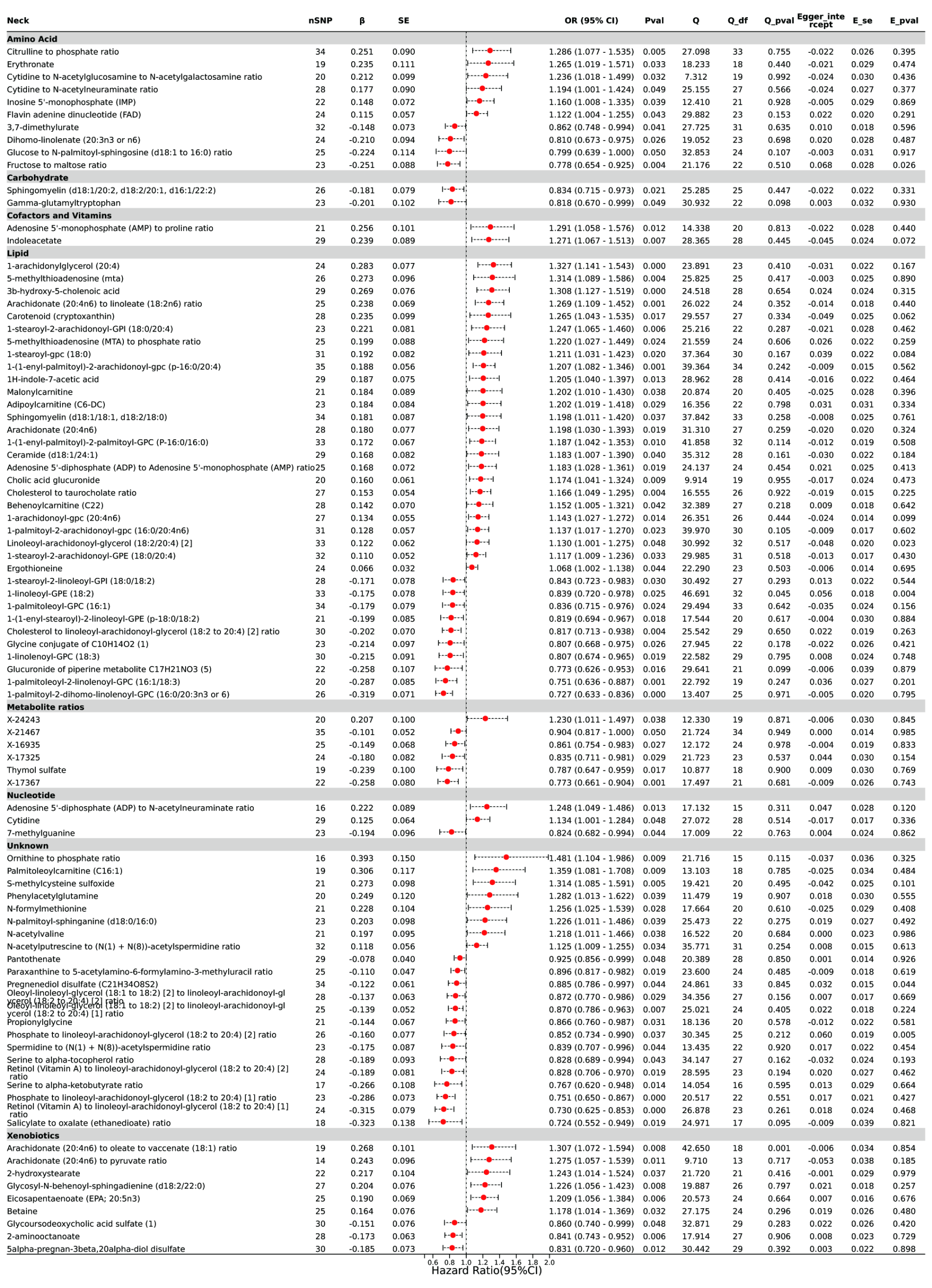


**Figure S14: Assessing MR causality between metabolites and fractures (neck).** Exposure comprises 1400 metabolites, while the outcome is defined as fractures (neck); nSNP: number of single nucleotide polymorphisms; method: inverse variance weighting; OR: odds ratio; CI: confidence interval. The odds ratio (OR) and confidence interval (CI) are calculated, with OR > 1 indicating that the exposure is a risk factor for the outcome, and OR < 1 suggesting it serves as a protective factor. Heterogeneity is analyzed using Q, with Q_df representing the degrees of freedom; a Q_pval < 0.05 indicates significant heterogeneity. The Egger_intercept is used for pleiotropy analysis, with E_se denoting the standard error. A p-value (E_pval) < 0.05 signifies the presence of pleiotropy.


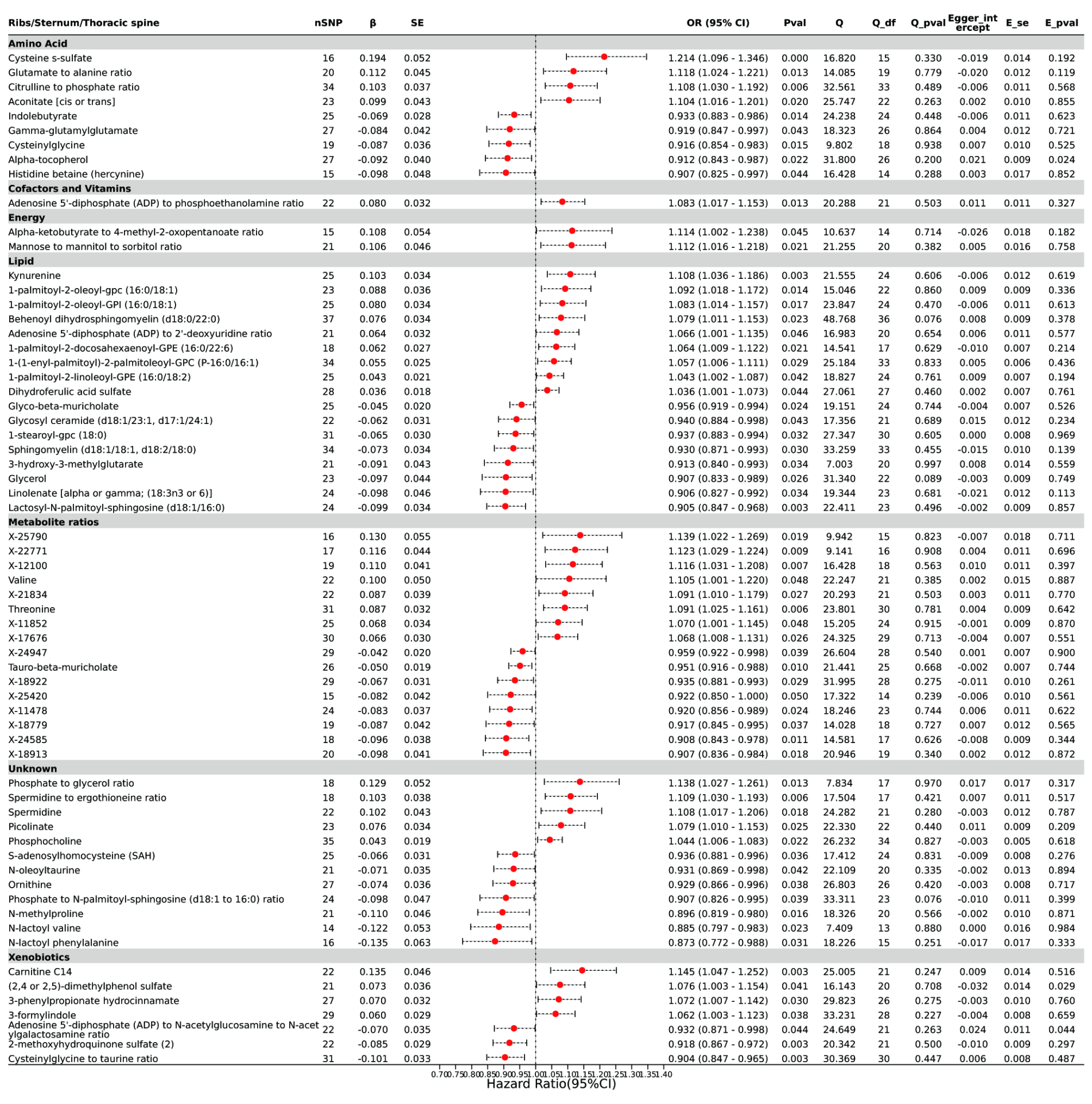


**Figure S15: Assessing MR causality between metabolites and fractures (ribs/sternum/thoracic spine).** Exposure comprises 1400 metabolites, while the outcome is defined as fractures (ribs/sternum/thoracic spine); nSNP: number of single nucleotide polymorphisms; method: inverse variance weighting; OR: odds ratio; CI: confidence interval. The odds ratio (OR) and confidence interval (CI) are calculated, with OR > 1 indicating that the exposure is a risk factor for the outcome, and OR < 1 suggesting it serves as a protective factor. Heterogeneity is analyzed using Q, with Q_df representing the degrees of freedom; a Q_pval < 0.05 indicates significant heterogeneity. The Egger_intercept is used for pleiotropy analysis, with E_se denoting the standard error. A p-value (E_pval) < 0.05 signifies the presence of pleiotropy.


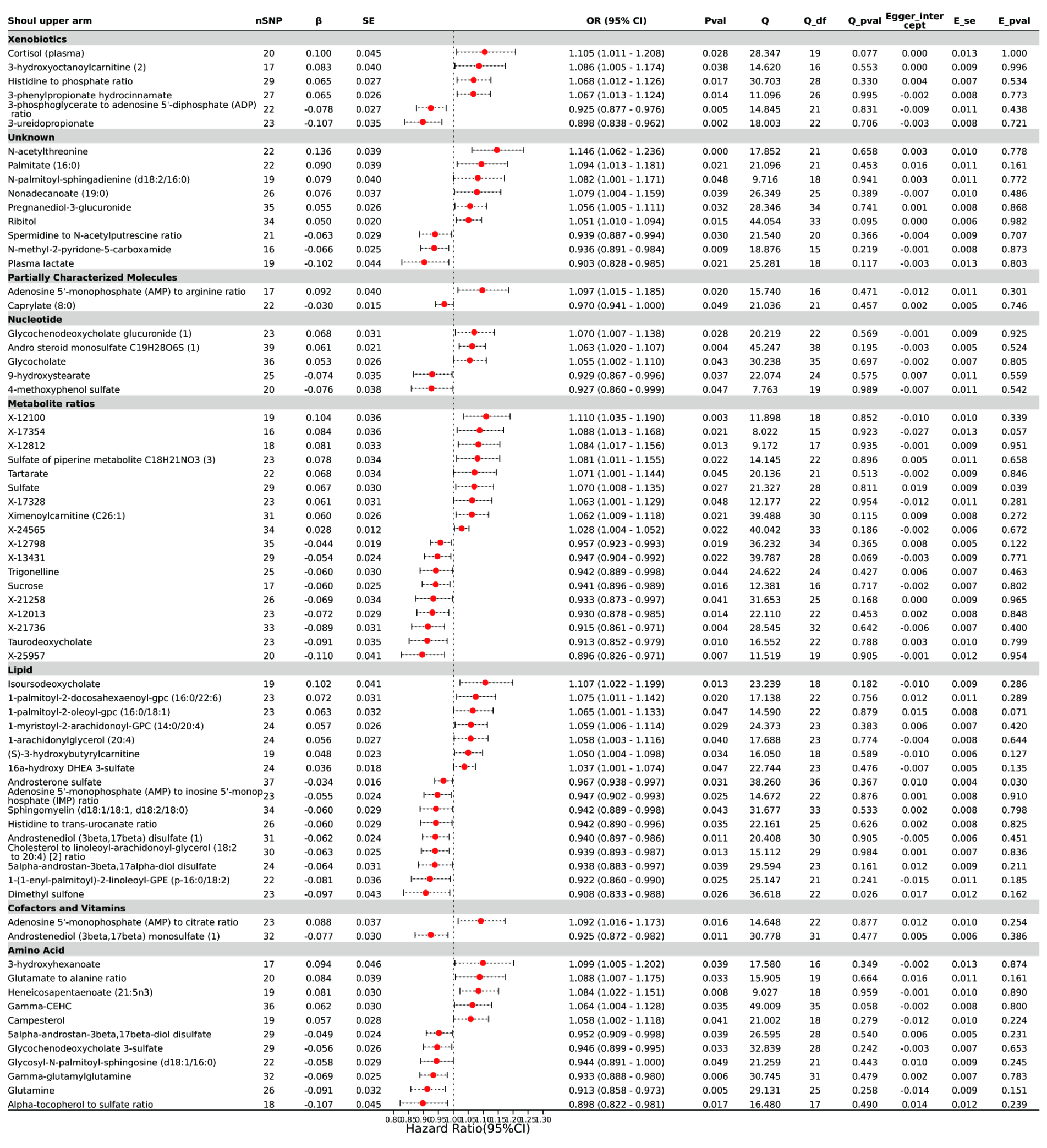


**Figure S16: Assessing MR causality between metabolites and fractures (shoul upper arm).** Exposure comprises 1400 metabolites, while the outcome is defined as fractures (shoul upper arm); nSNP: number of single nucleotide polymorphisms; method: inverse variance weighting; OR: odds ratio; CI: confidence interval. The odds ratio (OR) and confidence interval (CI) are calculated, with OR > 1 indicating that the exposure is a risk factor for the outcome, and OR < 1 suggesting it serves as a protective factor. Heterogeneity is analyzed using Q, with Q_df representing the degrees of freedom; a Q_pval < 0.05 indicates significant heterogeneity. The Egger_intercept is used for pleiotropy analysis, with E_se denoting the standard error. A p-value (E_pval) < 0.05 signifies the presence of pleiotropy.


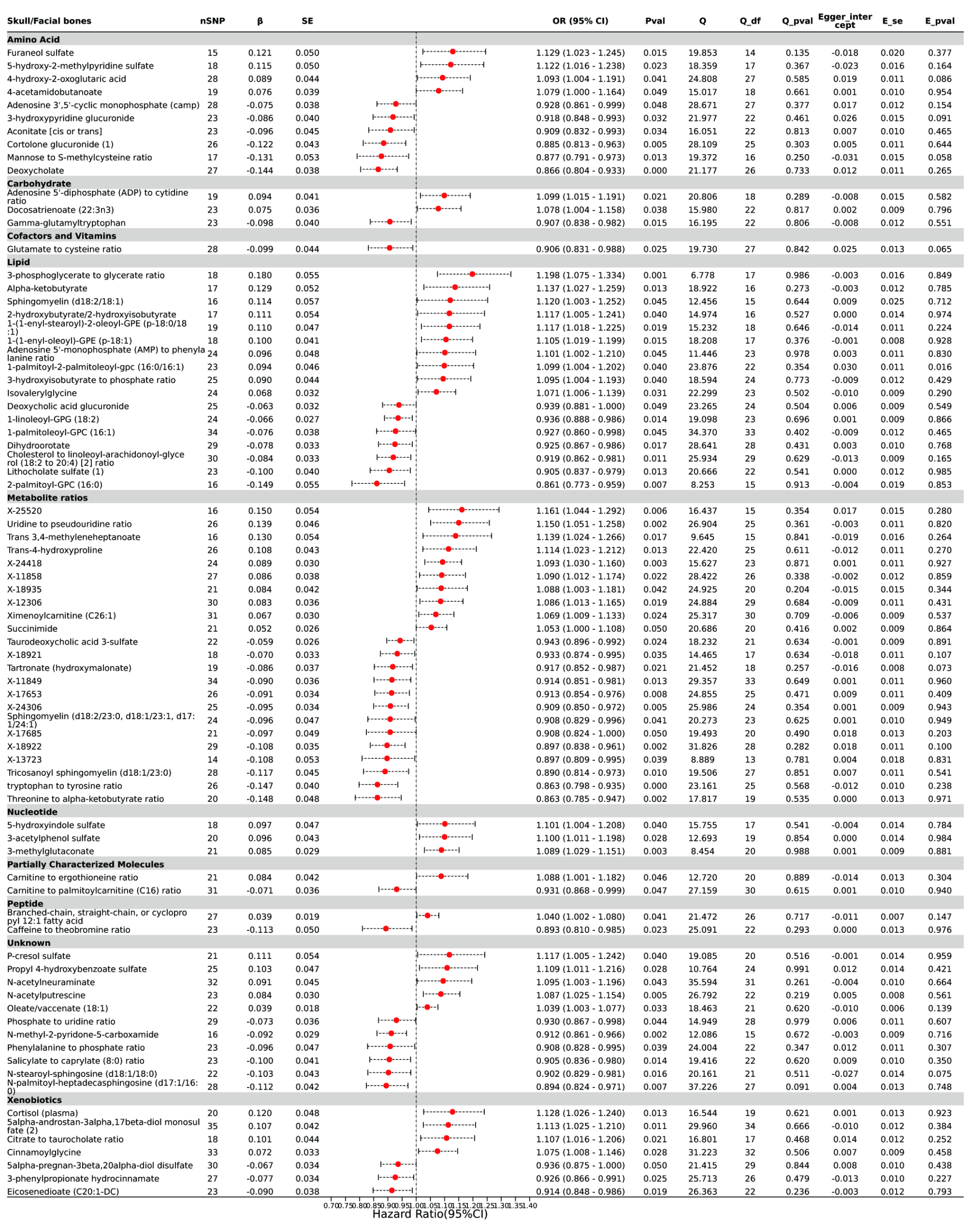


**Figure S17: Assessing MR causality between metabolites and fracture (skull/facial bones).** Exposure comprises 1400 metabolites, while the outcome is defined as fracture (skull/facial bones); nSNP: number of single nucleotide polymorphisms; method: inverse variance weighting；OR: odds ratio; CI: confidence interval. The odds ratio (OR) and confidence interval (CI) are calculated, with OR > 1 indicating that the exposure is a risk factor for the outcome, and OR < 1 suggesting it serves as a protective factor. Heterogeneity is analyzed using Q, with Q_df representing the degrees of freedom; a Q_pval < 0.05 indicates significant heterogeneity. The Egger_intercept is used for pleiotropy analysis, with E_se denoting the standard error. A P-value (E_pval) < 0.05 signifies the presence of pleiotropy.

**Figure S17: Assessing MR causality between metabolites and fractures (skull/facial bones).** Exposure comprises 1400 metabolites, while the outcome is defined as fractures (skull/facial bones); nSNP: number of single nucleotide polymorphisms; method: inverse variance weighting; OR: odds ratio; CI: confidence interval. The odds ratio (OR) and confidence interval (CI) are calculated, with OR > 1 indicating that the exposure is a risk factor for the outcome, and OR < 1 suggesting it serves as a protective factor. Heterogeneity is analyzed using Q, with Q_df representing the degrees of freedom; a Q_pval < 0.05 indicates significant heterogeneity. The Egger_intercept is used for pleiotropy analysis, with E_se denoting the standard error. A p-value (E_pval) < 0.05 signifies the presence of pleiotropy.


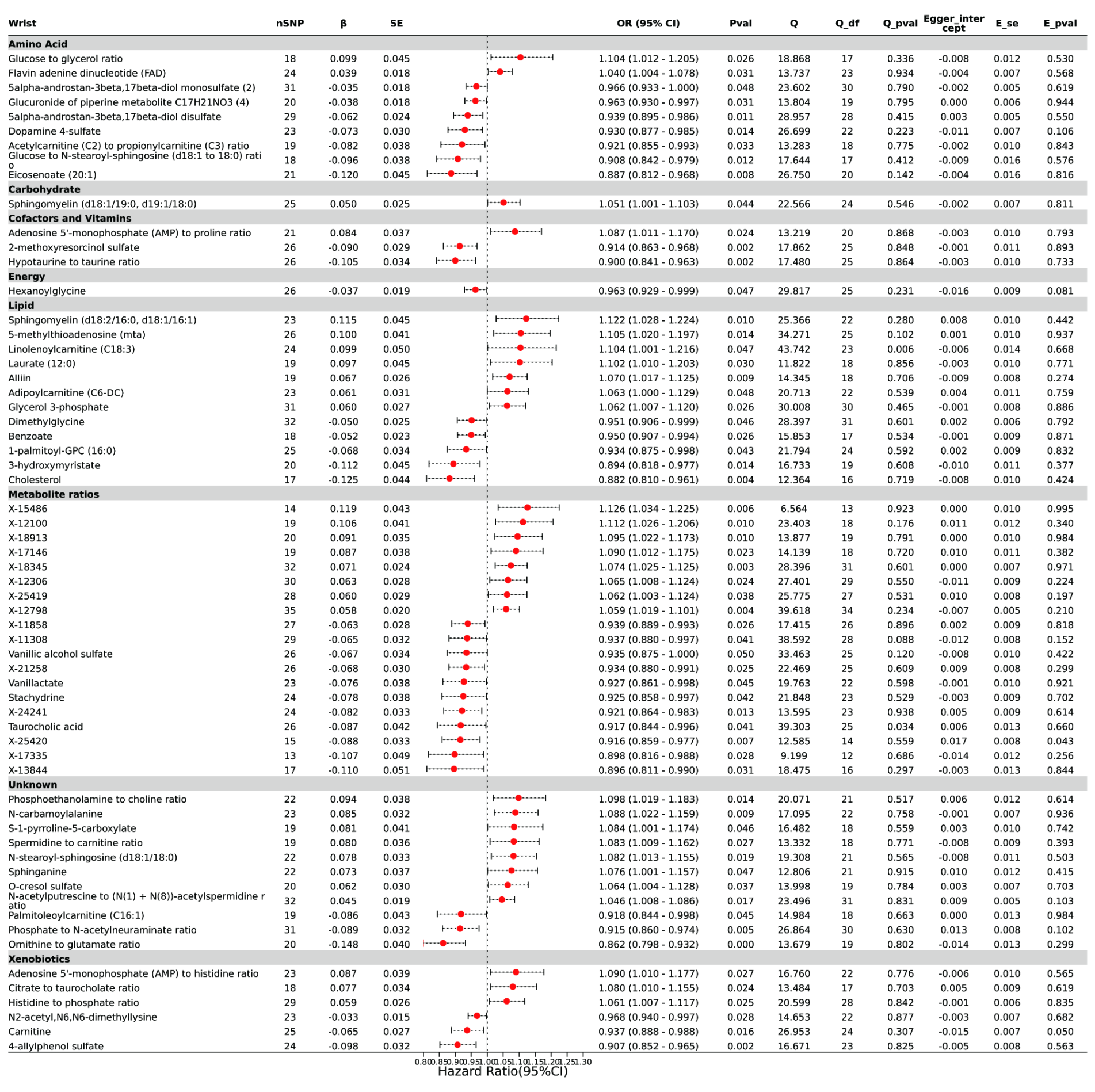


**Figure S18: Assessing MR causality between metabolites and fractures (wrist).** Exposure comprises 1400 metabolites, while the outcome is defined as fractures (wrist); nSNP: number of single nucleotide polymorphisms; method: inverse variance weighting; OR: odds ratio; CI: confidence interval. The odds ratio (OR) and confidence interval (CI) are calculated, with OR > 1 indicating that the exposure is a risk factor for the outcome, and OR < 1 suggesting it serves as a protective factor. Heterogeneity is analyzed using Q, with Q_df representing the degrees of freedom; a Q_pval < 0.05 indicates significant heterogeneity. The Egger_intercept is used for pleiotropy analysis, with E_se denoting the standard error. A p-value (E_pval) < 0.05 signifies the presence of pleiotropy.


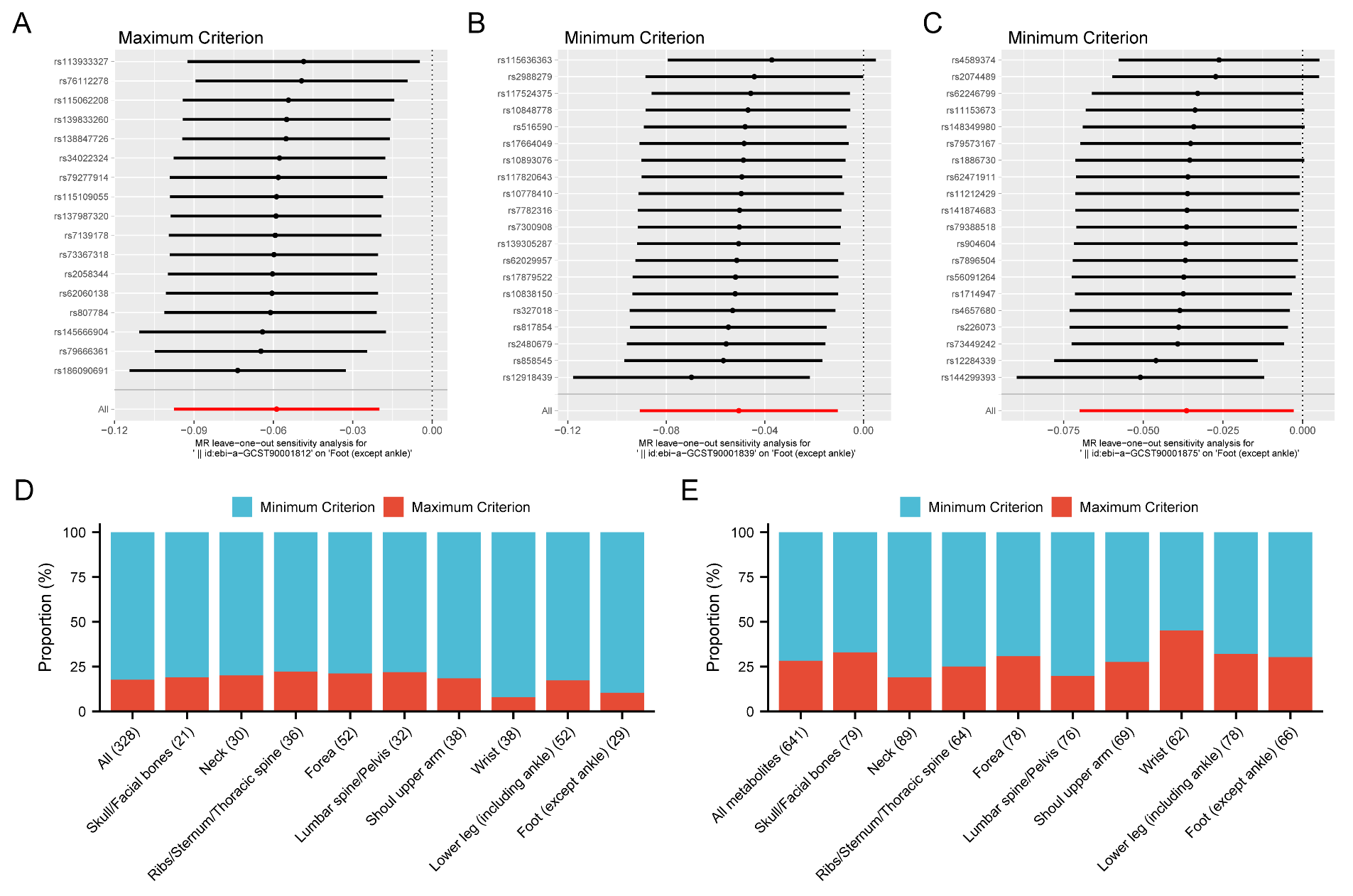


**Figure S19: Statistical results of leave-one-out (LOO) sensitivity analysis on immune cells and metabolites associated with fractures by MR analysis.** Figures A-C exemplify two scenarios in the current LOO sensitivity analysis: (A) Maximum Criterion: The LOO confidence intervals for all SNPs do not cross or include zero (the black vertical dashed line) and remain consistent (all on the same side). (B, C) Minimum Criterion: The LOO confidence intervals for most SNPs do not cross or include zero (the black vertical dashed line) and are consistent (all on the same side). The proportion of Minimum Criterion and Maximum Criterion in the LOO sensitivity analysis results was quantified for (D) immune cells and (E) metabolites associated with fractures in the Mendelian randomization analysis.


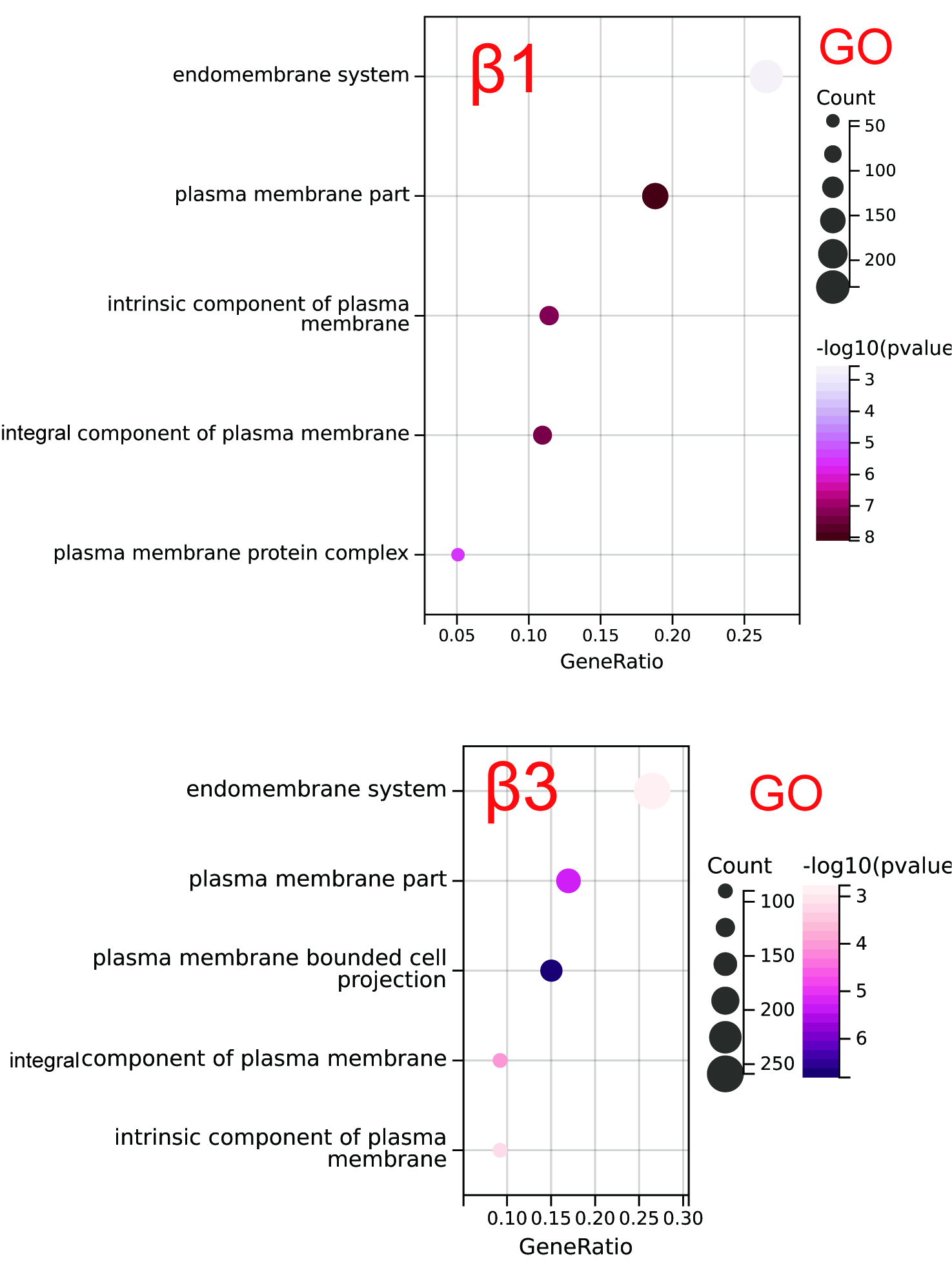


**Figure S20: Top 5 GO enrichment analysis of SNP-associated genes for MR of causality between immune cells/metabolites and fractures.** The bubble diagram (upper section) illustrates gene enrichment pathways for five common GO pathways in MR analysis of causal SNPs between immune cells and metabolites. The lower section of the figure demonstrates gene enrichment pathways for five common GO pathways in MR analysis of causal SNPs between immune cells and fractures. The β value represents the effect size of the SNP in two-sample MR. β1 represents the effect size of the SNP for immune cells and metabolites, and β3 represents the effect size of the SNP for metabolites and fractures. All pathways with p < 0.05 were considered statistically significant.


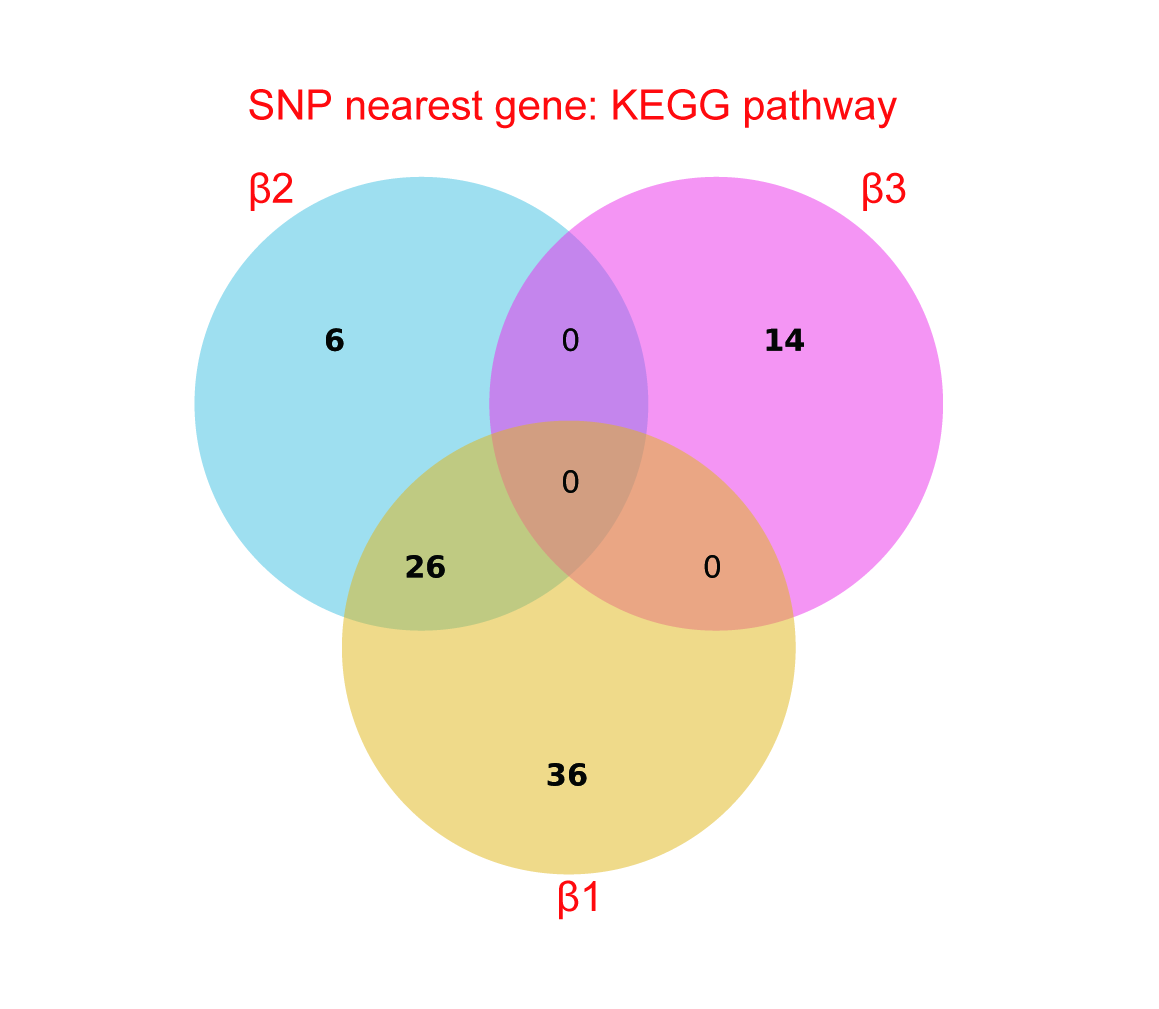


**Figure S21: KEGG pathway of genes from the association between immune cells, metabolites, and fractures.** Intersection of SNP-associated gene enrichment KEGG pathways obtained from three MR analyses. β1 represents the effect size of the SNP for immune cells and metabolites, β2 represents the effect size of the SNP for immune cells and fractures, and β3 represents the effect size of the SNP for metabolites and fractures. All pathways with p < 0.05 were considered statistically significant.


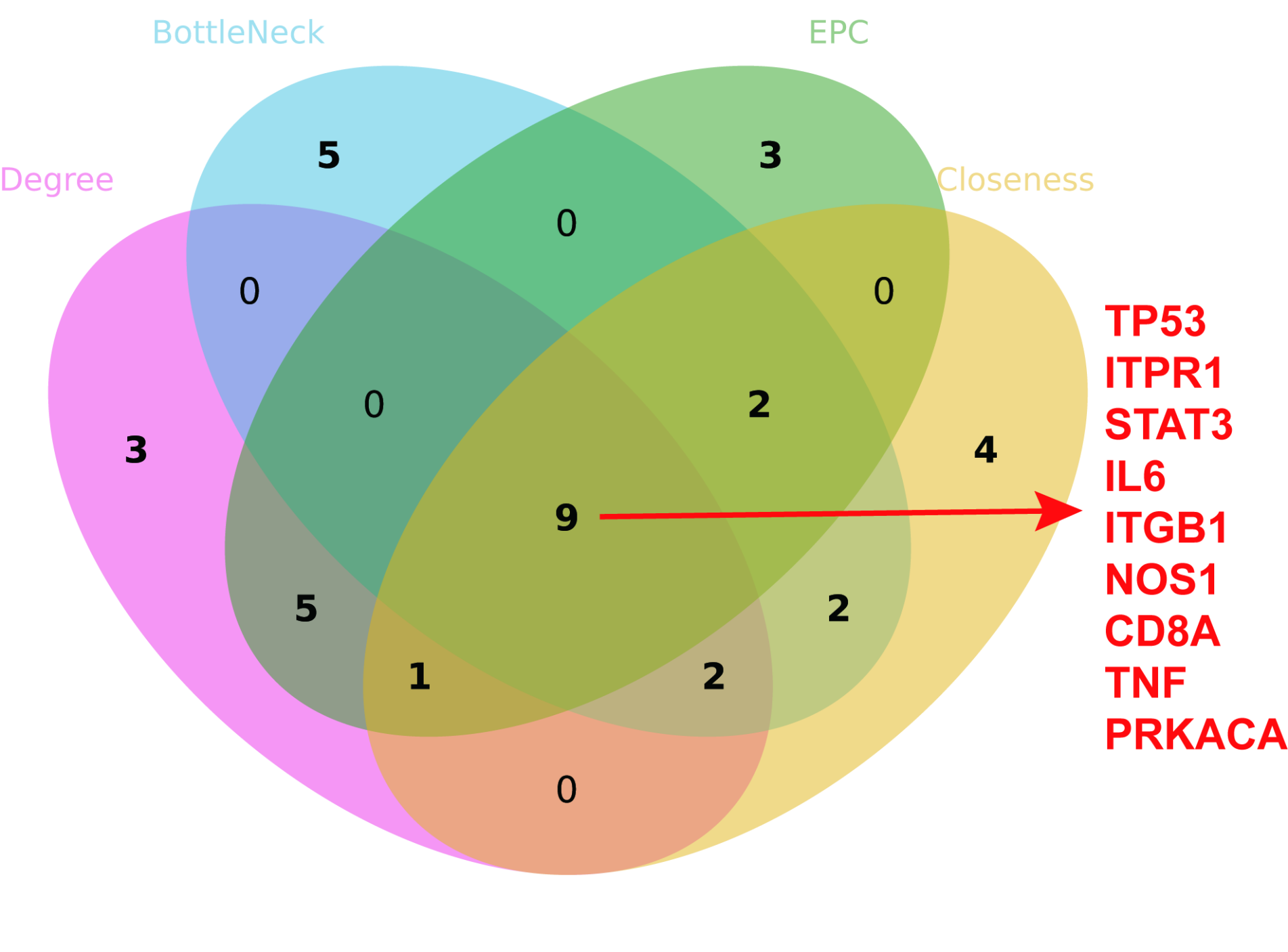


**Figure S22: PPI analysis of GO pathway-related genes.** The top 20 genes were assessed through four PPI analysis algorithms—Degree, EPC, Bottleneck, and Closeness—resulting in the identification of nine critical genes.


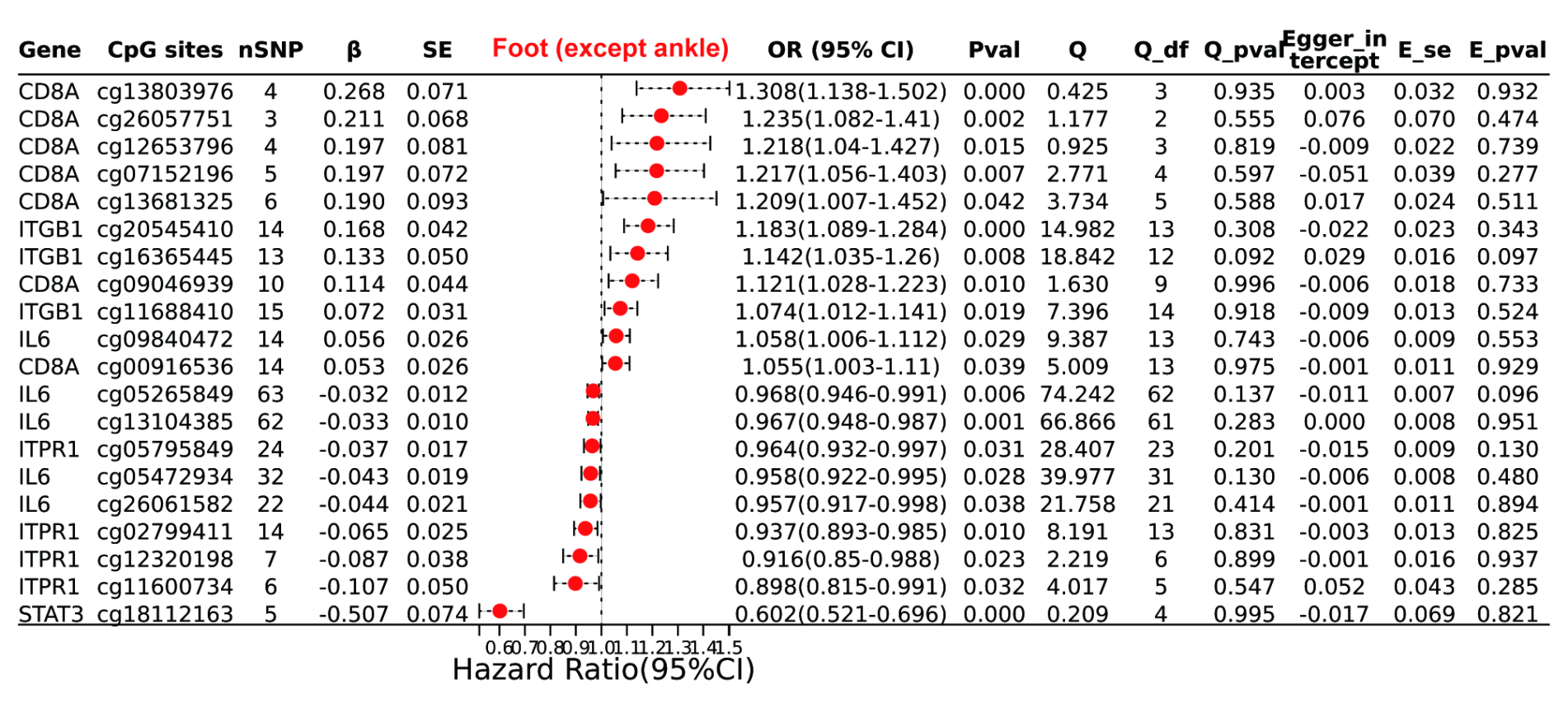


**Figure S23: Assessing MR causality between CpG sites of hub genes and fractures (foot, excluding ankle).** Exposure comprises CpG sites of hub genes, while the outcome is defined as fractures (foot, excluding ankle). nSNP: number of single nucleotide polymorphisms; method: inverse variance weighting; OR: odds ratio; CI: confidence interval. The odds ratio (OR) and confidence interval (CI) are calculated, with OR > 1 indicating that the exposure is a risk factor for the outcome, and OR < 1 suggesting it serves as a protective factor. Heterogeneity is analyzed using Q, with Q_df representing the degrees of freedom; a Q_pval < 0.05 indicates significant heterogeneity. The Egger_intercept is used for pleiotropy analysis, with E_se denoting the standard error. A P-value (E_pval) < 0.05 signifies the presence of pleiotropy.


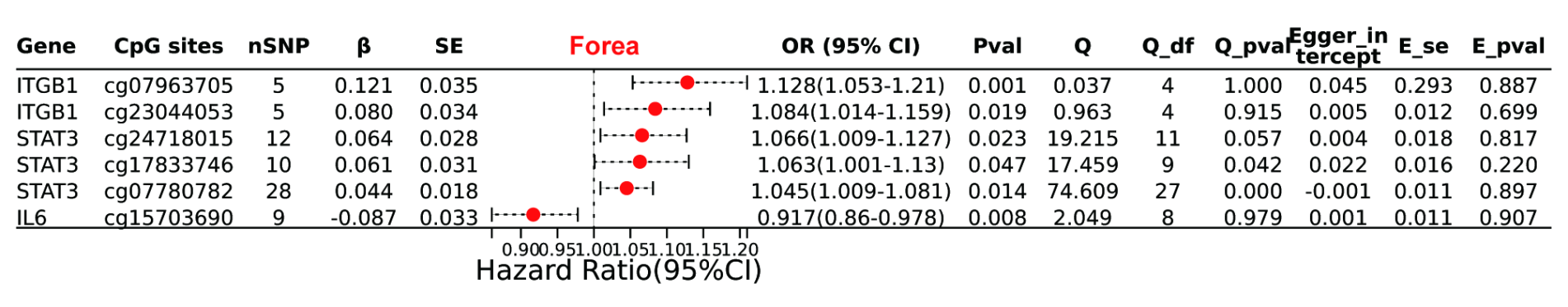


**Figure S24: Assessing MR causality between CpG sites of hub genes and fractures (forea).** Exposure comprises CpG sites of hub genes, while the outcome is defined as fractures (forea). nSNP: number of single nucleotide polymorphisms; method: inverse variance weighting; OR: odds ratio; CI: confidence interval. The odds ratio (OR) and confidence interval (CI) are calculated, with OR > 1 indicating that the exposure is a risk factor for the outcome, and OR < 1 suggesting it serves as a protective factor. Heterogeneity is analyzed using Q, with Q_df representing the degrees of freedom; a Q_pval < 0.05 indicates significant heterogeneity. The Egger_intercept is used for pleiotropy analysis, with E_se denoting the standard error. A P-value (E_pval) < 0.05 signifies the presence of pleiotropy.


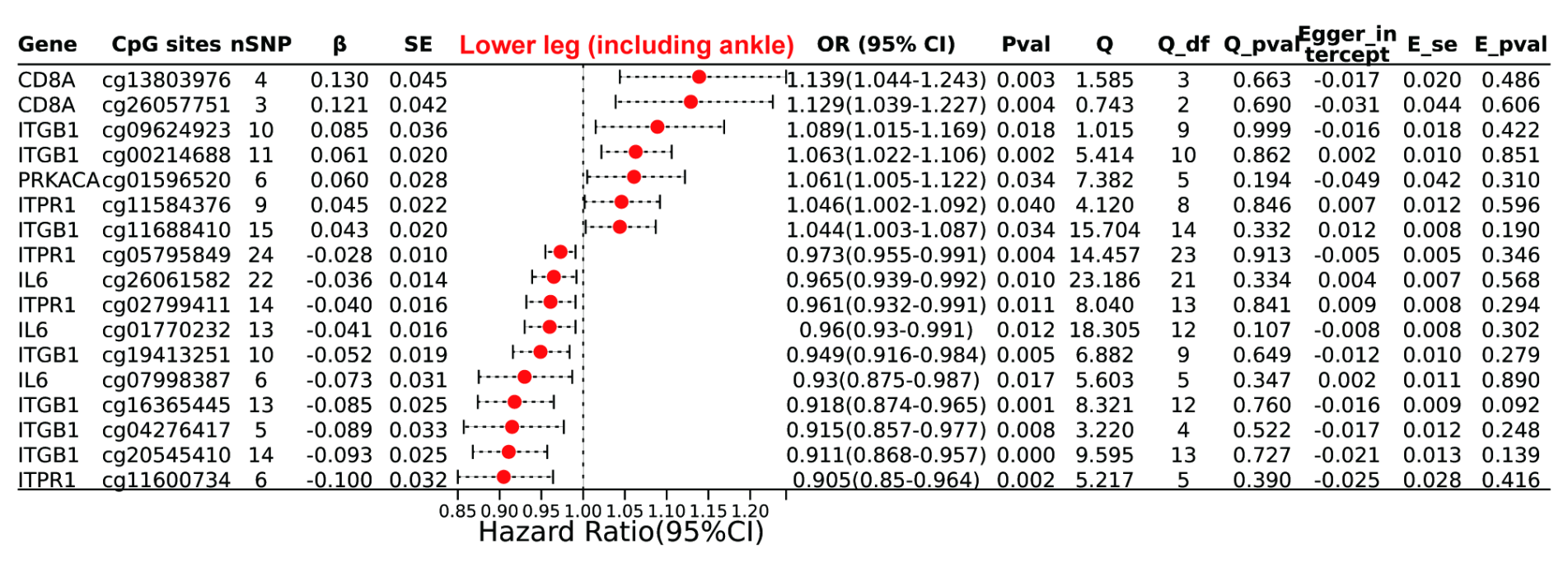


**Figure S25: Assessing MR causality between CpG sites of hub genes and fractures (lower leg, including ankle).** Exposure comprises CpG sites of hub genes, while the outcome is defined as fractures (lower leg, including ankle). nSNP: number of single nucleotide polymorphisms; method: inverse variance weighting; OR: odds ratio; CI: confidence interval. The odds ratio (OR) and confidence interval (CI) are calculated, with OR > 1 indicating that the exposure is a risk factor for the outcome, and OR < 1 suggesting it serves as a protective factor. Heterogeneity is analyzed using Q, with Q_df representing the degrees of freedom; a Q_pval < 0.05 indicates significant heterogeneity. The Egger_intercept is used for pleiotropy analysis, with E_se denoting the standard error. A P-value (E_pval) < 0.05 signifies the presence of pleiotropy.


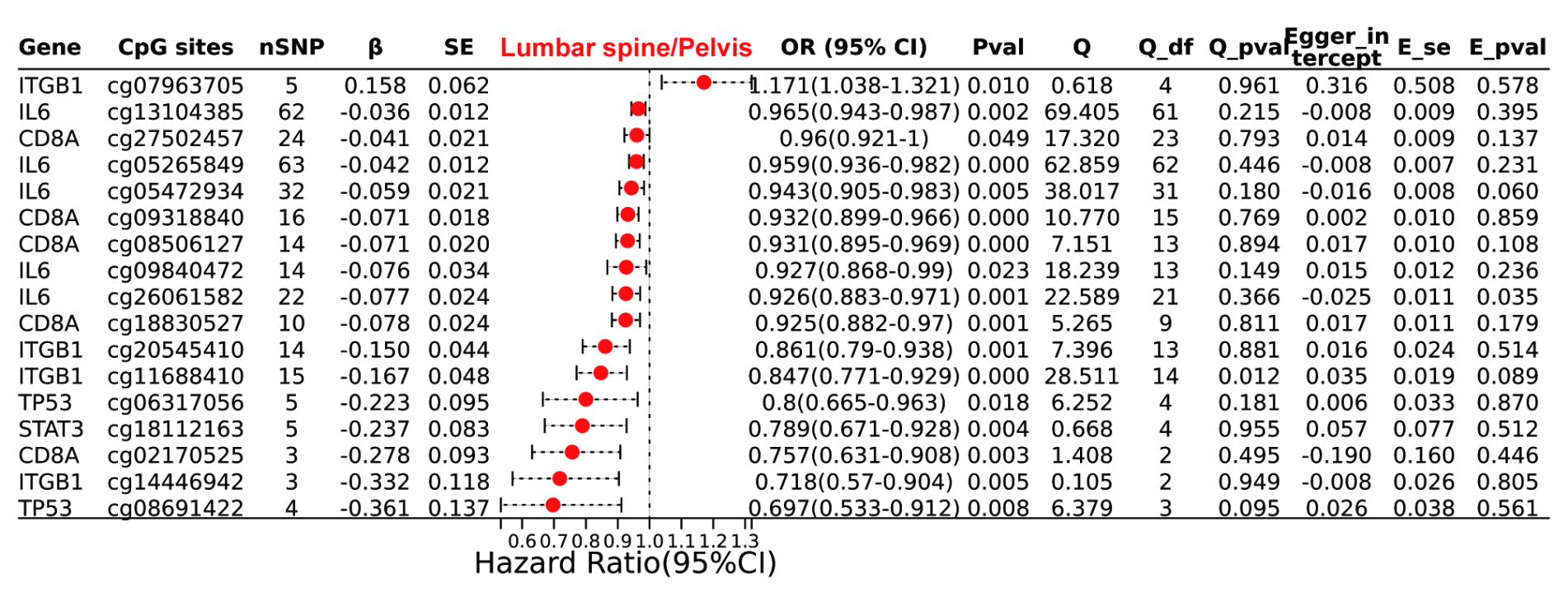


**Figure S26: Assessing MR causality between CpG sites of hub genes and fractures (lumbar spine/pelvis).** Exposure comprises CpG sites of hub genes, while the outcome is defined as fractures (lumbar spine/pelvis). nSNP: number of single nucleotide polymorphisms; method: inverse variance weighting; OR: odds ratio; CI: confidence interval. The odds ratio (OR) and confidence interval (CI) are calculated, with OR > 1 indicating that the exposure is a risk factor for the outcome, and OR < 1 suggesting it serves as a protective factor. Heterogeneity is analyzed using Q, with Q_df representing the degrees of freedom; a Q_pval < 0.05 indicates significant heterogeneity. The Egger_intercept is used for pleiotropy analysis, with E_se denoting the standard error. A P-value (E_pval) < 0.05 signifies the presence of pleiotropy.


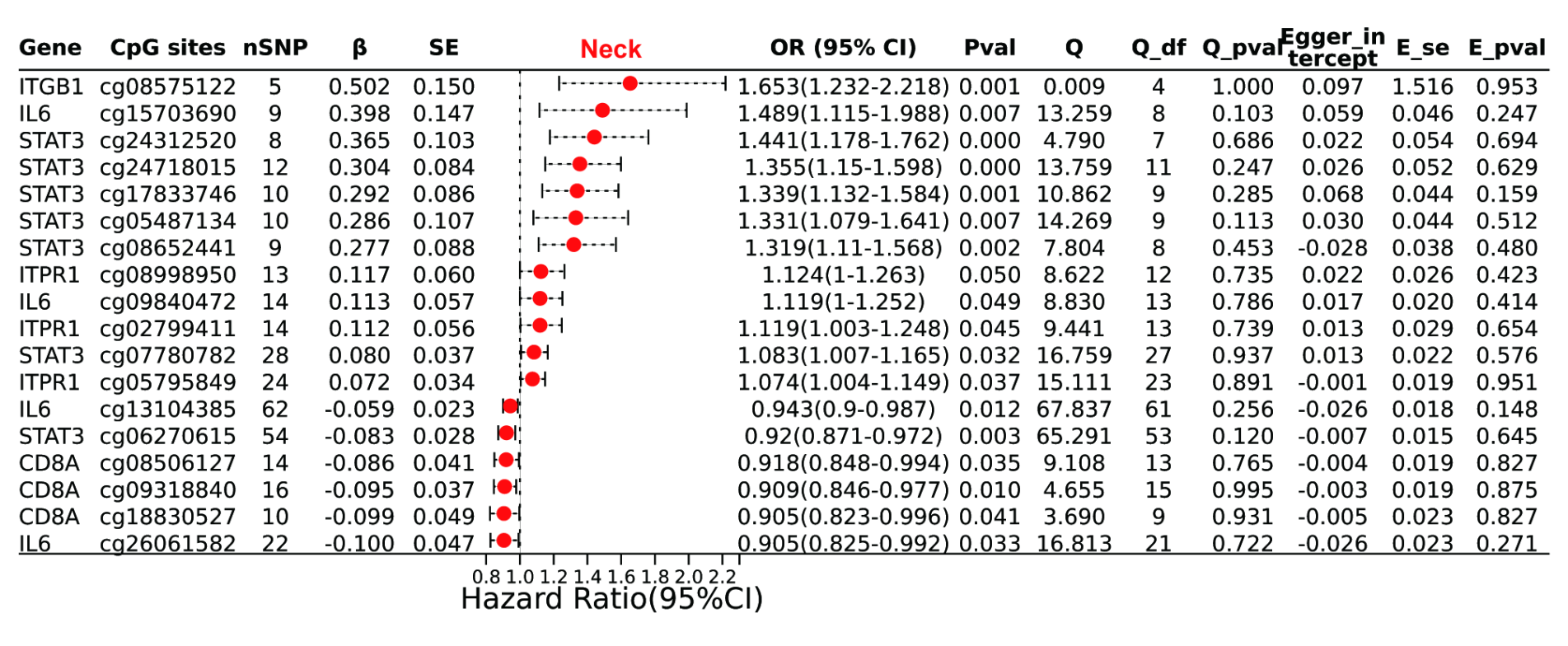


**Figure S27: Assessing MR causality between CpG sites of hub genes and fractures (neck).** Exposure comprises CpG sites of hub genes, while the outcome is defined as fractures (neck). nSNP: number of single nucleotide polymorphisms; method: inverse variance weighting; OR: odds ratio; CI: confidence interval. The odds ratio (OR) and confidence interval (CI) are calculated, with OR > 1 indicating that the exposure is a risk factor for the outcome, and OR < 1 suggesting it serves as a protective factor. Heterogeneity is analyzed using Q, with Q_df representing the degrees of freedom; a Q_pval < 0.05 indicates significant heterogeneity. The Egger_intercept is used for pleiotropy analysis, with E_se denoting the standard error. A P-value (E_pval) < 0.05 signifies the presence of pleiotropy.


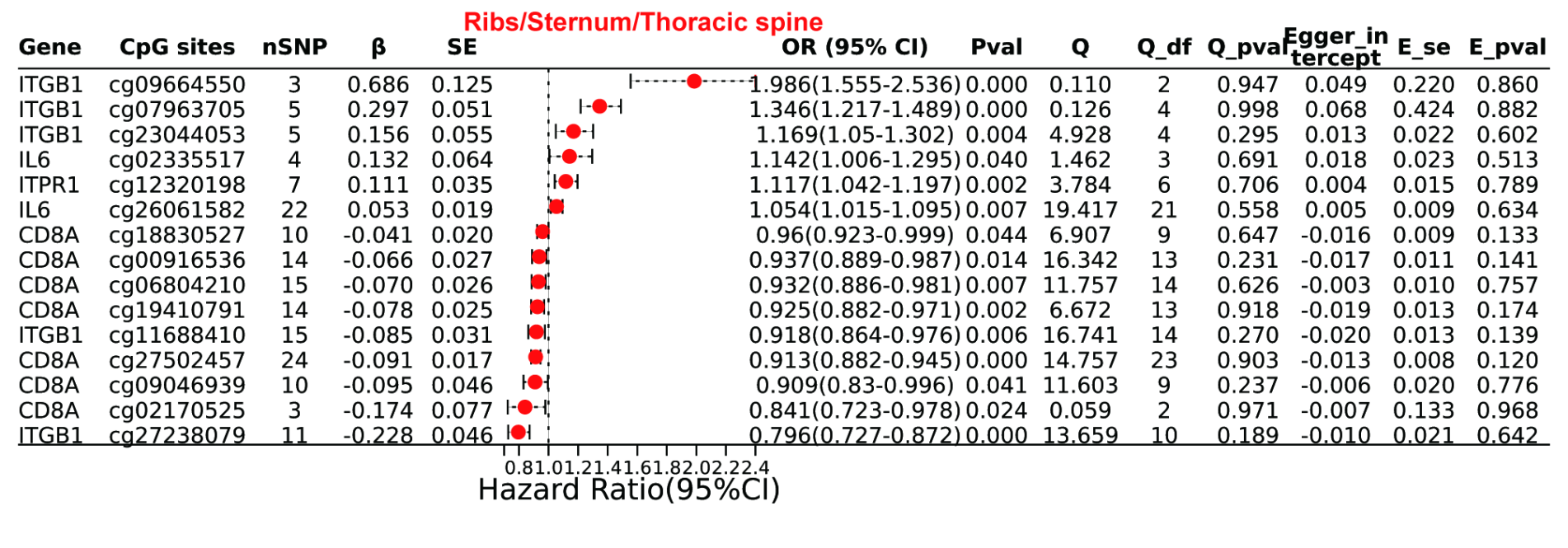


**Figure S28: Assessing MR causality between CpG sites of hub genes and fractures (ribs/sternum/thoracic spine).** Exposure comprises CpG sites of hub genes, while the outcome is defined as fractures (ribs/sternum/thoracic spine). nSNP: number of single nucleotide polymorphisms; method: inverse variance weighting; OR: odds ratio; CI: confidence interval. The odds ratio (OR) and confidence interval (CI) are calculated, with OR > 1 indicating that the exposure is a risk factor for the outcome, and OR < 1 suggesting it serves as a protective factor. Heterogeneity is analyzed using Q, with Q_df representing the degrees of freedom; a Q_pval < 0.05 indicates significant heterogeneity. The Egger_intercept is used for pleiotropy analysis, with E_se denoting the standard error. A P-value (E_pval) < 0.05 signifies the presence of pleiotropy.


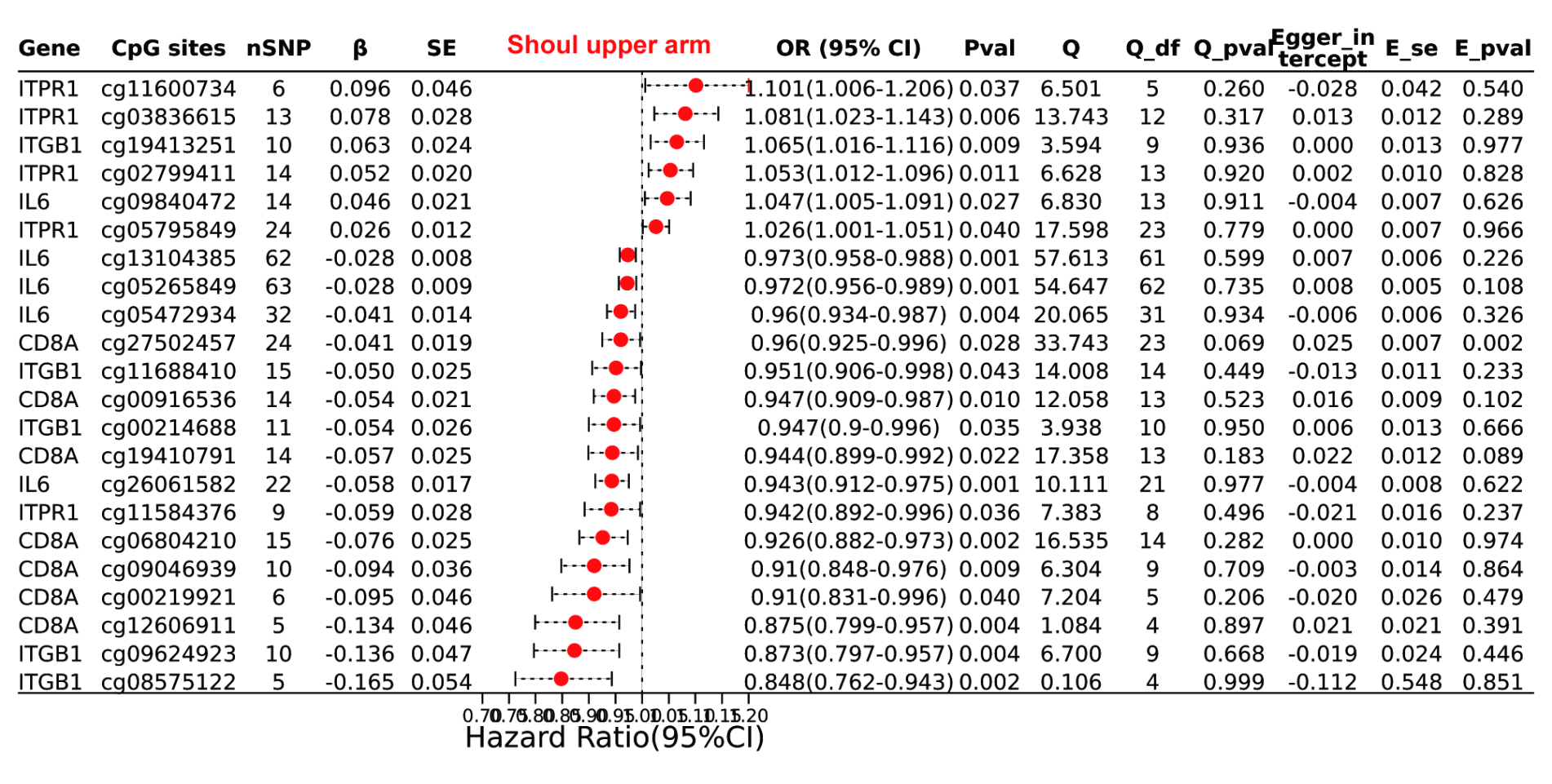


**Figure S29: Assessing MR causality between CpG sites of hub genes and fractures (shoul upper arm).** Exposure comprises CpG sites of hub genes, while the outcome is defined as fractures (shoul upper arm). nSNP: number of single nucleotide polymorphisms; method: inverse variance weighting; OR: odds ratio; CI: confidence interval. The odds ratio (OR) and confidence interval (CI) are calculated, with OR > 1 indicating that the exposure is a risk factor for the outcome, and OR < 1 suggesting it serves as a protective factor. Heterogeneity is analyzed using Q, with Q_df representing the degrees of freedom; a Q_pval < 0.05 indicates significant heterogeneity. The Egger_intercept is used for pleiotropy analysis, with E_se denoting the standard error. A P-value (E_pval) < 0.05 signifies the presence of pleiotropy.

**
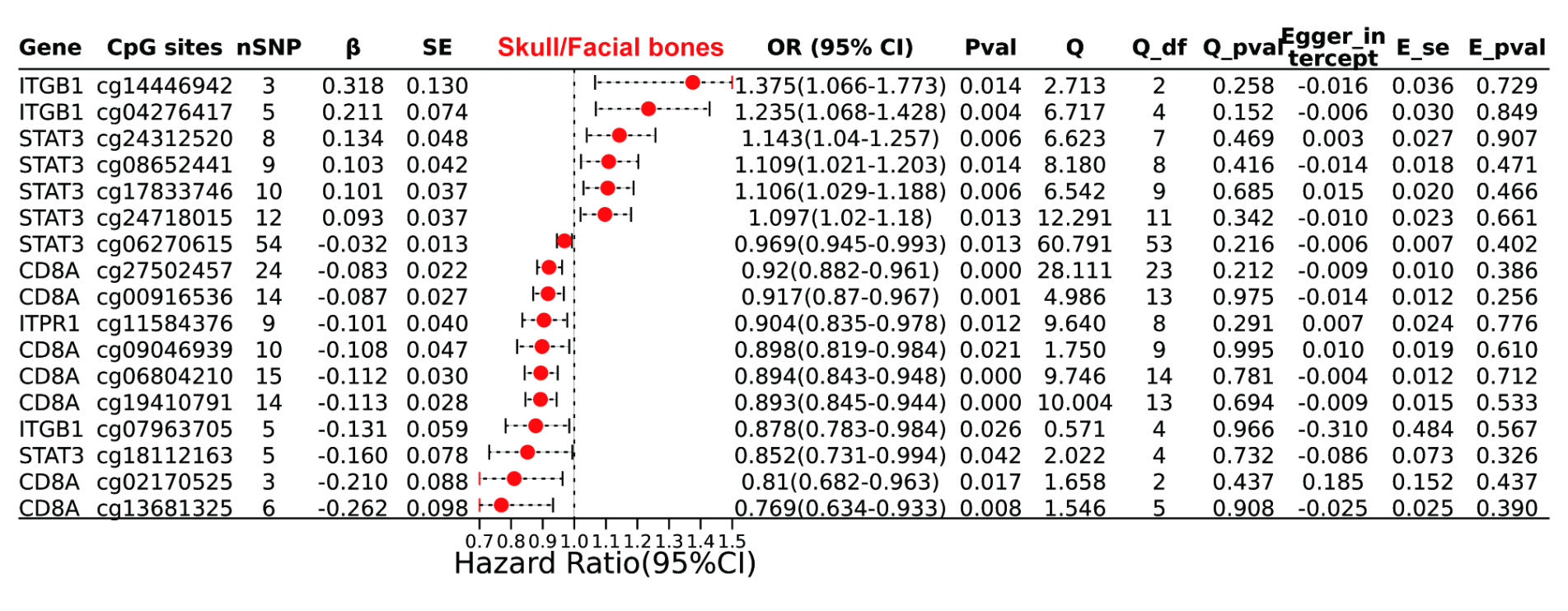
**

**Figure S30: Assessing MR causality between CpG sites of hub genes and fractures (skull/facial bones).** Exposure comprises CpG sites of hub genes, while the outcome is defined as fractures (skull/facial bones). nSNP: number of single nucleotide polymorphisms; method: inverse variance weighting; OR: odds ratio; CI: confidence interval. The odds ratio (OR) and confidence interval (CI) are calculated, with OR > 1 indicating that the exposure is a risk factor for the outcome, and OR < 1 suggesting it serves as a protective factor. Heterogeneity is analyzed using Q, with Q_df representing the degrees of freedom; a Q_pval < 0.05 indicates significant heterogeneity. The Egger_intercept is used for pleiotropy analysis, with E_se denoting the standard error. A P-value (E_pval) < 0.05 signifies the presence of pleiotropy.


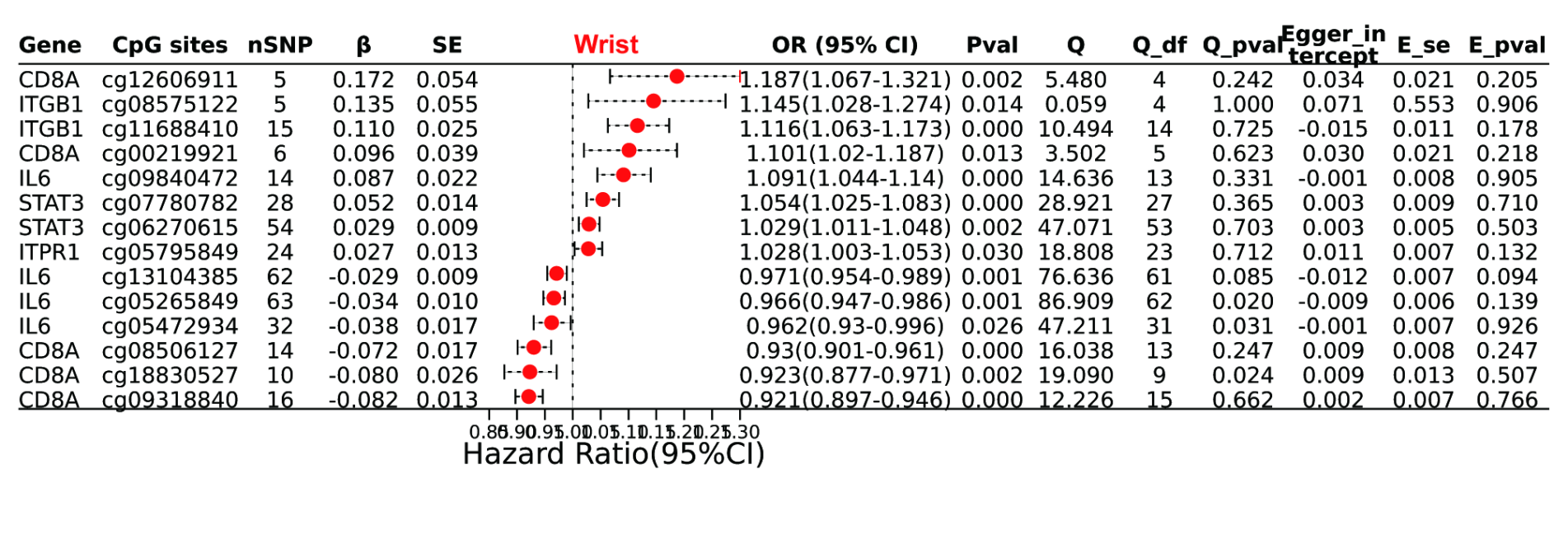


**Figure S31: Assessing MR causality between CpG sites of hub genes and fractures (wrist).** Exposure comprises CpG sites of hub genes, while the outcome is defined as fractures (wrist). nSNP: number of single nucleotide polymorphisms; method: inverse variance weighting; OR: odds ratio; CI: confidence interval. The odds ratio (OR) and confidence interval (CI) are calculated, with OR > 1 indicating that the exposure is a risk factor for the outcome, and OR < 1 suggesting it serves as a protective factor. Heterogeneity is analyzed using Q, with Q_df representing the degrees of freedom; a Q_pval < 0.05 indicates significant heterogeneity. The Egger_intercept is used for pleiotropy analysis, with E_se denoting the standard error. A P-value (E_pval) < 0.05 signifies the presence of pleiotropy.
